# Altered fecal microbiome and metabolome in adult patients with non-cystic fibrosis bronchiectasis

**DOI:** 10.1186/s12931-022-02229-w

**Published:** 2022-11-19

**Authors:** Wen-Wen Wang, Bei Mao, Yang Liu, Shu-Yi Gu, Hai-Wen Lu, Jiu-Wu Bai, Shuo Liang, Jia-Wei Yang, Jian-Xiong Li, Xiao Su, Hai-Yang Hu, Chen Wang, Jin-Fu Xu

**Affiliations:** 1 Department of Respiratory and Critical Care Medicine, Shanghai Pulmonary Hospital, Institute of Respiratory Medicine, Tongji University School of Medicine, 200433 Shanghai, China; 2 Unit of Respiratory Infection and Immunity, Institute Pasteur of Shanghai, Chinese Academy of Sciences, 200031 Shanghai, China; 3 State Key Laboratory of Natural Medicines, School of Life Science and Technology, China Pharmaceutical University, 211198 Nanjing, China

**Keywords:** Biomarkers, Bronchiectasis, Gut microbiome, Metabolomics

## Abstract

**Background:**

Emerging experimental and epidemiological evidence highlights a crucial cross-talk between the intestinal flora and the lungs, termed the “gut-lung axis”. However, the function of the gut microbiota in bronchiectasis remains undefined. In this study, we aimed to perform a multi-omics-based approach to identify the gut microbiome and metabolic profiles in patients with bronchiectasis.

**Methods:**

Fecal samples collected from non-CF bronchiectasis patients (BE group, n = 61) and healthy volunteers (HC group, n = 37) were analyzed by 16 S ribosomal RNA (rRNA) sequencing. The BE group was divided into two groups based on their clinical status: acute exacerbation (AE group, n = 31) and stable phase (SP group, n = 30). Further, metabolome (lipid chromatography-mass spectrometry, LC-MS) analyses were conducted in randomly selected patients (n = 29) and healthy volunteers (n = 31).

**Results:**

Decreased fecal microbial diversity and differential microbial and metabolic compositions were observed in bronchiectasis patients. Correlation analyses indicated associations between the differential genera and clinical parameters such as bronchiectasis severity index (BSI). Disease-associated gut microbiota was screened out, with eight genera exhibited high accuracy in distinguishing SP patients from HCs in the discovery cohort and validation cohort using a random forest model. Further correlation networks were applied to illustrate the relations connecting disease-associated genera and metabolites.

**Conclusion:**

The study uncovered the relationships among the decreased fecal microbial diversity, differential microbial and metabolic compositions in bronchiectasis patients by performing a multi-omics-based approach. It is the first study to characterize the gut microbiome and metabolome in bronchiectasis, and to uncover the gut microbiota’s potentiality as biomarkers for bronchiectasis.

*Trial registration: *This study is registered with ClinicalTrials.gov, number NCT04490447.

**Supplementary Information:**

The online version contains supplementary material available at 10.1186/s12931-022-02229-w.

## Backgrounds

Non-CF bronchiectasis is the third most common chronic airway disease [1], after asthma and chronic obstructive pulmonary disease (COPD), and with the highest prevalence in Asian populations [1, 2]. It is characterized by abnormal and irreversible bronchus dilation, causing recurrent respiratory infections, cough, and sputum production [3], imposing a substantial economic burden on healthcare [4]. The estimated prevalence in Chinese over 40 years of age is 1.2%, and the etiologies of over 70% of patients in China remain unclarified due to its great heterogeneity [5]. There are currently no approved therapies for bronchiectasis, and even the evidence supporting widely applied therapies is limited [6].

Gut microbiota plays an essential role in the development of the immune system and may contribute to the metabolic homeostasis of the human host [7–10], Emerging evidence has revealed the strong impact of the gut microbiota and the related “gut-lung axis” on the modulation of immune responses and disease development in the lungs [11–14], indicating the potential for modulation of gut microbiota for treating pulmonary diseases. Studies have characterized the gut microbiome of other chronic respiratory diseases, including asthma and cystic fibrosis (CF) [15–17], and several factors associated with COPD cause dysbiosis of the gut microbiome [18, 19]. Nevertheless, the gut microbiome and metabolome of bronchiectasis remain unclear. We hypothesized that patients with bronchiectasis exhibited different gut microbiome and metabolic profiles from healthy people.

In this study, we recruited 61 bronchiectasis patients and 37 healthy volunteers in the discovery cohort. Fecal samples were collected for subsequent 16 S ribosomal RNA (rRNA) Miseq gene sequencing and metabolomics analysis. The shifted gut microbial spectrum and metabolome of bronchiectasis patients were disclosed, so did the correlation between clinical parameters and relative abundance of microbial genera. A RF model was trained to assess the gut microbiota’s potential as noninvasive biomarkers for bronchiectasis. The composition of eight variant genera exhibited a high accuracy in the discrimination of bronchiectasis patients from HCs both in the discovery cohort and validation cohort.

## Methods

### Subject recruitment and sample collection

Ninety-two bronchiectasis patients and 59 healthy volunteers were recruited from the Shanghai Pulmonary Hospital. Part of patients were recommended from the hospital members of Bronchiectasis Treatment and Research Alliance of China (BEChina, http://www.chinabronchiectasis.com/). Eventually 58 healthy volunteers and 87 patients were included due to that 1 healthy volunteer and 5 patients did not meet the inclusion and exclusion criteria. All subjects were Han Chinese from the Yangtze River Delta region in China with similar eating habits. All included bronchiectasis patients meet the diagnostic criteria of the “British Thoracic Society guideline for non-cystic fibrosis (CF) bronchiectasis" [20]. All included healthy volunteers did not receive antibiotics or probiotics in the previous one month [21]. Further detailed inclusion and exclusion criteria for subjects’ enrollment were listed in Additional file 1: Table S1. Clinically stable patients with bronchiectasis who had no signs of exacerbation and did not receive antibiotics neither systemic nor nebulized in the previous one month were assigned to the SP group. Patients who had an acute exacerbation and requiring antibiotic treatment were assigned to the AE group, referring to the consensus definition of pulmonary exacerbation in adults with bronchiectasis. in brief, patients with a deterioration in three or more of the key symptoms including cough, sputum volume and/or consistency, sputum purulence, breathlessness and/or exercise tolerance, fatigue and/or malaise, hemoptysis, and needing a changed bronchiectasis treatment were considered suffering from a pulmonary exacerbation [22]. A total of 145 fecal samples were collected from the above participants. Each fecal sample was divided into three aliquots and immediately stored at − 80℃ as described [23]. After data quality control, samples were randomly assigned to the discovery cohort and the validation cohort. For the discovery cohort, 30, 31, and 37 fecal samples were collected from SP, AE, and HC groups, respectively, and assigned to 16 S rRNA gene sequencing. Seventeen and 19 fecal samples separately from HC and SP groups in the validation cohort were subjected to 16 S rRNA gene sequencing to validate the discovery cohort’s findings. Further metabolomic analyses (lipid chromatography-mass spectrometry, LC-MS) were conducted in the randomly selected SP group (n = 29) and HC group (n = 31).

### DNA extraction and 16 S rRNA gene sequencing of gut microbiota from bronchiectasis patients

DNA extraction was performed using the QIAamp Fast DNA Stool Mini Kit (Qiagen, Germany), referring to the manufacturer’s guidelines. DNA size was verified by 1% agarose gel electrophoresis, and its concentration was measured by NanoDrop 2000 (Thermo Scientific, Waltham, MA, USA). The V3-V4 hypervariable region of the bacterial 16S ribosomal RNA (rRNA) gene was amplified with the primers 341F (5’-CCTACGGGRSGCAGCAG-3’) and 806R (5’- GGACTACVVGGGTATCTAATC-3’). After purification, the amplicons were quantified, pooled to equalize concentrations, and sequenced using an Illumina Miseq PE250 platform (Illumina, California, USA).

### Sequencing data analysis of bronchiectasis patients’ gut microbiota

Sequences with more than 97% similarity thresholds were allocated to one of the described Operational Taxonomic Units (OTUs) using UPARSE (http://drive5.com/uparse/), and chimeric sequences were filtered using Usearch V.7.0.1090. The taxonomy was analyzed by the Ribosomal Database Project (RDP) Classifier algorithm (http://rdp.cme.msu.edu/) against the RDP database (http://rdp.cme.msu.edu/) with the confidence threshold of 0.8. The OTU profiling table, and α and β diversity were obtained using python scripts in QIIME V1.9.1. α diversity was estimated by the chao1 index and observed species index. β diversity was calculated using the unweighted UniFrac measure.

### Gut microbial biomarker identification based on random forest model

Linear discriminant analysis (LDA) values were obtained using LDA Effect Size (LEfSe) analysis [24], and the genera with LDA score (log10) larger than two were selected for subsequent random forest (RF) analyses. According to the descending order of mean decrease accuracy (MDA), the top 10 genera with the highest MDA values were chosen as candidates. They were iteratively eliminated to identify a set of combinations to train the forest with the highest accuracy for patients’ discrimination. Receiver operating characteristic (ROC) curves were established, and the values of the AUC were calculated to determine the discriminatory capacity of the remaining candidates. Combinations with the highest AUC value used to train the forest were included in the final model.

### LC-MS-based metabolomics data analysis for SP and HC

Fecal samples were extracted for the metabolite profile evaluation as previously described [25]. LC-MS/MS analyses were performed using a Ultra-High Performance Liquid Chromatography (UHPLC) system (Vanquish, Thermo Fisher Scientific) with a UPLC BEH amide column (2.1 mm × 100 mm, 1.7 μm) coupled to a Q Exactive HFX mass spectrometer (Orbitrap MS, Thermo Fisher Scientific). The mobile phase consisted of 25 mmol/L ammonium acetate and 25 mmol/L ammonia hydroxide in water (pH = 9.75) (A) and acetonitrile (B). The analyses were carried with elution gradient with column temperature at 30℃. The auto-sampler temperature was 4℃, and the injection volume was 3 µL. A Thermo Q Exactive HFX mass spectrometer was used to acquire MS/MS spectra on information-dependent acquisition (IDA) mode under the control of acquisition software (Xcalibur, Thermo Fisher Scientific). In this mode, the acquisition software continuously evaluated the full scan MS spectrum. The LC-MS raw data obtained from the platform were performed peak identification, extraction, alignment and integration as previously described [25].

### Statistical analysis

For clinical characteristics, differences of age, BMI, FEV1% predicted, BSI score, SGRQ score between groups were compared using the two-tailed Student’s t-test or Mann–Whitney U test based on the normality test or Levene’s test. Differences of gender, smoking status, medication, pathogen infection between groups were calculated using Pearson’s chi-square test or Fisher’s exact test. The correlation between the clinical parameters was evaluated using Pearson’s correlation analysis. Fecal microbial characterization was determined by LEfSe with Wilcoxon rank-sum test (*P* < 0.05) and LDA score (log10) > 2. Differences of fecal metabolites between SP and HC group were determined by the two-tailed Student’s t-test (*P* < 0.05) and orthogonal partial least squares-discriminatory analysis (OPLS-DA) with Variable Importance in the Projection (VIP) > 1. Correlations between differential genera and clinical parameters, and correlations between differential genera and metabolites were evaluated by Spearman’s correlation analyses. Statistical analyses were performed with SPSS V.19.0 for Windows (SPSS, Chicago, Illinois, USA) and R V.3.5.1 (the R Project for Statistical Computing).

## Results

### Study population and clinical characteristics

**Fig. 1:**
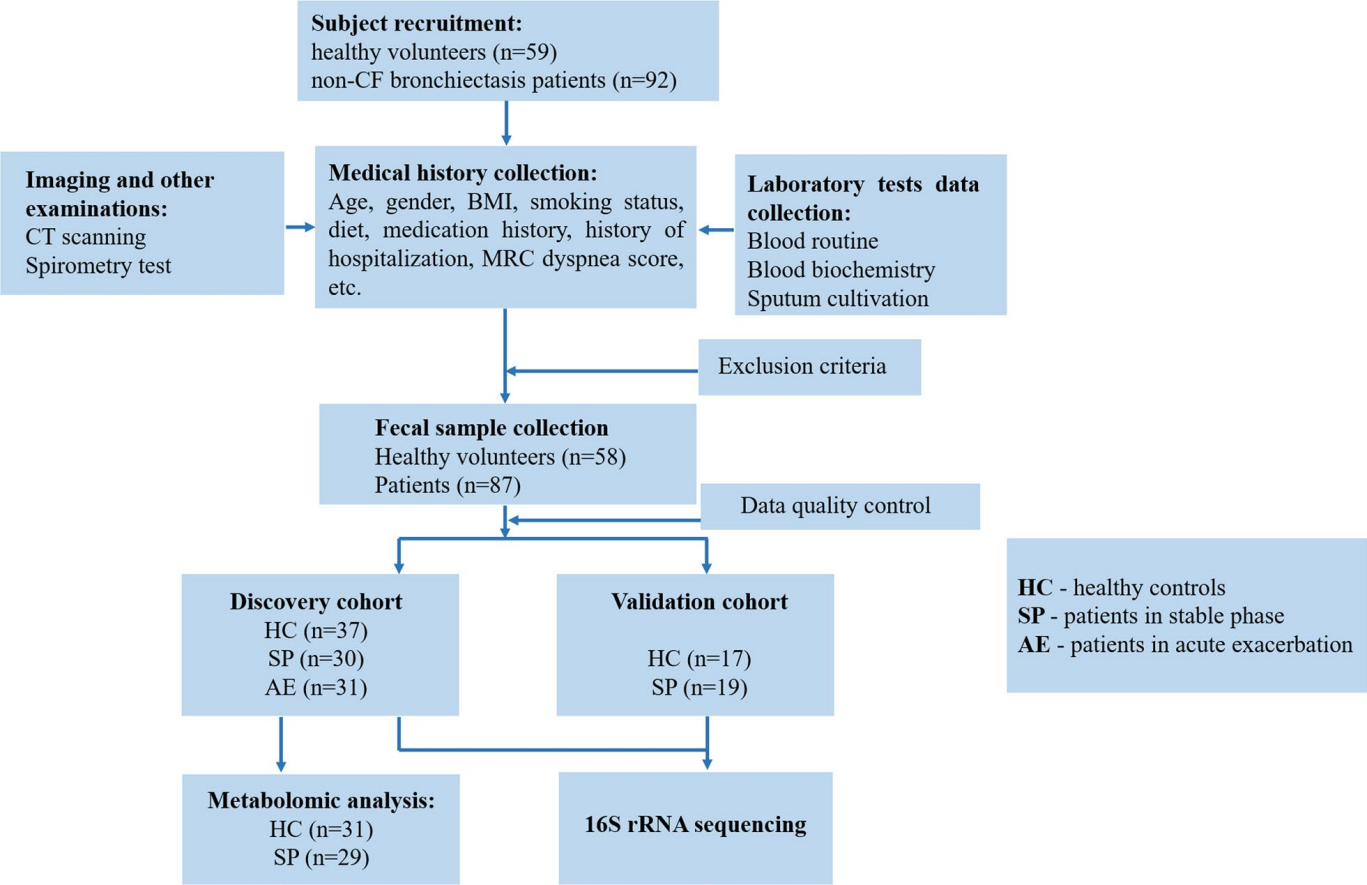
Flowchart. One hundred forty-five fecal samples were collected from Shanghai Pulmonary Hospital. Samples were randomly allocated to the discovery cohort and validation cohort. In the discovery cohort, 30, 31, and 37 fecal samples collected from the SP, AE, and HC groups, respectively, were assigned to 16 S rRNA gene sequencing. Thirty-one and 29 samples from HC and SP groups were assigned to LC-MS analysis

One hundred fifty-one subjects were recruited for this study. Eighty-seven patients and 58 healthy volunteers were included and randomly allocated to the discovery cohort and validation cohort (Fig. 1). In the discovery cohort, 30, 31, and 37 fecal samples were collected from SP, AE, and HC groups, respectively, and assigned to 16 S rRNA gene sequencing. Clinical characteristics, including age, gender, body mass index (BMI), and smoking status, were matched in three groups with the exception that the BMI of bronchiectasis patients (both SP and AE) was significantly lower than in the HC group (Table 1). All 31 patients in the AE group had received systemic antibiotics prescribed by a clinician in the previous month. There were no significant differences in *P. aeruginosa* infection, total bronchiectasis severity index (BSI) score, and St. George’s Respiratory Questionnaire (SGRQ) score (assigned at the time of sample collection) between the SP group and AE group. Pearson’s correlation analyses conducted among clinical characteristics showed significant correlations among forced expiratory volume in the first second (FEV1) % predicted values, BMI, BSI score, and SGRQ score (Additional file 1: Fig. S1). The participants’ baseline characteristics in the validation cohort were listed in Additional file 1: Table S2).

**Table 1:** Characteristics of the discovery cohort

	HC (n = 37)	SP (n = 30)	AE (n = 31)	*P* values	*P* values
				(HC vs. SP)	(SP vs. AE)
Age, years^†^	45.89 ± 10.37	50.57 ± 11.78	47.55 ± 13.84	0.092	0.363
Gender^‡^					
Female	25 (68%)	16 (53%)	15 (48%)	0.234	0.699
Male	12 (32%)	14 (47%)	16 (52%)		
BMI ^†^	24.24 ± 3.26	21.37 ± 2.38	21.25 ± 2.95	0	0.856
Smoking status^‡^					
Never smoker	34 (92%)	26 (87%)	27 (87%)	0.487	0.960
Ever smoker	3 (8%)	4 (13%)	4 (13%)		
Medication					
Oral / IV antibiotics^‡^	N/A	0	31		
FEV1% predicted^†^	N/A	73.26 ± 25.72	64.97 ± 21.08		0.173
*P. aeruginosa* infection^‡^	N/A				
Yes		17 (57%)	11 (35%)		0.097
No		13 (43%)	20 (65%)		
BSI score^†^	N/A	6.10 ± 2.85	7.19 ± 4.14		0.235
SGRQ score^†^	N/A	21.07 ± 8.02	25.21 ± 10.34		0.100

*N/A* not applicable; ^†^mean ± SD, ^‡^n (%)

### Decreased gut microbial diversity in bronchiectasis

**Fig. 2:**
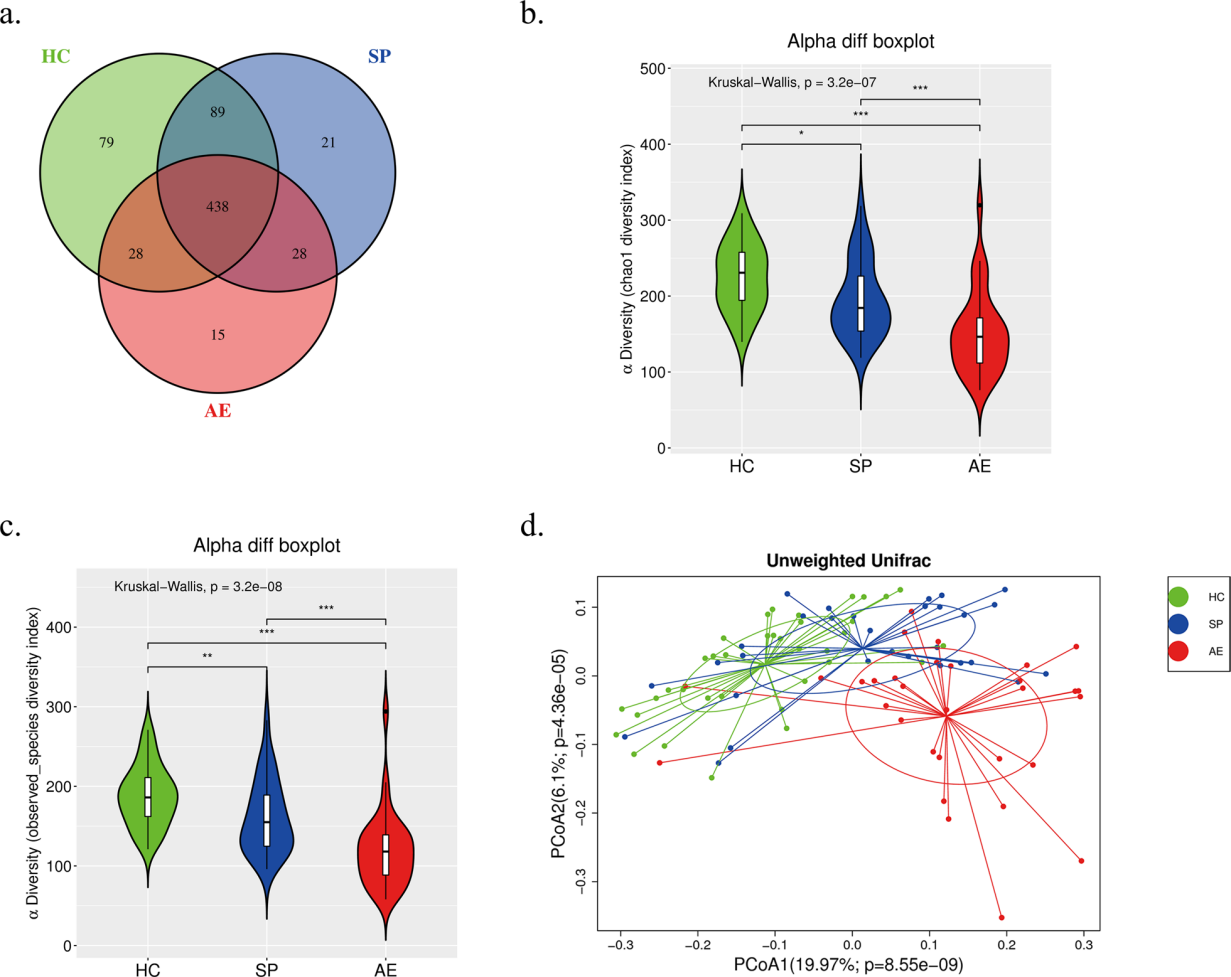
Decreased fecal microbial diversity in patients with bronchiectasis. **a** Venn diagram displaying the identified OTUs in three groups. α diversity estimated by Chao1 index (**b**) and observed species index (**c**). **d** β diversity calculated among the three groups using the unweighted UniFrac by PCoA. *P < 0.05, **P < 0.01, ***P < 0.001

A total number of 5,872,652 high-quality reads were obtained, with a median read count of 59,971 (50,372 to 64,962) per sample. The Venn diagram showed that 698 OTUs were obtained. Of these, 634, 576, and 509 OTUs were identified in the HC, SP, and AE groups, respectively, among which 79, 21, 15 OTUs were unique in the aforementioned three groups, respectively. When comparing HC with SP and HC with AE, 156 and 211 discriminatory OTUs, respectively, were separately identified (Fig. 2a). The sequences were aligned for analyses of α diversity and β diversity to evaluate the differences between microbial relative richness and diversity among the three groups. The gut microbiota α diversity, as estimated by the chao1 index and observed species index, was lower in bronchiectasis patients (both SP and AE group) than in the HC group (Fig. 2b and c). Principal coordinate analysis (PCoA) revealed a distinct distribution of fecal microbial communities among three groups (Fig. 2d). Results of multiple comparisons between the two groups were consistent with the above outcomes (Additional file 1: Fig. S2a, b, and c).

### Alterations of fecal microbial composition associated with bronchiectasis

**Fig. 3:**
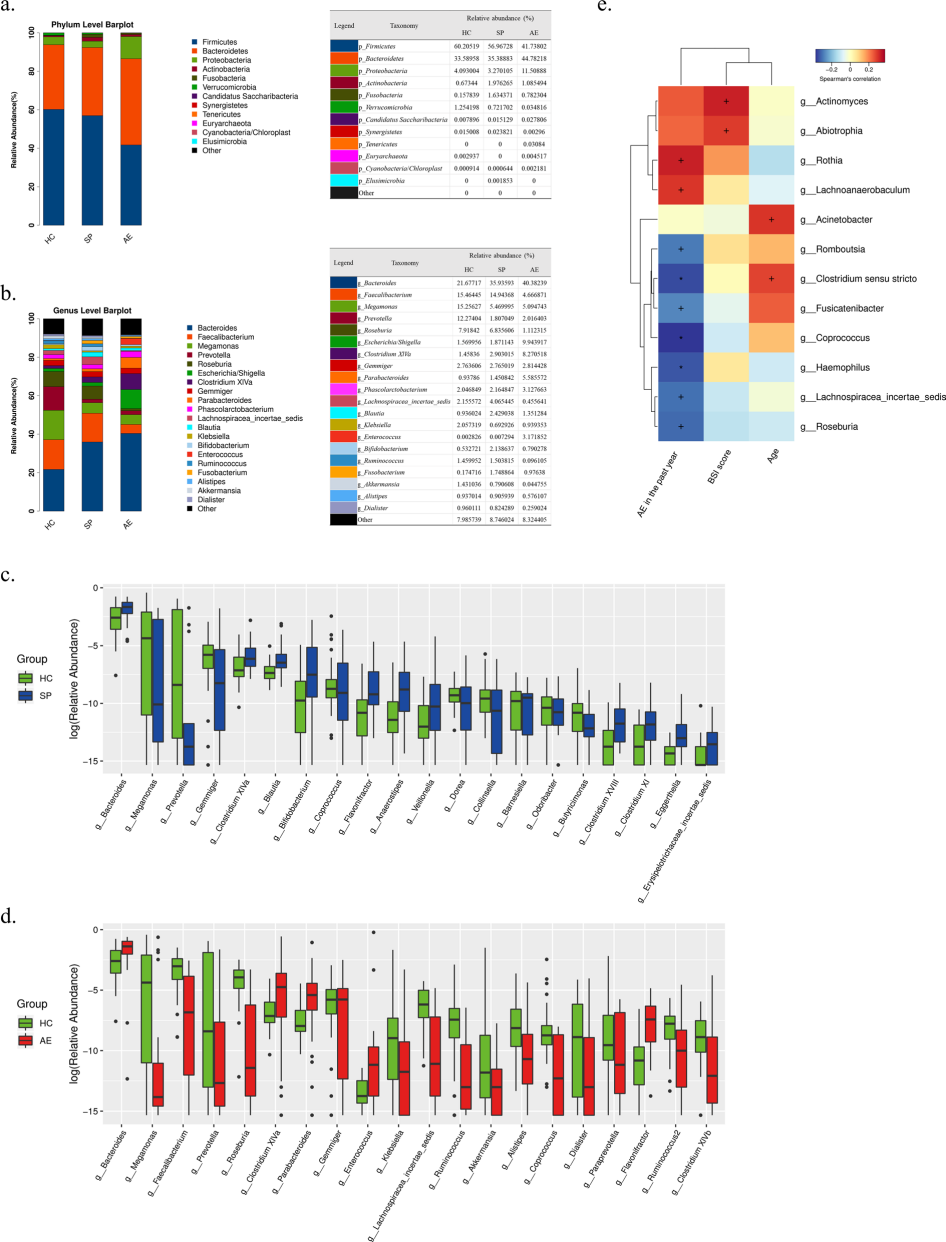
Distinct phylogenetic profiling of gut microbiota among the three groups. The relative proportions of dominant taxa at the phylum level (**a**) and genus level (**b**). The top 20 variant genera between HC and SP (**c**), HC and AE (**d**). **e** Correlations between variant genera and clinical parameters were evaluated by Spearman’s correlation analyses. ^+^*P* < 0.05, *P < 0.01

The relative proportions of dominant taxa at phylum and genus level were evaluated by microbial taxon assignment in three groups. Significant variability of gut microbiota in each group was observed (Fig. 3a and b). At the phylum level, *Firmicutes* was the most dominant phylum in HC and SP group, accounting for around 60.2% and 57.0% of the OTUs in the above two groups and its relative abundance was lower in both SP and AE group when comparing to HC group. *Bacteroidetes* accounted for around 44.8% of the OTUs and became the most predominant phylum in AE group. Data showed an elevated relative proportion of *Bacteroidetes* in SP and AE group compared with HC group, suggesting a decreased *Firmicutes* /*Bacteroidetes* (F/B) ratio in bronchiectasis patients. Compared to HC group, the relative abundance of Proteobacteria was enriched in AE group, although decreased in SP group samples. At the genus level, 23 and 50 altered genera were respectively identified in SP and AE group, among which, 15 and 32 genera were enriched in SP and AE group compared to those in HC group (Additional file 1: Tables S3 and S4). Here, we displayed the top 20 variant genera in box plots (Fig. 3c and d). Genera including *Bacteroides*, *Clostridium XI*, *Clostridium XIVa* and *Gemmiger* were found to be significantly more abundant, whereas genus such as *Prevotella*, *Coprococcus*, *Butyricimonas* and *Megamonas* showed a lower relative abundance in both SP and AE group that in HC group. Correlation between relative abundance of variant genera and clinical parameters including age, BSI score and times of acute exacerbations in the past year were further analyzed in bronchiectasis patients by Spearman’s correlation analyses represented by a heat map (Fig. 3e).

### Functional variations in the gut microbiota of bronchiectasis patients

We developed a phylogenetic investigation of communities by reconstructing unobserved states (PICRUSt) to predict the functional variations in the gut microbiota of bronchiectasis patients using 16 S rRNA gene sequencing data. LEfSe analysis was used to estimate the impact of each metabolic pathway (MetaCyc_pathway) on each component’s differential effect and determine the metabolic pathways significantly altered in each group. Of the 401 Meta_Cyc pathways tested, 97, 35, and 63 pathways were differentially enriched in AE, SP, and HC groups, respectively, with a screening condition at LDA score (log10) > 2 and *P* < 0.05 (Additional file 1: Fig. S3 and Table S5).

### Identification and validation of SP patients based on gut microbial genera

**Fig. 4:**
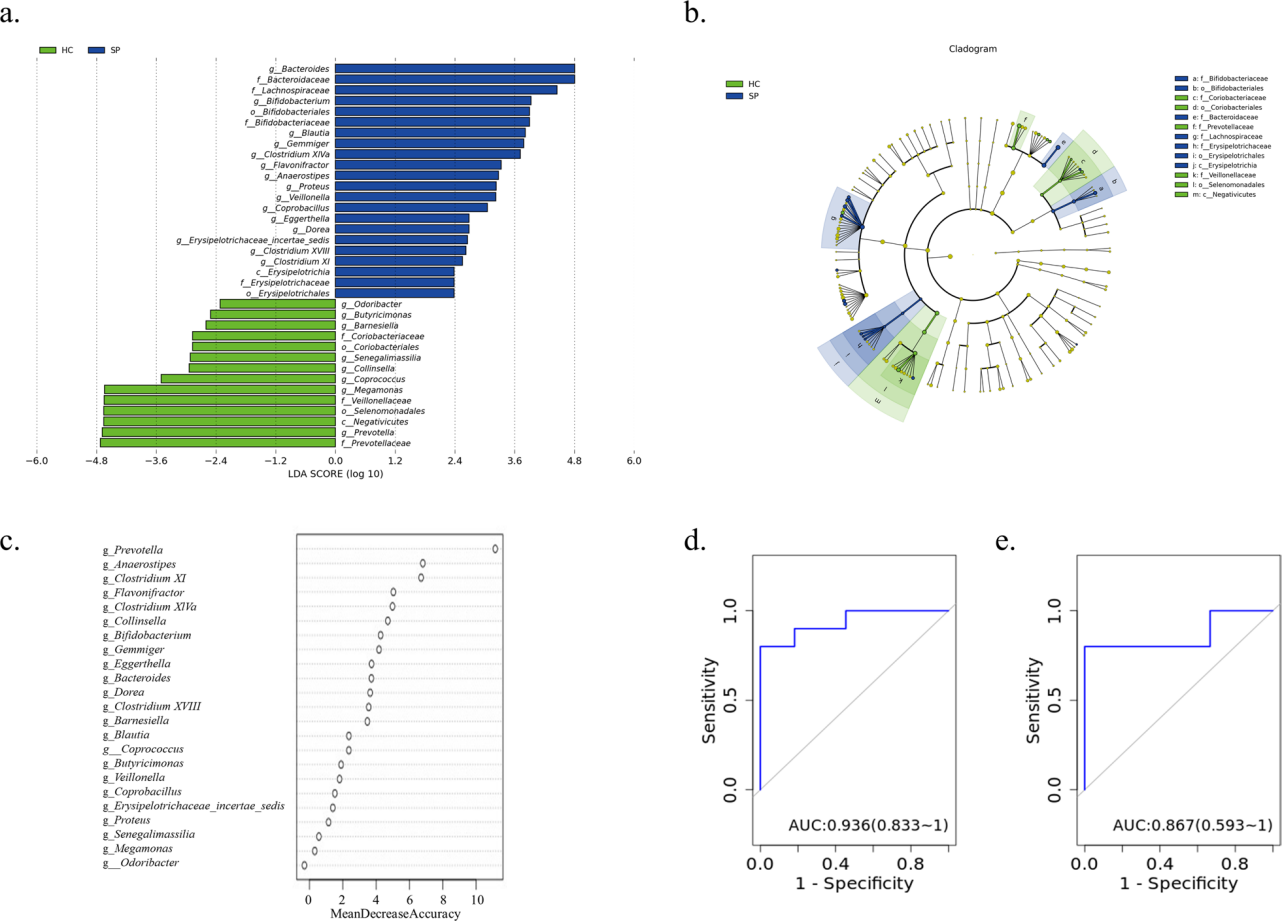
Identification and validation of bronchiectasis patients based on gut microbial genera. **a** LDA analyses identify differential taxa between HC and SP with LDA scores (log10) > 2. **b** Taxonomic cladogram indicating the phylogenetic distribution of gut microbiota in HC and SP. **c** MDA values of candidate genera are listed in descending order. The ROC curve was generated by the RF model using eight genera in the discovery cohort (**d**) and validation cohort (**e**)

Given that the patients in the AE group had a history of recent antibiotic use, which may have an enormous effect on the gut microbial composition and related metabolites, we only chose samples from the HC and SP groups for subsequent RF model establishment and metabolome analyses. LEfSe analyses identified all the taxa with the greatest differences in relative abundance between the SP and HC groups (Fig. 4a), and a Cladogram displayed the phylogenetic distribution of fecal microbiota associated with the above two groups (Fig. 4b). Twenty-three genera with LDA values (log10) larger than two were selected for the RF training referring to the aforementioned method (Additional file 1: Table S6). The top 10 genera with the highest MDA values were selected as candidates (Fig. 4c). Eventually, the combination of eight genera containing *Prevotella*, *Anaerostipes*, *Clostridium XI*, *Flavonifractor*, *Clostridium XlVa*, *Collinsella*, *Bifidobacterium*, and *Gemmiger* showed the best separating capacity with the AUC value of 0.936 (Fig. 4d). 16 S rRNA gene sequencing data collected from 17 HCs and 19 SPs in the validation cohort were used for an independent test to validate the discovery cohort’s findings. They achieved an ideal discriminative power with an AUC of 0.867 (Fig. 4e), despite the value being lower than that in the discovery cohort.

### Shifted fecal metabolome of bronchiectasis patients

**Fig. 5:**
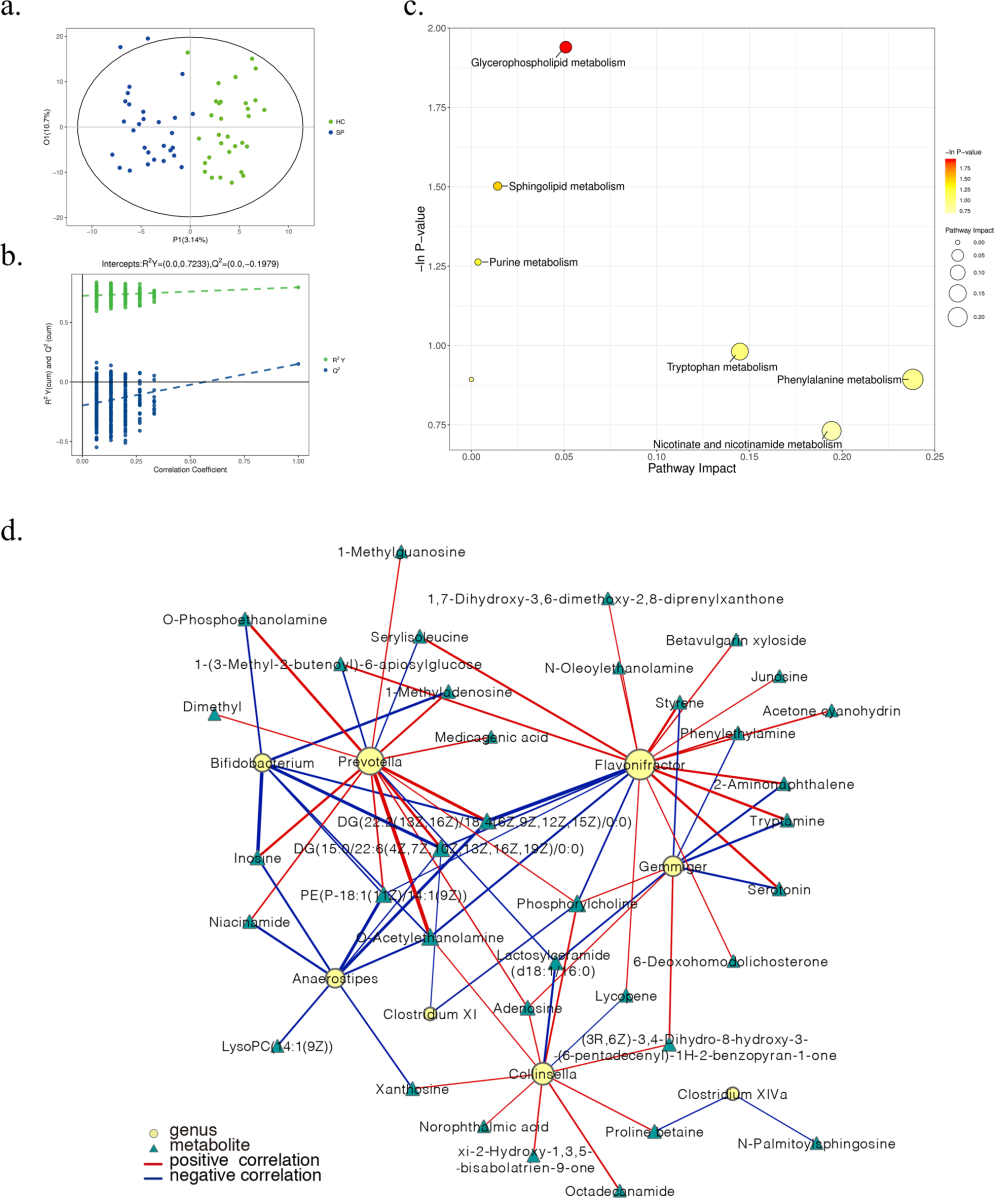
Differential fecal metabolomic features in HC and SP groups. **a** OPLS-DA score plot. **b** OPLS-DA validation plot intercepts: R2Y = (0.0, 0.7233), Q2 = (0.0, – 0.1979). **c** Bubble plot displaying the significantly enriched pathways. **d** Correlation network of the 8 genera and metabolites with *P* < 0.01. The size of each node is proportional to the quantity of related metabolites and the thickness of the line is proportional to the correlation coefficient. Lines between nodes and triangles indicate positive correlations (red) or negative correlations (blue)

Considering that the bronchiectasis patients possessed a perturbed gut microbiota potentially associated with the disease, we hypothesized that gut metabolism might be partially affected by gut microbiota in the bronchiectasis patients. Therefore, we conducted metabolome analyses of fecal samples by a nontargeted LC-MS-based metabolomics approach. A total of 835 metabolites were identified in both HC and SP groups (Additional file 1: Table S7). The OPLS-DA score plot showed clear discrimination between SP patients and HCs (Fig. 5a), and the negative intercept of the Q^2^ regression line in the permutation test indicated the validity of the OPLS-DA model (Fig. 5b). Forty-four differential metabolites (VIP > 1 and *P* < 0.05) were identified in the SPs. Metabolites such as adenosine, inosine, xanthosine, and phosphorylcholine were largely reduced, whereas metabolites like lactosylceramide, tryptamine, serotonin showed a significantly higher level in SPs (Additional file 1: Fig. S4). The significantly enriched metabolic pathways were displayed in the bubble plot to summarize the 44 variant metabolites (Fig. 5c). Glycerophospholipid metabolism, sphingolipid metabolism, purine metabolism, and tryptophan metabolism were the main pathways that responded to the SP group. Then, Spearman’s correlation analyses were conducted to clarify the correlations between the 23 differential genera and 44 metabolites, and the results revealed both positive and negative correlations for several genera-metabolites pairs in the SP group. For instance, some SP-reduced genera (*Prevotella*, *Gemmiger*, and *Butyricimonas*) were positively correlated with nucleosides, including adenosine, xanthosine, and inosine, which may be involved in purine metabolism (Additional file 1: Fig. S5). Furthermore, correlation networks were applied using the software Cytoscape to illuminate the associations between the 8 genera screened out by RF training model and the differential metabolites (Fig. 5d). In fact, to investigate whether fecal components of bronchiectasis patients can directly affect lung immunity, sterile fecal water (SFW) was extracted from fecal samples of clinical subjects, and MH-S cells (a continuous cell line of murine alveolar macrophages) were cultured with SFW (see Supplementary Methods). By using an enzyme-linked immunosorbent assay (ELISA) kit, we observed an elevated level of interleukin-6 (IL-6) in the SP group’s supernatant compared with the HC group (Additional file 1: Fig. S6).

## Discussion

According to previous literature reading and data collection, it is generally accepted that there were many factors affecting the composition and structure of intestinal microbes, including eating habits, age, gender, smoking status, taking antibiotics, probiotics, prebiotics and other supplements [26]. In this study, clinical characteristics, including age, gender, and smoking status, were matched in healthy control group and bronchiectasis group. Our baseline information indicated that compared with subjects without bronchiectasis, subjects with bronchiectasis (both SP and AE) have lower BMI. Though the cause of being underweight remains unclear, it is thought to be associated with decreased quality of life and more pulmonary comorbidities. Likewise, correlations between clinical parameters demonstrated that higher bronchiectasis severe index was positively correlated with poorer quality of life, and lower BMI is possibly related to decreased lung function and poorer life quality and prognosis in patients with bronchiectasis, which are consistent with the conclusions of earlier published articles [27, 28].

Multiple studies have uncovered the gut microbiota’s role in regulating the pulmonary immune microenvironment and maintaining healthy lungs, which is probably mediated by soluble bacterial components, metabolites, or innate lymphoid cells in the gut [29, 30]. The protective role of dietary fiber and probiotics in cohort studies of COPD and animal models of respiratory infection has been revealed [31–33], indicating that gut microbe interventions show great potential in treating respiratory disease.

Although no direct evidence has shown the correlation between the gut microbiota and bronchiectasis development, Butland has reported the development of progressive bronchiectasis within one year of proctocolectomy back to the early 1980s [34]. Subsequently, a literature review also indicated that the most common clinical manifestation of pulmonary involvement in patients with inflammatory bowel disease (IBD) was bronchiectasis [35]. In our preliminary experiments, sterile fecal water was extracted from fecal samples of bronchiectasis patients, and HCs referring to the protocol of Shenoy [36] and were co-cultured with MH-S cells. Higher level of interleukin-6 (IL-6) in the SP group’s supernatant (Additional file 1: Fig. S6) were detected. With the findings above, we hypothesized that there might be an altered gut microbiome and fecal metabolome in bronchiectasis patients.

Results of 16 S rRNA gene sequencing have uncovered a shifted gut microbial richness and diversity in bronchiectasis patients, characterized by a significant decline of α diversity as well as varied β diversity. We also observed a significant difference in the gut microbial diversity in patients with two clinical statuses (SP and AE). Although the antibiotics have a potent effect on gut microbial composition for patients in acute exacerbation (AE), there is a possibility that both antibiotic use and clinical exacerbation contribute to dysbiosis of the gut microbiome. Thus, in this study, screening out the microbial indicators to predict acute disease exacerbation is not applicable. Alterations of microbial composition at a different taxonomic level were also confirmed. Although an upregulated *Firmicutes* to *Bacteroidetes* ratio has been suggested as an indicator of several pathological conditions [14, 37, 38], our results suggested otherwise. Apart from acting as nutritional resources of the intestinal epithelium, short-chain fatty acids (SCFA) are widely believed to play various important roles in colonic epithelium maintenance, glucose homeostasis, lipid metabolism, and immune function [39–42]. In this study, the relative abundance of SCFA-producing bacteria, such as *Butyricimonas*, *Prevotella*, and *Coprococcus*, was significantly reduced in bronchiectasis patients. Thus, the decrease in SCFA-producing bacteria may promote intestinal mucosal destruction and alter the gut immune microenvironment, thereby contributing to the alterations in the distal immune in the lung. Using metabolomics, we identified several fecal metabolites and metabolic pathways altered in the bronchiectasis patients, including phospholipids, adenyl purines, amino acids, and peptides. Adenosine and adenosine-receptor signaling appear to have anti-inflammatory and barrier-protective effects in IBD, decreased extracellular adenosine, and elevated adenosine metabolic activity are thought to increase the inflammatory response in both the airway and intestine [43, 44]. Furthermore, bioactive sphingolipids are critical modulators of cell biological processes, including apoptosis, senescence, and inflammatory responses [45], and the distribution of variant sphingolipids is correlated with clinical status in IBD [46]. Sphingolipid metabolism is also associated with chronic inflammation in obesity, type 2 diabetes mellitus (T2DM), atherosclerosis [47, 48]. Sphingolipids, such as lactosylceramide, ceramides and sphingomyelins were thought to be involved in the regulation of pulmonary inflammatory response and play an important role in the process of P. aeruginosa infection in the lungs [49, 50]. The decreased fecal level of adenosine and variation in SPs’ sphingolipid metabolism may also suggest an increased inflammatory response in bronchiectasis. Tryptamine is a tryptophan-derived bacterial metabolite that is a common precursor molecule to many hormones and neurotransmitters [51, 52]. Serotonin is one of the most well-known tryptamines that has been recognized as important signaling molecule in the gut [53]. Previous studies have suggested that tryptamine down-regulated intestinal inflammatory reaction via modulating colonic mucus secretion in the mouse model of IBD [54, 55], and that serotonin signaling was altered in response to gastrointestinal disorders and inflammation [53]. Our data showed a higher fecal level of tryptamine and serotonin, as well as higher level of IL-6 in the SFW buffer in the SPs (Additional file 1: Figs. S4 and S6). Based on these findings, we assumed a proinflammatory state in the intestinal of bronchiectasis patients. It could be the cause or effect of the cross-talk between the intestinal microbiota and the lungs, termed the ‘gut-lung axis’ [13]. Although we have characterized an altered gut microbiome and fecal metabolome in bronchiectasis patients using a multi-omics-based approach, our study has limitations. It is only phenomenological research, and our approach to use the same cohort of subjects for both discovery and validation is not an external validation of results. Further well-designed experiments should be conducted to investigate the role of ‘gut-lung axis’ in bronchiectasis pathogenesis.

## Conclusion

To our knowledge, this is the first study to demonstrate that bronchiectasis patients exhibit distinct gut microbial and metabolic characteristics at both the clinical status of stable and AE. The gut microbiota has shown great potential as a noninvasive biomarker in bronchiectasis.

## Supplementary Information

**Additional file 1: Table S1.** Inclusionand exclusion criteria for subjects enrollment. **Table S2.** Characteristics of the validationcohort. **Table S3.** Relative abundance of 23 altered genera identified in SPgroup. **Table S4**. Relative abundance of 50 altered genera identified in AEgroup. **Table S5.** Significantly variant metabolicpathways identified in each group using LEfSe analyses. **TableS6.** LDA score values of 23 differential genera. **Table S7.** Eighthundred and thirty-five metabolites identified both from HC and SP group. **Fig. S1.** Correlationsbetween clinical parameters conducted by Pearson’s correlation analyses. **Fig.S2. **Differentiated fecal microbialdiversity in three groups. β diversity calculated between HC and SP (a), SP, and AE (b), HC and AE (c) calculated usingunweighted UniFrac by PCoA. **Fig. S3.** Statistical LEfSe analysis of differentialMetaCyc_pathway diagram at *P* < 0.05 and LDA scores (log10) > 2. **Fig. S4. **Forty-fourdifferential fecal metabolites in the HC and SP groups. **Fig. S5. **Correlations between the 23 differentialgenera and 44 metabolites by Spearman’s rank correlation coefficient analyses. +*P*<0.05,*P <0.01. **Fig. S6. **IL-6 levels in the supernatant of SFW-cultured MH-S cellsdetected by ELISA. +*P*<0.05.

## Data Availability

The datasets used and/or analyzed during the current study are available from the corresponding author on reasonable request.

## Abbreviations

16S rRNA: 16 S ribosomal RNA
AUC: Area under the curve
BMI: Body mass index
BSI: Bronchiectasis severity index
CF: Cystic fibrosis
COPD: Chronic obstructive pulmonary disease
F/B: *Firmicutes*/*Bacteroidetes*
FEV1: Forced expiratory volume in the 1st second
IBD: I nflammatory bowel disease
LC-MS: Lipid chromatography-mass spectrometry
LDA: Linear discriminant analysis
LEfSe: LDA effect size
OPLS-DA: Orthogonal partial least squares-discriminatory analysis
OTUs: O perational taxonomic units
PCoA: Principal coordinate analysis
PICRUSt: Phylogenetic investigation of communities by reconstruction of unobserved states
RDP: Ribosomal database project
RF: Random forest
ROC: Receiver operating characteristic
SCFA: Short-chain fatty acids
SFW: Sterile fecal water
SGRQ: St. George’s respiratory questionnaire
T2DM: Type 2 diabetes mellitus
UHPLC-MS: Ultra-high performance liquid chromatography-mass spectrometry
VIP: Variable importance in the projection
