# Supplementary material for: Altered fecal microbiome and metabolome in adult patients with non-cystic fibrosis bronchiectasis
Source: Respir Res. 2022 Nov 19;23:317. doi: 10.1186/s12931-022-02229-w (PMC9675243; doi:10.1186/s12931-022-02229-w)
Supplement: Supplementary file 1 — Additional file 1: Table S1. Inclusionand exclusion criteria for subjects enrollment. Table S2. Characteristics of the validationcohort. Table S3. Relative abundance of 23 altered genera identified in SPgroup. Table S4. Relative abundance of 50 altered genera identified in AEgroup. Table S5. Significantly variant metabolicpathways identified in each group using LEfSe analyses. TableS6. LDA score values of 23 differential genera. Table S7. Eighthundred and thirty-five metabolites identified both from HC and SP group. Fig. S1. Correlationsbetween clinical parameters conducted by Pearson’s correlation analyses. Fig.S2. Differentiated fecal microbialdiversity in three groups. β diversity calculated between HC and SP (a), SP, and AE (b), HC and AE (c) calculated usingunweighted UniFrac by PCoA. Fig. S3. Statistical LEfSe analysis of differentialMetaCyc_pathway diagram at P < 0.05 and LDA scores (log10) > 2. Fig. S4. Forty-fourdifferential fecal metabolites in the HC and SP groups. Fig. S5. Correlations between the 23 differentialgenera and 44 metabolites by Spearman’s rank correlation coefficient analyses. +P<0.05,*P <0.01. Fig. S6. IL-6 levels in the supernatant of SFW-cultured MH-S cellsdetected by ELISA. +P<0.05. [file 12931_2022_2229_MOESM1_ESM.docx]

| Table S1. Inclusion and exclusion criteria for subjects enrollment. |
| --- |
| **Inclusion criteria**: Each subject should meet all the following criteria to be enrolled in this study: |
| 1)Written informed consent |
| 2)Regular bowel movement: every 2 days - 3 times/ day |
| 3)The diagnose of bronchiectasis should refer to “BTS guideline 2010” |
| **Exclusion criteria**: Subjects meeting the following criteria should be excluded from this study: |
| 1)Diagnosis of chronic respiratory diseases including chronic obstructive pulmonary disease, tuberculosis, cystic fibrosis, asthma, allergic bronchopulmonary aspergillosis, lung cancer, or interstitial lung disease. |
| 2)pregnancy or lactation |
| 3)Active smoking or regular alcohol using within last 6 months |
| 4)HIV infection |
| 5)Previous abdominal or rectal surgery |
| 6)Diagnosis of chronic gastrointestinal disease (including gastroesophageal reflux disease), heart disease, diabetes, severe renal insufficiency (GFR < 30ml/min), alpha 1-antitrypsin deficiency, autoimmune disease or immunodeficiency. |
| 7)Regular use of the following types of medications (> 2 times per week) within last 2 months: inhaled or intravenous corticosteroids, inhaled bronchodilators, opium, loperamide antidiarrheal, systemic antihistaminic, metoclopramide, proton pump inhibitor |
| 8)Poor compliance or inability to cooperate as judged by the doctor |

| Table S2. Characteristics of the validation cohort. | | | |
| --- | --- | --- | --- |
|  | HC (n=17) | SP (n=19) | *P* values (HC vs SP) |
| Age, years^)^ | 53.94±12.20 | 55.53±10.20 | 0.812 |
| Gender^‡)^ |  |  |  |
| Female | 11（65%） | 13（68%） | 0.813 |
| Male | 6（35%） | 6（32%） |  |
| BMI^†)^ | 24.44±3.53 | 22.07±3.04 | 0.037 |
| Smoking status^‡)^ |  |  |  |
| Never smoker | 16（94%） | 17（89%） | 0.615 |
| Ever smoker | 1（6%） | 2（11%） |  |
|  |  | ^†^)mean±SD, ^‡)^n(%) | |

| Table S3. Relative abundance of 23 altered genera identified in SP group. | | | |
| --- | --- | --- | --- |
| Taxonomy | mean(HC) | mean(SP) | *P* values |
| g *Anaerostipes* | 0.00092 | 0.00462 | 0.00246 |
| g__*Bacteroides* | 0.19006 | 0.31308 | 0.00040 |
| g__*Barnesiella* | 0.00103 | 0.00035 | 0.00146 |
| g *Bifidobacterium* | 0.00402 | 0.01821 | 0.03096 |
| g__*Blautia* | 0.00800 | 0.02087 | 0.00112 |
| g__*Butyricimonas* | 0.00067 | 0.00021 | 0.01191 |
| g *Clostridium XI* | 0.00010 | 0.00053 | 0.00017 |
| g__*Clostridium XVIII* | 0.00014 | 0.00050 | 0.01332 |
| g__*Clostridium XlVa* | 0.01248 | 0.02460 | 0.00634 |
| g *Collinsella* | 0.00237 | 0.00118 | 0.00054 |
| g *Coprobacillus* | 0.00001 | 0.00002 | 0.01162 |
| g__*Coprococcus* | 0.01108 | 0.00621 | 0.00738 |
| g *Dorea* | 0.00206 | 0.00212 | 0.04989 |
| g__*Eggerthella* | 0.00003 | 0.00020 | 0.00098 |
| g__*Erysipelotrichaceae_incertae_sedis* | 0.00004 | 0.00010 | 0.00384 |
| g *Flavonifractor* | 0.00114 | 0.00570 | 0.00013 |
| g *Gemmiger* | 0.02430 | 0.02564 | 0.00336 |
| g__*Megamonas* | 0.14107 | 0.04492 | 0.00814 |
| g *Odoribacter* | 0.00081 | 0.00045 | 0.03701 |
| g__*Prevotella* | 0.11123 | 0.01604 | 0.00000 |
| g__*Proteus* | 0.00000 | 0.00001 | 0.02402 |
| g *Senegalimassilia* | 0.00007 | 0.00000 | 0.04630 |
| g__*Veillonella* | 0.00080 | 0.00427 | 0.03142 |

| Table S4. Relative abundance of 50 altered genera identified in AE group. | | | |
| --- | --- | --- | --- |
| Taxonomy | mean(HC) | mean(AE) | *P* values |
| g *Abiotrophia* | 0.00000 | 0.00025 | 0.00179 |
| g__*Acidaminococcus* | 0.00021 | 0.00000 | 0.00396 |
| g__*Acinetobacter* | 0.00000 | 0.00002 | 0.00171 |
| g *Actinomyces* | 0.00005 | 0.00148 | 0.00009 |
| g__*Akkermansia* | 0.01254 | 0.00035 | 0.01624 |
| g__*Alistipes* | 0.00808 | 0.00461 | 0.00021 |
| g *Allisonella* | 0.00072 | 0.00013 | 0.00055 |
| g__*Alloprevotella* | 0.00449 | 0.00010 | 0.04761 |
| g__*Asaccharobacter* | 0.00005 | 0.00003 | 0.03166 |
| g *Bacteroides* | 0.19006 | 0.36971 | 0.00004 |
| g__*Barnesiella* | 0.00103 | 0.00058 | 0.00065 |
| g__*Butyricimonas* | 0.00067 | 0.00034 | 0.00031 |
| g *Catabacter* | 0.00001 | 0.00000 | 0.01646 |
| g__*Clostridium XI* | 0.00010 | 0.00190 | 0.00956 |
| g__*Clostridium XlVa* | 0.01248 | 0.07645 | 0.00471 |
| g *Clostridium XlVb* | 0.00359 | 0.00315 | 0.00000 |
| g__*Clostridium sensu stricto* | 0.00052 | 0.00071 | 0.00084 |
| g__*Collinsella* | 0.00237 | 0.00137 | 0.00025 |
| g *Coprococcus* | 0.01108 | 0.00040 | 0.00000 |
| g__*Desulfovibrio* | 0.00067 | 0.00010 | 0.01424 |
| g__*Dialister* | 0.00862 | 0.00238 | 0.00150 |
| g *Dorea* | 0.00206 | 0.00074 | 0.00000 |
| g__*Eggerthella* | 0.00003 | 0.00046 | 0.00070 |
| g__*Enterococcus* | 0.00003 | 0.03129 | 0.00002 |
| g *Erysipelotrichaceae incertae sedis* | 0.00004 | 0.00036 | 0.00001 |
| g__*Faecalibacterium* | 0.13714 | 0.04150 | 0.00001 |
| g__*Flavonifractor* | 0.00114 | 0.00854 | 0.00005 |
| g *Fusicatenibacter* | 0.00264 | 0.00037 | 0.00000 |
| g__*Gemmiger* | 0.02430 | 0.02627 | 0.01515 |
| g__*Haemophilus* | 0.00083 | 0.00015 | 0.00004 |
| g *Holdemanella* | 0.00011 | 0.00003 | 0.02808 |
| g__*Klebsiella* | 0.01844 | 0.00806 | 0.00822 |
| g__*Lachnoanaerobaculum* | 0.00000 | 0.00001 | 0.04395 |
| g *Lachnospiracea incertae sedis* | 0.01886 | 0.00402 | 0.00000 |
| g__*Megamonas* | 0.14107 | 0.04560 | 0.00199 |
| g__*Odoribacter* | 0.00081 | 0.00023 | 0.00088 |
| g *Olsenella* | 0.00000 | 0.00001 | 0.02559 |
| g__*Oscillibacter* | 0.00295 | 0.00365 | 0.04703 |
| g__*Parabacteroides* | 0.00815 | 0.05175 | 0.00062 |
| g *Paraprevotella* | 0.00786 | 0.00122 | 0.00897 |
| g__*Prevotella* | 0.11123 | 0.01890 | 0.00656 |
| g__*Pyramidobacter* | 0.00010 | 0.00003 | 0.04124 |
| g *Rhodococcus* | 0.00000 | 0.00000 | 0.02638 |
| g__*Romboutsia* | 0.00136 | 0.00010 | 0.00000 |
| g__*Roseburia* | 0.06948 | 0.00999 | 0.00000 |
| g *Ruminococcus* | 0.01276 | 0.00076 | 0.00000 |
| g__*Ruminococcus2* | 0.00497 | 0.00386 | 0.00007 |
| g__*Senegalimassilia* | 0.00007 | 0.00000 | 0.04521 |
| g *Streptophyta* | 0.00001 | 0.00002 | 0.04061 |
| g__*Turicibacter* | 0.00006 | 0.00000 | 0.00123 |

Table S5. Significantly variant metabolic pathways identified in each group using LEfSe analyses.

| biomarker_name | enrich_group | LDA score | *P* values | MetaCyc_pathway |
| --- | --- | --- | --- | --- |
| FOLSYN-PWY | AE | 2.52238 | 1.06E-05 | superpathway of tetrahydrofolate biosynthesis and salvage |
| PWY-7199 | AE | 2.62767 | 1.33E-05 | pyrimidine deoxyribonucleosides salvage |
| PWY-7196 | AE | 2.51301 | 4.21E-03 | superpathway of pyrimidine ribonucleosides salvage |
| VALSYN-PWY | AE | 2.50375 | 3.83E-03 | L-valine biosynthesis |
| PWY-7222 | AE | 2.68156 | 6.58E-04 | guanosine deoxyribonucleotides de novo biosynthesis II |
| P461-PWY | AE | 2.62351 | 2.80E-03 | hexitol fermentation to lactate, formate, ethanol and acetate |
| PWY-7221 | AE | 2.32917 | 8.19E-03 | guanosine ribonucleotides de novo biosynthesis |
| CALVIN-PWY | AE | 2.31043 | 3.07E-02 | Calvin-Benson-Bassham cycle |
| PWY-6944 | AE | 2.03177 | 1.31E-02 | androstenedione degradation |
| GLUCONEO-PWY | AE | 2.26845 | 2.27E-03 | gluconeogenesis I |
| PWY-6609 | AE | 2.75949 | 3.12E-03 | adenine and adenosine salvage III |
| GLYCOGENSYNTH-PWY | AE | 2.56446 | 1.76E-02 | glycogen biosynthesis I (from ADP-D-Glucose) |
| PWY0-1296 | AE | 2.77038 | 1.31E-03 | purine ribonucleosides degradation |
| PWY-5686 | AE | 2.36541 | 5.82E-03 | UMP biosynthesis |
| PWY-7013 | AE | 2.74832 | 7.47E-03 | L-1,2-propanediol degradation |
| TRPSYN-PWY | AE | 2.50845 | 3.75E-03 | L-tryptophan biosynthesis |
| DENOVOPURINE2-PWY | AE | 2.47761 | 1.47E-03 | superpathway of purine nucleotides de novo biosynthesis II |
| PWY-6163 | AE | 2.49726 | 1.00E-05 | chorismate biosynthesis from 3-dehydroquinate |
| ARO-PWY | AE | 2.47410 | 1.32E-05 | chorismate biosynthesis I |
| NONOXIPENT-PWY | AE | 2.62463 | 1.99E-05 | pentose phosphate pathway (non-oxidative branch) |
| THRESYN-PWY | AE | 2.38571 | 9.80E-03 | superpathway of L-threonine biosynthesis |
| LACTOSECAT-PWY | AE | 2.98872 | 7.05E-09 | lactose and galactose degradation I |
| PWY-5022 | AE | 2.56724 | 2.20E-03 | 4-aminobutanoate degradation V |
| PWY-5419 | AE | 2.23130 | 1.65E-03 | catechol degradation to 2-oxopent-4-enoate II |
| PWY-7229 | AE | 2.54452 | 2.23E-03 | superpathway of adenosine nucleotides de novo biosynthesis I |
| PWY-7187 | AE | 2.55023 | 6.55E-06 | pyrimidine deoxyribonucleotides de novo biosynthesis II |
| PWY-7184 | AE | 2.56330 | 2.46E-04 | pyrimidine deoxyribonucleotides de novo biosynthesis I |
| PWY-6122 | AE | 2.49901 | 2.68E-03 | 5-aminoimidazole ribonucleotide biosynthesis II |
| PWY-6121 | AE | 2.41591 | 3.36E-03 | 5-aminoimidazole ribonucleotide biosynthesis I |
| PWY-6126 | AE | 2.52866 | 1.50E-03 | superpathway of adenosine nucleotides de novo biosynthesis II |
| GLYCOLYSIS | AE | 2.47671 | 3.07E-04 | glycolysis I (from glucose 6-phosphate) |
| PWY-7220 | AE | 2.68156 | 6.58E-04 | adenosine deoxyribonucleotides de novo biosynthesis II |
| PWY0-166 | AE | 2.60976 | 1.23E-06 | superpathway of pyrimidine deoxyribonucleotides de novo biosynthesis (E. coli) |
| PWY-7234 | AE | 2.53079 | 5.89E-04 | inosine-5'-phosphate biosynthesis III |
| P441-PWY | AE | 2.91949 | 3.74E-09 | superpathway of N-acetylneuraminate degradation |
| TEICHOICACID-PWY | AE | 2.59185 | 5.66E-04 | teichoic acid (poly-glycerol) biosynthesis |
| PWY-6386 | AE | 2.52490 | 1.21E-03 | UDP-N-acetylmuramoyl-pentapeptide biosynthesis II (lysine-containing) |
| PWY-6387 | AE | 2.49712 | 2.11E-03 | UDP-N-acetylmuramoyl-pentapeptide biosynthesis I (meso-diaminopimelate containing) |
| PWY-6385 | AE | 2.44514 | 2.59E-03 | peptidoglycan biosynthesis III (mycobacteria) |
| PWY-6317 | AE | 2.69117 | 1.17E-04 | galactose degradation I (Leloir pathway) |
| HOMOSER-METSYN-PWY | AE | 2.40070 | 2.60E-02 | L-methionine biosynthesis I |
| PWY0-1586 | AE | 2.84597 | 1.83E-04 | peptidoglycan maturation (meso-diaminopimelate containing) |
| PWY-5101 | AE | 2.52015 | 2.84E-03 | L-isoleucine biosynthesis II |
| PWY-5103 | AE | 2.32234 | 2.82E-02 | L-isoleucine biosynthesis III |
| SER-GLYSYN-PWY | AE | 2.60804 | 5.32E-05 | superpathway of L-serine and glycine biosynthesis I |
| PWY0-1319 | AE | 2.57154 | 4.42E-04 | CDP-diacylglycerol biosynthesis II |
| GLCMANNANAUT-PWY | AE | 2.93708 | 2.12E-09 | superpathway of N-acetylglucosamine, N-acetylmannosamine and N-acetylneuraminate degradation |
| PWY-7376 | AE | 2.33775 | 2.57E-03 | cob(II)yrinate a,c-diamide biosynthesis II (late cobalt incorporation) |
| PEPTIDOGLYCANSYN-PWY | AE | 2.48836 | 1.54E-03 | peptidoglycan biosynthesis I (meso-diaminopimelate containing) |
| ANAEROFRUCAT-PWY | AE | 2.41091 | 6.09E-03 | homolactic fermentation |
| HEXITOLDEGSUPER-PWY | AE | 2.82723 | 3.50E-06 | superpathway of hexitol degradation (bacteria) |
| PWY-6071 | AE | 2.22920 | 3.23E-02 | superpathway of phenylethylamine degradation |
| POLYISOPRENSYN-PWY | AE | 2.62338 | 2.31E-05 | polyisoprenoid biosynthesis (E. coli) |
| PWY-7208 | AE | 2.63211 | 3.06E-04 | superpathway of pyrimidine nucleobases salvage |
| PWY-7200 | AE | 2.46607 | 1.64E-03 | superpathway of pyrimidine deoxyribonucleoside salvage |
| PWY-922 | AE | 2.96878 | 1.92E-09 | mevalonate pathway I |
| PWY-5910 | AE | 2.98824 | 1.92E-09 | superpathway of geranylgeranyldiphosphate biosynthesis I (via mevalonate) |
| PWY-6185 | AE | 2.15281 | 2.40E-02 | 4-methylcatechol degradation (ortho cleavage) |
| PWY-6182 | AE | 2.20055 | 1.37E-02 | superpathway of salicylate degradation |
| PHOSLIPSYN-PWY | AE | 2.50993 | 1.07E-03 | superpathway of phospholipid biosynthesis I (bacteria) |
| ANAGLYCOLYSIS-PWY | AE | 2.31694 | 2.55E-02 | glycolysis III (from glucose) |
| PENTOSE-P-PWY | AE | 2.18073 | 1.18E-02 | pentose phosphate pathway |
| PWY-5420 | AE | 2.33306 | 2.42E-03 | catechol degradation II (meta-cleavage pathway) |
| UDPNAGSYN-PWY | AE | 2.38920 | 1.96E-03 | UDP-N-acetyl-D-glucosamine biosynthesis I |
| PWY-3001 | AE | 2.28847 | 2.41E-02 | superpathway of L-isoleucine biosynthesis I |
| PWY-6147 | AE | 2.40772 | 4.64E-02 | 6-hydroxymethyl-dihydropterin diphosphate biosynthesis I |
| PWY4FS-7 | AE | 2.47660 | 9.41E-04 | phosphatidylglycerol biosynthesis I (plastidic) |
| PWY-7663 | AE | 2.45974 | 2.73E-04 | gondoate biosynthesis (anaerobic) |
| ARGORNPROST-PWY | AE | 2.66679 | 1.60E-05 | arginine, ornithine and proline interconversion |
| MET-SAM-PWY | AE | 2.30472 | 2.59E-02 | superpathway of S-adenosyl-L-methionine biosynthesis |
| PWY-5667 | AE | 2.57154 | 4.42E-04 | CDP-diacylglycerol biosynthesis I |
| PWY-5181 | AE | 2.20548 | 4.45E-02 | toluene degradation III (aerobic) (via p-cresol) |
| PWY-5180 | AE | 2.54379 | 2.14E-03 | toluene degradation I (aerobic) (via o-cresol) |
| PWY-5182 | AE | 2.54379 | 2.14E-03 | toluene degradation II (aerobic) (via 4-methylcatechol) |
| PWY-5265 | AE | 2.68555 | 4.22E-03 | peptidoglycan biosynthesis II (staphylococci) |
| PWY-7111 | AE | 2.68205 | 2.38E-03 | pyruvate fermentation to isobutanol (engineered) |
| PWY-841 | AE | 2.27963 | 4.62E-02 | superpathway of purine nucleotides de novo biosynthesis I |
| HEMESYN2-PWY | AE | 2.80330 | 8.04E-05 | heme biosynthesis II (anaerobic) |
| COA-PWY | AE | 2.45110 | 8.09E-04 | coenzyme A biosynthesis I |
| PWY-7431 | AE | 2.33383 | 1.09E-02 | aromatic biogenic amine degradation (bacteria) |
| PWY-7219 | AE | 2.52332 | 1.07E-02 | adenosine ribonucleotides de novo biosynthesis |
| PWY-5484 | AE | 2.58354 | 6.01E-04 | glycolysis II (from fructose 6-phosphate) |
| PWY-6612 | AE | 2.54726 | 5.34E-05 | superpathway of tetrahydrofolate biosynthesis |
| PWY-621 | AE | 2.63931 | 3.05E-03 | sucrose degradation III (sucrose invertase) |
| PWY-5973 | AE | 2.33444 | 2.01E-03 | cis-vaccenate biosynthesis |
| PWY4FS-8 | AE | 2.47660 | 9.41E-04 | phosphatidylglycerol biosynthesis II (non-plastidic) |
| PWY-5430 | AE | 2.28740 | 2.00E-03 | meta cleavage pathway of aromatic compounds |
| COMPLETE-ARO-PWY | AE | 2.49714 | 2.15E-05 | superpathway of aromatic amino acid biosynthesis |
| f-1CMET2-PWY | AE | 2.52551 | 2.53E-04 | not found |
| ILEUSYN-PWY | AE | 2.50375 | 3.83E-03 | L-isoleucine biosynthesis I (from threonine) |
| PWY-6123 | AE | 2.44899 | 1.46E-03 | inosine-5'-phosphate biosynthesis I |
| PWY-6897 | AE | 2.49533 | 2.26E-02 | thiamin salvage II |
| PWY-6151 | AE | 2.43287 | 4.19E-02 | S-adenosyl-L-methionine cycle I |
| PWY-6471 | AE | 2.98436 | 1.01E-09 | peptidoglycan biosynthesis IV (Enterococcus faecium) |
| PWY-6277 | AE | 2.49901 | 2.68E-03 | superpathway of 5-aminoimidazole ribonucleotide biosynthesis |
| PWY-2941 | AE | 2.99812 | 4.34E-10 | L-lysine biosynthesis II |
| PWY-2942 | AE | 2.36020 | 2.29E-02 | L-lysine biosynthesis III |
| PWY-7198 | SP | 2.07503 | 8.00E-03 | pyrimidine deoxyribonucleotides de novo biosynthesis IV |
| P108-PWY | SP | 2.51876 | 2.45E-02 | pyruvate fermentation to propanoate I |
| PWY-6282 | SP | 2.57721 | 2.71E-02 | palmitoleate biosynthesis I (from (5Z)-dodec-5-enoate) |
| P23-PWY | SP | 2.70981 | 3.27E-04 | reductive TCA cycle I |
| ARG-POLYAMINE-SYN | SP | 2.38396 | 2.41E-03 | not found |
| PWY-6263 | SP | 2.02788 | 1.22E-02 | superpathway of menaquinol-8 biosynthesis II |
| PWY-5857 | SP | 2.54898 | 3.75E-03 | ubiquinol-10 biosynthesis (prokaryotic) |
| PWY-5856 | SP | 2.54898 | 3.75E-03 | ubiquinol-9 biosynthesis (prokaryotic) |
| PWY-5855 | SP | 2.54898 | 3.75E-03 | ubiquinol-7 biosynthesis (prokaryotic) |
| PWY-6519 | SP | 2.64386 | 9.87E-04 | 8-amino-7-oxononanoate biosynthesis I |
| GLYOXYLATE-BYPASS | SP | 2.51048 | 1.62E-03 | glyoxylate cycle |
| PWY0-1261 | SP | 2.58328 | 1.46E-04 | anhydromuropeptides recycling |
| PWY-6708 | SP | 2.54898 | 3.75E-03 | ubiquinol-8 biosynthesis (prokaryotic) |
| PYRIDNUCSAL-PWY | SP | 2.42818 | 5.99E-03 | NAD salvage pathway I |

| P105-PWY | SP | 2.71819 | 3.31E-05 | TCA cycle IV (2-oxoglutarate decarboxylase) |
| --- | --- | --- | --- | --- |
| BIOTIN-BIOSYNTHESIS-PWY | SP | 2.65225 | 8.84E-05 | biotin biosynthesis I |
| TCA-GLYOX-BYPASS | SP | 2.53854 | 1.62E-03 | superpathway of glyoxylate bypass and TCA |
| UBISYN-PWY | SP | 2.54658 | 3.37E-03 | superpathway of ubiquinol-8 biosynthesis (prokaryotic) |
| TCA | SP | 2.62195 | 1.72E-03 | TCA cycle I (prokaryotic) |
| GLYCOLYSIS-TCA-GLYOX-BYPASS | SP | 2.40183 | 1.12E-03 | superpathway of glycolysis, pyruvate dehydrogenase, TCA, and glyoxylate bypass |
| P42-PWY | SP | 2.69631 | 5.23E-03 | incomplete reductive TCA cycle |
| ALL-CHORISMATE-PWY | SP | 2.51379 | 6.25E-04 | superpathway of chorismate metabolism |
| PWY-6629 | SP | 2.53159 | 3.17E-08 | superpathway of L-tryptophan biosynthesis |
| PWY-6969 | SP | 2.67166 | 2.37E-04 | TCA cycle V (2-oxoglutarate:ferredoxin oxidoreductase) |
| PWY-6396 | SP | 2.44164 | 1.64E-02 | superpathway of 2,3-butanediol biosynthesis |
| PWY-5505 | SP | 2.53627 | 8.81E-03 | L-glutamate and L-glutamine biosynthesis |
| PWY-7211 | SP | 2.31022 | 2.92E-03 | superpathway of pyrimidine deoxyribonucleotides de novo biosynthesis |
| PWY-7210 | SP | 2.19877 | 7.97E-03 | pyrimidine deoxyribonucleotides biosynthesis from CTP |
| COLANSYN-PWY | SP | 2.42820 | 3.41E-03 | colanic acid building blocks biosynthesis |
| PYRIDOXSYN-PWY | SP | 2.51383 | 1.36E-03 | pyridoxal 5'-phosphate biosynthesis I |
| PWY-6895 | SP | 2.59194 | 1.45E-06 | superpathway of thiamin diphosphate biosynthesis II |
| PWY0-845 | SP | 2.37227 | 1.77E-02 | superpathway of pyridoxal 5'-phosphate biosynthesis and salvage |
| PWY0-1241 | SP | 2.61366 | 1.26E-04 | ADP-L-glycero-&beta;-D-manno-heptose biosynthesis |
| POLYAMSYN-PWY | SP | 2.26284 | 3.34E-02 | superpathway of polyamine biosynthesis I |
| FASYN-INITIAL-PWY | SP | 2.56745 | 1.52E-02 | superpathway of fatty acid biosynthesis initiation (E. coli) |
| ASPASN-PWY | HC | 2.75288 | 1.06E-05 | superpathway of L-aspartate and L-asparagine biosynthesis |
| PWY0-1338 | HC | 2.48483 | 9.70E-05 | polymyxin resistance |
| THISYN-PWY | HC | 2.80651 | 3.50E-08 | superpathway of thiamin diphosphate biosynthesis I |
| PWY-6608 | HC | 2.65016 | 5.27E-03 | guanosine nucleotides degradation III |
| PWY0-1297 | HC | 2.73075 | 3.14E-03 | superpathway of purine deoxyribonucleosides degradation |
| GALACTARDEG-PWY | HC | 2.47527 | 3.39E-04 | D-galactarate degradation I |
| PWY0-1479 | HC | 2.50731 | 2.09E-05 | tRNA processing |
| PWY-5896 | HC | 2.48818 | 7.30E-04 | superpathway of menaquinol-10 biosynthesis |
| PWY-6165 | HC | 2.04540 | 1.97E-08 | chorismate biosynthesis II (archaea) |
| ORNDEG-PWY | HC | 2.49479 | 3.27E-02 | superpathway of ornithine degradation |
| PWY-6467 | HC | 2.75387 | 9.06E-05 | Kdo transfer to lipid IVA III (Chlamydia) |
| PWY-5850 | HC | 2.48818 | 7.30E-04 | superpathway of menaquinol-6 biosynthesis I |
| PWY-6749 | HC | 2.74756 | 6.88E-04 | CMP-legionaminate biosynthesis I |
| AEROBACTINSYN-PWY | HC | 2.60970 | 2.13E-07 | aerobactin biosynthesis |
| PWY-5989 | HC | 2.64799 | 7.06E-03 | stearate biosynthesis II (bacteria and plants) |
| PWY-5097 | HC | 2.49905 | 5.94E-03 | L-lysine biosynthesis VI |
| PWYG-321 | HC | 2.61607 | 1.76E-02 | mycolate biosynthesis |
| GLUCOSE1PMETAB-PWY | HC | 2.57742 | 1.66E-03 | glucose and glucose-1-phosphate degradation |
| PWY-6891 | HC | 2.83414 | 1.12E-05 | thiazole biosynthesis II (Bacillus) |
| ECASYN-PWY | HC | 2.54179 | 1.54E-04 | enterobacterial common antigen biosynthesis |
| LPSSYN-PWY | HC | 2.09529 | 1.62E-06 | superpathway of lipopolysaccharide biosynthesis |
| PWY-5104 | HC | 3.06671 | 3.06E-05 | L-isoleucine biosynthesis IV |
| PWY-5100 | HC | 2.70660 | 2.29E-02 | pyruvate fermentation to acetate and lactate II |
| PWY-4984 | HC | 2.73477 | 3.04E-03 | urea cycle |
| PYRIDNUCSYN-PWY | HC | 2.67486 | 1.29E-03 | NAD biosynthesis I (from aspartate) |
| PWY0-1533 | HC | 2.63844 | 1.51E-05 | methylphosphonate degradation I |
| P101-PWY | HC | 2.08215 | 2.03E-04 | ectoine biosynthesis |
| GLUCARDEG-PWY | HC | 2.47964 | 2.84E-04 | D-glucarate degradation I |
| PWY-6353 | HC | 2.58947 | 2.10E-03 | purine nucleotides degradation II (aerobic) |
| NONMEVIPP-PWY | HC | 2.69839 | 8.40E-05 | methylerythritol phosphate pathway I |
| GLUCARGALACTSUPER-PWY | HC | 2.47527 | 3.39E-04 | superpathway of D-glucarate and D-galactarate degradation |
| ARGDEG-PWY | HC | 2.50437 | 2.40E-03 | superpathway of L-arginine, putrescine, and 4-aminobutanoate degradation |
| PWY-5862 | HC | 2.52366 | 2.41E-04 | superpathway of demethylmenaquinol-9 biosynthesis |
| PWY-5860 | HC | 2.52366 | 2.41E-04 | superpathway of demethylmenaquinol-6 biosynthesis I |
| PWY-5861 | HC | 2.31602 | 4.21E-02 | superpathway of demethylmenaquinol-8 biosynthesis |
| PWY-7328 | HC | 2.39106 | 6.98E-03 | superpathway of UDP-glucose-derived O-antigen building blocks biosynthesis |
| PWY-7242 | HC | 2.76455 | 7.99E-04 | D-fructuronate degradation |
| PWY0-1298 | HC | 2.66906 | 1.57E-03 | superpathway of pyrimidine deoxyribonucleosides degradation |
| KDO-NAGLIPASYN-PWY | HC | 2.64556 | 3.91E-07 | superpathway of (Kdo)2-lipid A biosynthesis |
| PWY-5347 | HC | 2.18425 | 4.70E-02 | superpathway of L-methionine biosynthesis (transsulfuration) |
| f-3-HYDROXYPHENYLACETATE-DEGRADATION-PW | HC | 2.56062 | 2.41E-04 | not found |
| PWY-5507 | HC | 2.04082 | 7.47E-05 | adenosylcobalamin biosynthesis I (early cobalt insertion) |
| PWY-7560 | HC | 2.69839 | 8.40E-05 | methylerythritol phosphate pathway II |
| PWY0-1415 | HC | 2.34198 | 1.24E-02 | superpathway of heme biosynthesis from uroporphyrinogen-III |
| PWY-181 | HC | 2.04889 | 2.27E-04 | photorespiration |
| P161-PWY | HC | 2.75846 | 7.29E-03 | acetylene degradation |
| KETOGLUCONMET-PWY | HC | 2.60871 | 2.19E-06 | ketogluconate metabolism |
| NAD-BIOSYNTHESIS-II | HC | 2.55418 | 2.61E-04 | NAD salvage pathway II |
| PWY-5304 | HC | 2.92797 | 6.92E-04 | superpathway of sulfur oxidation (Acidianus ambivalens) |
| PWY-5121 | HC | 2.52538 | 1.53E-02 | superpathway of geranylgeranyl diphosphate biosynthesis II (via MEP) |
| NAGLIPASYN-PWY | HC | 2.84071 | 1.00E-06 | lipid IVA biosynthesis |
| PWY-6892 | HC | 3.01516 | 6.55E-08 | thiazole biosynthesis I (E. coli) |
| PWY-7315 | HC | 2.59073 | 1.21E-04 | dTDP-N-acetylthomosamine biosynthesis |
| GALACTUROCAT-PWY | HC | 2.58673 | 1.65E-04 | D-galacturonate degradation I |
| GLUCUROCAT-PWY | HC | 2.63215 | 1.07E-03 | superpathway of &beta;-D-glucuronide and D-glucuronate degradation |
| DTDPRHAMSYN-PWY | HC | 2.71429 | 2.97E-02 | dTDP-L-rhamnose biosynthesis I |
| PWY-1269 | HC | 2.90180 | 1.13E-07 | CMP-3-deoxy-D-manno-octulosonate biosynthesis I |
| SALVADEHYPOX-PWY | HC | 2.72664 | 1.67E-03 | adenosine nucleotides degradation II |
| PPGPPMET-PWY | HC | 2.40416 | 2.24E-02 | ppGpp biosynthesis |
| ENTBACSYN-PWY | HC | 2.49922 | 1.11E-03 | enterobactin biosynthesis |
| ORNARGDEG-PWY | HC | 2.50437 | 2.40E-03 | superpathway of L-arginine and L-ornithine degradation |
| PWY-5845 | HC | 2.48818 | 7.30E-04 | superpathway of menaquinol-9 biosynthesis |
| PWY-722 | HC | 2.56572 | 2.65E-07 | nicotinate degradation I |

| Table S6. LDA score values of 23 differential genera. |  |  |  |
| --- | --- | --- | --- |
| biomarker_name | enrich_group | LDA score | *P* values |
| k Bacteria.p Bacteroidetes.c Bacteroidia.o Bacteroidales.f Bacteroidaceae.g Bacteroides | SP | 4.80633 | 5.25E-04 |
| k Bacteria.p Bacteroidetes.c Bacteroidia.o Bacteroidales.f Prevotellaceae.g Prevotella | HC | 4.68245 | 3.26E-06 |
| k Bacteria.p Firmicutes.c Negativicutes.o Selenomonadales.f Veillonellaceae.g Megamonas | HC | 4.63503 | 7.41E-03 |
| k Bacteria.p Actinobacteria.c Actinobacteria.o Bifidobacteriales.f Bifidobacteriaceae.g Bifidobacterium | SP | 3.93299 | 3.10E-02 |
| k Bacteria.p Firmicutes.c Clostridia.o Clostridiales.f Lachnospiraceae.g Blautia | SP | 3.81912 | 1.12E-03 |
| k Bacteria.p Firmicutes.c Clostridia.o Clostridiales.f Ruminococcaceae.g Gemmiger | SP | 3.78289 | 3.17E-03 |
| k Bacteria.p Firmicutes.c Clostridia.o Clostridiales.f Lachnospiraceae.g Clostridium XlVa | SP | 3.71682 | 6.22E-03 |
| k Bacteria.p Firmicutes.c Clostridia.o Clostridiales.f Lachnospiraceae.g Coprococcus | HC | 3.50059 | 7.11E-03 |
| k Bacteria.p Firmicutes.c Clostridia.o Clostridiales.f Ruminococcaceae.g Flavonifractor | SP | 3.33096 | 1.30E-04 |
| k Bacteria.p Firmicutes.c Clostridia.o Clostridiales.f Lachnospiraceae.g Anaerostipes | SP | 3.28059 | 2.47E-03 |
| k Bacteria.p Proteobacteria.c Gammaproteobacteria.o Enterobacteriales.f Enterobacteriaceae.g Proteus | SP | 3.22907 | 2.31E-02 |
| k Bacteria.p Firmicutes.c Negativicutes.o Selenomonadales.f Veillonellaceae.g Veillonella | SP | 3.21962 | 3.25E-02 |
| k Bacteria.p Firmicutes.c Erysipelotrichia.o Erysipelotrichales.f Erysipelotrichaceae.g Coprobacillus | SP | 3.05239 | 1.13E-02 |
| k Bacteria.p Actinobacteria.c Actinobacteria.o Coriobacteriales.f Coriobacteriaceae.g Collinsella | HC | 2.94211 | 5.28E-04 |
| k Bacteria.p Actinobacteria.c Actinobacteria.o Coriobacteriales.f Coriobacteriaceae.g Senegalimassilia | HC | 2.91492 | 4.51E-02 |
| k Bacteria.p Actinobacteria.c Actinobacteria.o Coriobacteriales.f Coriobacteriaceae.g Eggerthella | SP | 2.68340 | 1.10E-03 |
| k Bacteria.p Firmicutes.c Clostridia.o Clostridiales.f Lachnospiraceae.g Dorea | SP | 2.68212 | 4.92E-02 |
| k Bacteria.p Firmicutes.c Erysipelotrichia.o Erysipelotrichales.f Erysipelotrichaceae.g Erysipelotrichaceae incertae sedis | SP | 2.65270 | 4.61E-03 |
| k Bacteria.p Firmicutes.c Erysipelotrichia.o Erysipelotrichales.f Erysipelotrichaceae.g Clostridium XVIII | SP | 2.62182 | 1.40E-02 |
| k Bacteria.p Bacteroidetes.c Bacteroidia.o Bacteroidales.f Porphyromonadaceae.g Barnesiella | HC | 2.60195 | 1.43E-03 |
| k Bacteria.p Firmicutes.c Clostridia.o Clostridiales.f Peptostreptococcaceae.g Clostridium XI | SP | 2.55392 | 1.79E-04 |
| k Bacteria.p Bacteroidetes.c Bacteroidia.o Bacteroidales.f Porphyromonadaceae.g Butyricimonas | HC | 2.50456 | 1.13E-02 |
| k Bacteria.p Bacteroidetes.c Bacteroidia.o Bacteroidales.f Porphyromonadaceae.g Odoribacter | HC | 2.31232 | 3.59E-02 |

| Table S7. Eight hundred and thirty-five metabolites identified both from HC and SP group. | | | | | | | |
| --- | --- | --- | --- | --- | --- | --- | --- |
| Metabolites | Mean_HC | Mean_SP | *P* values | q values | FC | Log2FC | VIP_score |
| 10-Oxo-11-octadecen-13-olide | 0.13387 | 0.11304 | 0.44500 | 0.84346 | 1.18434 | 0.24409 | 0.05753 |
| 1,1'-[1,12-Dodecanediylbis(oxy)]bisbenzene | 7.20339 | 10.10850 | 0.42138 | 0.82772 | 0.71261 | -0.48882 | 0.53360 |
| 1-[1,4-Dihydro-4-nonyl-5-(1-oxodecyl)-3-pyridinyl]-1-dodecanone | 0.19470 | 0.20805 | 0.81611 | 0.94058 | 0.93581 | -0.09571 | 0.45232 |
| 11-beta-Hydroxyandrosterone-3-glucuronide | 0.38409 | 0.40498 | 0.80782 | 0.94058 | 0.94842 | -0.07640 | 0.60164 |
| 11-cis-Retinaldehyde | 0.14400 | 0.21390 | 0.41468 | 0.82772 | 0.67321 | -0.57087 | 1.02712 |
| 1-(1-Pyrrolidinyl)-2-butanone | 0.22849 | 0.31897 | 0.18437 | 0.80583 | 0.71633 | -0.48130 | 1.76317 |
| 1-(1-Pyrrolidinyl)-2-propanone | 2.69950 | 3.31468 | 0.63684 | 0.91307 | 0.81441 | -0.29618 | 0.80357 |
| 1,2,10-Trihydroxydihydro-trans-linalyl oxide 7-O-beta-D-glucopyranoside | 0.04901 | 0.02624 | 0.13786 | 0.80583 | 1.86788 | 0.90140 | 0.87384 |
| 1,2,3,4,5,6-Hexahydro-5-(1-hydroxyethylidene)-7H-cyclopenta[b]pyridin-7-one | 0.09214 | 0.14336 | 0.31871 | 0.82125 | 0.64268 | -0.63783 | 0.89282 |
| 1,2,3,4,5,6-Hexahydro-7H-cyclopenta[b]pyridin-7-one | 0.10297 | 0.26479 | 0.12919 | 0.80583 | 0.38888 | -1.36261 | 1.85424 |
| 1,2-Dihydro-1,1,6-trimethylnaphthalene | 0.23573 | 0.19977 | 0.17799 | 0.80583 | 1.18000 | 0.23879 | 0.73649 |
| [12]-Gingerol | 0.23437 | 0.30940 | 0.52519 | 0.87218 | 0.75751 | -0.40067 | 0.21831 |
| 12-HPETE | 0.49370 | 0.33164 | 0.40049 | 0.82398 | 1.48868 | 0.57404 | 0.42090 |
| 1,3-Diisopropylbenzene | 0.22391 | 0.22137 | 0.96774 | 0.99027 | 1.01148 | 0.01647 | 0.04207 |
| 1,3-Dimethyluracil | 7.53733 | 10.21296 | 0.23120 | 0.80583 | 0.73802 | -0.43828 | 1.62585 |
| 13-L-Hydroperoxylinoleic acid | 0.18052 | 0.15756 | 0.72293 | 0.92871 | 1.14576 | 0.19630 | 0.18666 |
| 1-(3-Methyl-2-butenoyl)-6-apiosylglucose | 0.14851 | 1.10115 | 0.00311 | 0.43316 | 0.13487 | -2.89038 | 2.04384 |
| 13-Oxo-9,11-tridecadienoic acid | 0.13922 | 0.15993 | 0.65059 | 0.91556 | 0.87051 | -0.20007 | 0.35485 |
| 13-Tetradecene-1,3-diyne-6,7-diol | 0.69125 | 0.40752 | 0.05534 | 0.75227 | 1.69625 | 0.76235 | 1.22090 |
| 1,4-Dideoxy-1,4-imino-D-ribitol | 1.37091 | 3.22949 | 0.06197 | 0.75227 | 0.42450 | -1.23617 | 1.69379 |
| 1-(4-Hydroxy-3-methoxyphenyl)-3-decanone | 0.60533 | 0.32709 | 0.05641 | 0.75227 | 1.85063 | 0.88802 | 1.19642 |
| 15-KETE | 0.12963 | 0.17060 | 0.44518 | 0.84346 | 0.75987 | -0.39618 | 0.74074 |
| 1,7-Dihydroxy-3,6-dimethoxy-2,8-diprenylxanthone | 0.07153 | 0.03146 | 0.04942 | 0.75227 | 2.27381 | 1.18511 | 0.46114 |
| 1-(9Z,12Z,15Z-octadecatrienoyl)-glycero-3-phosphate | 0.11646 | 0.19022 | 0.41736 | 0.82772 | 0.61226 | -0.70778 | 0.43028 |
| 1-Aminocyclopropanecarboxylic acid | 0.09085 | 0.09071 | 0.99333 | 0.99931 | 1.00149 | 0.00215 | 0.71039 |
| 1-Arachidonoylglycerophosphoinositol | 0.12018 | 0.11641 | 0.87784 | 0.95744 | 1.03241 | 0.04602 | 0.02132 |
| (1beta,2alpha,3alpha)-1,2,3,24-Tetrahydroxy-12-oleanen-28-oic acid | 0.13853 | 0.05777 | 0.32237 | 0.82125 | 2.39785 | 1.26174 | 1.10675 |
| 1-(beta-D-Ribofuranosyl)-1,4-dihydronicotinamide | 1.55763 | 0.88261 | 0.06874 | 0.75227 | 1.76480 | 0.81951 | 1.55701 |
| 1-Cyano-2-hydroxy-3-butene | 0.22384 | 0.56756 | 0.03731 | 0.75227 | 0.39439 | -1.34230 | 1.78461 |
| 1H-Indole-2,3-dione | 0.14675 | 0.20923 | 0.25876 | 0.80923 | 0.70136 | -0.51177 | 0.58991 |
| 1-Hydroxy-3,5-dimethoxy-2,4-diprenylxanthone | 0.00748 | 0.01067 | 0.65131 | 0.91556 | 0.70088 | -0.51276 | 0.62342 |
| 1-Isopropyl-2,3-dimethylcyclopentane | 0.04967 | 0.06429 | 0.78249 | 0.94011 | 0.77262 | -0.37216 | 0.31031 |
| 1-Isothiocyanato-7-(methylthio)heptane | 1.09938 | 0.73262 | 0.10071 | 0.80583 | 1.50062 | 0.58556 | 1.63086 |
| 1-Isothiocyanato-8-(methylthio)octane | 0.21522 | 0.16963 | 0.47411 | 0.84543 | 1.26876 | 0.34342 | 0.26349 |
| 1-Kestose | 0.11273 | 0.12691 | 0.69336 | 0.92441 | 0.88826 | -0.17095 | 0.71146 |
| 1-Methyladenine | 0.23262 | 0.29097 | 0.33107 | 0.82398 | 0.79947 | -0.32289 | 1.01022 |
| 1-Methyladenosine | 0.34316 | 0.15093 | 0.00191 | 0.43316 | 2.27367 | 1.18502 | 2.04730 |
| 1-Methylguanosine | 0.10533 | 0.04609 | 0.02164 | 0.75227 | 2.28546 | 1.19249 | 1.71177 |
| 1-Methylhistamine | 2.29829 | 0.62614 | 0.08010 | 0.75227 | 3.67054 | 1.87599 | 1.41714 |
| 1-Methylhistidine | 0.16671 | 0.14739 | 0.45298 | 0.84543 | 1.13108 | 0.17770 | 0.03533 |
| 1-Methylhypoxanthine | 1.16936 | 1.08247 | 0.75777 | 0.93126 | 1.08027 | 0.11139 | 0.04087 |
| 1-O-2'-Hydroxy-4'-methoxycinnamoyl-b-D-glucose | 0.05079 | 0.05568 | 0.74448 | 0.93126 | 0.91229 | -0.13244 | 0.38375 |
| 1-Octen-3-yl primeveroside | 0.02012 | 0.01783 | 0.70872 | 0.92626 | 1.12825 | 0.17408 | 0.33013 |
| 1-Palmitoylglycerophosphoinositol | 0.04222 | 0.18348 | 0.17520 | 0.80583 | 0.23012 | -2.11954 | 0.86233 |
| 1-Pyrroline | 0.12171 | 0.19297 | 0.01344 | 0.73899 | 0.63072 | -0.66493 | 2.49954 |
| 20-Carboxy-leukotriene B4 | 0.09591 | 0.01232 | 0.13963 | 0.80583 | 7.78459 | 2.96062 | 1.34852 |
| 20-Hydroxyeicosatetraenoic acid | 0.30228 | 0.13084 | 0.14115 | 0.80583 | 2.31036 | 1.20811 | 1.16493 |
| 2-(1-Pyrrolidinyl)-3-pentanone | 0.19158 | 0.30727 | 0.38504 | 0.82398 | 0.62351 | -0.68153 | 0.72435 |
| 22-Acetylpriverogenin B | 0.04109 | 0.03067 | 0.39764 | 0.82398 | 1.33955 | 0.42175 | 0.11051 |
| 2-(2-Furanyl)-3-methyl-2-butenal | 0.40936 | 0.35995 | 0.39354 | 0.82398 | 1.13728 | 0.18559 | 0.12168 |
| (2-{[3-(3,4-dihydroxyphenyl)prop-2-enoyl]oxy}ethyl)trimethylazanium | 0.19671 | 0.02829 | 0.36810 | 0.82398 | 6.95278 | 2.79759 | 0.40602 |
| 2-(3-Carboxy-3-aminopropyl)-L-histidine | 0.10174 | 0.11454 | 0.70619 | 0.92569 | 0.88819 | -0.17106 | 0.49146 |
| (+)-2,3-Dihydro-3-methyl-1H-pyrrole | 1.33034 | 1.27156 | 0.78955 | 0.94058 | 1.04623 | 0.06519 | 0.74829 |
| 2,3-Dihydro-5-(3-hydroxypropanoyl)-1H-pyrrolizine | 0.37117 | 0.28773 | 0.14091 | 0.80583 | 1.28998 | 0.36735 | 0.91495 |
| 2,3-Dihydro-5-(5-methyl-2-furanyl)-1H-pyrrolizine | 0.16353 | 0.70411 | 0.11663 | 0.80583 | 0.23225 | -2.10622 | 1.86642 |
| 24,25-Dihydroxyvitamin D | 0.54667 | 0.39252 | 0.13910 | 0.80583 | 1.39270 | 0.47788 | 0.83237 |
| 2,4-Dimethyloxazole | 0.14900 | 0.14681 | 0.90264 | 0.96628 | 1.01489 | 0.02133 | 0.45823 |
| (24R)-Ergost-4-ene-3,6-dione | 0.25573 | 0.20803 | 0.33393 | 0.82398 | 1.22934 | 0.29788 | 0.36956 |
| 25,27-Dihydro-4,7-didehydro-7-deoxyphysalin A | 0.04212 | 0.02742 | 0.20834 | 0.80583 | 1.53598 | 0.61916 | 0.50811 |
| 2,5-Diethylpyrazine | 0.15263 | 0.14387 | 0.79415 | 0.94058 | 1.06090 | 0.08529 | 0.18307 |
| 2,5-Dihydro-2,4-dimethyloxazole | 0.55401 | 0.71983 | 0.07031 | 0.75227 | 0.76965 | -0.37773 | 1.23873 |
| 2,5-Dimethyl-1H-pyrrole | 0.30489 | 0.26191 | 0.81915 | 0.94058 | 1.16410 | 0.21922 | 0.39467 |
| 25-Hydroxycholesterol | 0.55812 | 0.77984 | 0.22707 | 0.80583 | 0.71569 | -0.48259 | 1.27947 |
| 2,6-Dimethylaniline | 0.40473 | 1.30264 | 0.12853 | 0.80583 | 0.31070 | -1.68642 | 1.78947 |
| 2,6-Dimethylpyrazine | 0.09417 | 0.07746 | 0.55809 | 0.88968 | 1.21580 | 0.28190 | 0.42096 |
| 2,8-Di-O-methylellagic acid | 1.29344 | 0.90800 | 0.61651 | 0.91307 | 1.42449 | 0.51045 | 1.05313 |
| 28-Norcyclomusalenone | 0.37766 | 1.14489 | 0.37497 | 0.82398 | 0.32987 | -1.60003 | 0.97430 |
| 2-Acetyl-1,5,6,7-tetrahydro-6-hydroxy-7-(hydroxymethyl)-4H-azepine-4-one | 0.12127 | 0.10385 | 0.74689 | 0.93126 | 1.16781 | 0.22381 | 0.05818 |
| 2-acetyl-1-alkyl-sn-glycero-3-phosphocholine | 3.25389 | 3.87472 | 0.53762 | 0.87678 | 0.83977 | -0.25193 | 0.29444 |
| 2-Acetyl-5-methylpyridine | 1.44563 | 1.74655 | 0.39464 | 0.82398 | 0.82771 | -0.27281 | 1.46780 |
| 2-Aminoacetophenone | 0.38133 | 0.41315 | 0.69341 | 0.92441 | 0.92300 | -0.11560 | 0.45724 |
| 2-Aminoheptanedioic acid | 0.36855 | 0.54278 | 0.57384 | 0.89898 | 0.67901 | -0.55850 | 0.96842 |
| 2-Aminoisobutyric acid | 0.56328 | 0.74058 | 0.42022 | 0.82772 | 0.76060 | -0.39480 | 0.67542 |
| 2-Aminonaphthalene | 1.47001 | 3.51593 | 0.01826 | 0.75227 | 0.41810 | -1.25808 | 1.92254 |
| 2-Azetidinecarboxylic acid | 1.22896 | 1.09367 | 0.50215 | 0.86098 | 1.12371 | 0.16827 | 0.33143 |
| 2-Benzofurancarboxaldehyde | 0.17569 | 0.20371 | 0.53277 | 0.87495 | 0.86245 | -0.21349 | 1.29369 |
| 2'-Deoxymugineic acid | 0.05983 | 0.15659 | 0.05164 | 0.75227 | 0.38208 | -1.38804 | 1.73883 |
| 2-Dodecanone | 0.08125 | 0.08816 | 0.68626 | 0.92441 | 0.92162 | -0.11775 | 0.40418 |
| (2E)-N-(4-aminobutyl)-3-(4-hydroxy-3-methoxyphenyl)prop-2-enimidic acid | 0.05371 | 0.09571 | 0.34317 | 0.82398 | 0.56119 | -0.83344 | 1.37552 |
| (2E)-Piperamide-C5:1 | 0.10928 | 0.12931 | 0.79529 | 0.94058 | 0.84510 | -0.24281 | 0.48675 |
| 2-(Ethylamino)-4,5-dihydroxybenzamide | 0.24750 | 0.25017 | 0.95921 | 0.98827 | 0.98932 | -0.01549 | 0.52354 |
| 2-Ethyldihydro-3(2H)-thiophenone | 0.17941 | 0.17927 | 0.99683 | 0.99977 | 1.00077 | 0.00111 | 0.85378 |
| 2-Furancarboxaldehyde | 0.09688 | 0.11077 | 0.83284 | 0.94728 | 0.87458 | -0.19334 | 0.70483 |
| 2-Hexaprenyl-6-methoxyphenol | 0.11544 | 0.08797 | 0.16199 | 0.80583 | 1.31229 | 0.39208 | 0.38946 |
| 2-Hydroxy-6-pentadecylbenzoic acid | 0.46310 | 0.32823 | 0.28981 | 0.81978 | 1.41088 | 0.49659 | 0.35189 |

| 2-Hydroxypyridine | 0.29976 | 0.52365 | 0.18845 | 0.80583 | 0.57244 | -0.80482 | 1.61873 |
| --- | --- | --- | --- | --- | --- | --- | --- |
| 2-Isopropyl-3-methoxypyrazine | 0.35284 | 0.44760 | 0.29414 | 0.81978 | 0.78828 | -0.34321 | 1.44341 |
| 2-Isopropylphenyl methylcarbamate | 0.30435 | 0.89377 | 0.14144 | 0.80583 | 0.34053 | -1.55416 | 1.71229 |
| 2''-Methoxy--oleuropein | 0.02079 | 0.01846 | 0.80159 | 0.94058 | 1.12583 | 0.17098 | 0.48170 |
| 2-Methyl-1,3-cyclohexadiene | 0.71355 | 0.67557 | 0.64033 | 0.91307 | 1.05621 | 0.07890 | 0.15329 |
| 2-Methyl-1-methylthio-2-butene | 0.17553 | 0.31979 | 0.15418 | 0.80583 | 0.54889 | -0.86541 | 1.93153 |
| 2-Methyl-3-(2-methylpropyl)pyrazine | 0.12589 | 0.13434 | 0.77821 | 0.93997 | 0.93714 | -0.09367 | 0.65051 |
| 2-Methyl-3-(2-propenyl)pyrazine | 0.18901 | 0.20461 | 0.77295 | 0.93930 | 0.92374 | -0.11444 | 0.55289 |
| 2-Methylpyrrole | 0.06398 | 0.05870 | 0.75041 | 0.93126 | 1.08986 | 0.12415 | 0.15664 |
| 2-(Methylthio)propane | 0.09904 | 0.09802 | 0.96118 | 0.98827 | 1.01045 | 0.01500 | 0.46812 |
| 2(N)-Methyl-norsalsolinol | 2.16775 | 3.18362 | 0.32126 | 0.82125 | 0.68091 | -0.55447 | 1.29914 |
| 2-Phenylacetamide | 0.39062 | 0.39090 | 0.99597 | 0.99977 | 0.99928 | -0.00104 | 0.54928 |
| 2-Piperidinone | 65.49566 | 116.47042 | 0.04215 | 0.75227 | 0.56234 | -0.83049 | 1.69308 |
| 2-Propionylpyrrole | 0.31463 | 0.47522 | 0.39321 | 0.82398 | 0.66207 | -0.59494 | 1.29876 |
| 2-Pyrrolidineacetic acid | 0.33207 | 0.39228 | 0.62738 | 0.91307 | 0.84652 | -0.24038 | 0.84596 |
| 2-Pyrrolidinone | 0.89346 | 1.00679 | 0.64283 | 0.91307 | 0.88743 | -0.17229 | 0.50394 |
| (2R)-2-Hydroxy-2-methylbutanenitrile | 0.58767 | 0.75350 | 0.19744 | 0.80583 | 0.77992 | -0.35860 | 1.12848 |
| (2R,3R,4R)-2-Amino-4-hydroxy-3-methylpentanoic acid | 1.20541 | 0.84552 | 0.54544 | 0.88103 | 1.42564 | 0.51161 | 0.04514 |
| (2S,2'S)-Pyrosaccharopine | 0.08130 | 0.10775 | 0.59028 | 0.90771 | 0.75454 | -0.40634 | 0.84092 |
| (2S,4R)-4-(9H-Pyrido[3,4-b]indol-1-yl)-1,2,4-butanetriol | 0.09682 | 0.11963 | 0.25671 | 0.80583 | 0.80928 | -0.30529 | 1.47149 |
| (2S,4R,5S)-Muscarine | 0.20141 | 0.20966 | 0.94974 | 0.98827 | 0.96067 | -0.05789 | 0.58807 |
| (2S,4S)-Pinnatanine | 0.52178 | 1.06788 | 0.20385 | 0.80583 | 0.48862 | -1.03323 | 1.22121 |
| (2xi,20beta)-2,20-Dihydroxy-3-oxo-12-ursen-28-oic acid | 0.09924 | 0.08296 | 0.29243 | 0.81978 | 1.19629 | 0.25856 | 0.18191 |
| 3-(2-Furanylmethylene)pyrrolidine | 0.15184 | 0.30036 | 0.13852 | 0.80583 | 0.50552 | -0.98415 | 1.19484 |
| 3,4-Dihydro-5-(5-methyl-2-furanyl)-2H-pyrrole | 0.09311 | 0.34747 | 0.10486 | 0.80583 | 0.26798 | -1.89982 | 1.63153 |
| 3,4-Dihydro-5-propanoyl-2H-pyrrole | 0.20677 | 0.21601 | 0.83979 | 0.95251 | 0.95719 | -0.06313 | 0.19181 |
| 3,4-Dihydrocadalene | 0.30675 | 0.17105 | 0.04956 | 0.75227 | 1.79335 | 0.84265 | 1.23263 |
| 3,5-Dimethylphenyl methylcarbamate | 0.26441 | 0.31255 | 0.79375 | 0.94058 | 0.84600 | -0.24128 | 1.04340 |
| 3a,4b,7a-Trihydroxy-5b-cholanoic acid | 0.02855 | 0.02249 | 0.48630 | 0.84975 | 1.26924 | 0.34396 | 0.13062 |
| 3alpha,4,5,7alpha-Tetrahydro-5-hydroxy-1H-isoindole-1,3(2H)-dione | 1.44625 | 1.26865 | 0.37019 | 0.82398 | 1.14000 | 0.18903 | 0.36223 |
| 3-Amino-2-piperidone | 1.26212 | 0.99056 | 0.56044 | 0.89136 | 1.27414 | 0.34953 | 0.59967 |
| 3-Aminobutanoic acid | 0.92370 | 0.96717 | 0.84712 | 0.95251 | 0.95506 | -0.06634 | 0.25897 |
| 3-Aminocaproic acid | 0.20510 | 0.18406 | 0.32195 | 0.82125 | 1.11431 | 0.15615 | 0.17857 |
| (3b,4b,11b,14b)-11-Ethoxy-3,4-epoxy-14-hydroxy-12-cyathen-15-al 14-xyloside | 0.18933 | 0.07811 | 0.28514 | 0.81978 | 2.42385 | 1.27730 | 0.39215 |
| 3b,6b-Diangeloyloxy-7(11)-eremophilen-12,8b-olide | 0.09267 | 0.12795 | 0.72388 | 0.92871 | 0.72427 | -0.46540 | 0.36335 |
| 3b,8b-Dihydroxy-6b-(3-chloro-2-hydroxy-2-methylbutanoyloxy)-7(11)-eremophilen | 0.03227 | 0.01372 | 0.07775 | 0.75227 | 2.35134 | 1.23348 | 1.45794 |
| (3beta,22E,24R)-5,8-Epidioxy-23-methylergosta-6,22-dien-3-ol | 0.69580 | 0.97878 | 0.23003 | 0.80583 | 0.71089 | -0.49230 | 1.30525 |
| (3beta,5alpha,6beta,22E,24R)-23-Methylergosta-7,22-diene-3,5,6-triol | 1.09437 | 1.55422 | 0.12062 | 0.80583 | 0.70413 | -0.50609 | 1.56377 |
| (3beta,5alpha,6beta,9alpha,22E,24R)-23-Methylergosta-7,22-diene-3,5,6,9-tetrol | 0.29900 | 0.46910 | 0.15233 | 0.80583 | 0.63740 | -0.64974 | 1.45373 |
| 3-Carboxy-2,3,4,9-tetrahydro-1H-pyrido[3,4-b]indole-1-propanoic acid | 0.05026 | 0.07106 | 0.29535 | 0.81978 | 0.70723 | -0.49975 | 1.11458 |
| 3'-Deaminofusarochromanone | 0.11210 | 0.03945 | 0.08775 | 0.76203 | 2.84143 | 1.50662 | 1.32829 |
| 3-Dehydrosphinganine | 7.24305 | 5.78563 | 0.57734 | 0.89986 | 1.25190 | 0.32412 | 0.14984 |
| 3-Dehydroxycarnitine | 69.07464 | 94.09124 | 0.19899 | 0.80583 | 0.73412 | -0.44590 | 1.92245 |
| 3-Epidemissidine | 1.05381 | 0.80438 | 0.78710 | 0.94058 | 1.31009 | 0.38967 | 0.02846 |
| 3-Ethyl-2-methoxypyrazine | 1.91023 | 2.11971 | 0.69411 | 0.92441 | 0.90118 | -0.15012 | 0.75868 |
| 3-Ethylpyridine | 0.07913 | 0.09464 | 0.56487 | 0.89467 | 0.83619 | -0.25810 | 0.30108 |
| 3-Hydroxyanthranilic acid | 0.03293 | 0.03905 | 0.47822 | 0.84543 | 0.84318 | -0.24608 | 0.86192 |
| 3-Hydroxy-N-methylcoclaurine | 0.13398 | 0.01115 | 0.30253 | 0.82070 | 12.01358 | 3.58659 | 1.10813 |
| 3'-Hydroxy-T2-triol | 0.01740 | 0.00575 | 0.29194 | 0.81978 | 3.02860 | 1.59865 | 0.77662 |
| 3-Isovalidene-3alpha,4-dihydrophthalide | 0.39311 | 0.50340 | 0.25645 | 0.80583 | 0.78090 | -0.35679 | 1.40263 |
| 3-Methoxytyrosine | 0.05660 | 0.05621 | 0.97465 | 0.99296 | 1.00697 | 0.01002 | 0.58116 |
| 3-Methyl-3H-imidazo[4,5-f]quinoxalin-2-amine | 0.12884 | 0.16619 | 0.48980 | 0.84975 | 0.77527 | -0.36723 | 1.19321 |
| 3-Methyl-5-pentyl-2-furanundecanoic acid | 0.43609 | 0.76240 | 0.16913 | 0.80583 | 0.57200 | -0.80591 | 1.47994 |
| 3-Methyladenine | 0.39181 | 0.46937 | 0.27886 | 0.81613 | 0.83476 | -0.26057 | 0.65565 |
| 3-Methylcrotonylglycine | 0.61110 | 0.94762 | 0.56535 | 0.89467 | 0.64488 | -0.63289 | 0.98580 |
| 3-Methylcytosine | 0.54191 | 0.60319 | 0.48898 | 0.84975 | 0.89840 | -0.15456 | 0.63561 |
| 3-Methyldioxyindole | 0.42798 | 0.50129 | 0.47268 | 0.84543 | 0.85377 | -0.22809 | 1.16614 |
| 3-Methylguanine | 15.54510 | 17.22095 | 0.51803 | 0.86996 | 0.90269 | -0.14771 | 1.73368 |
| 3-Methylhistidine | 0.71295 | 0.52049 | 0.57993 | 0.89986 | 1.36977 | 0.45393 | 0.04834 |
| 3-Methyluridine | 0.11802 | 0.08099 | 0.13080 | 0.80583 | 1.45710 | 0.54310 | 0.62102 |
| 3'-N'-Acetylfusarochromanone | 0.02748 | 0.01335 | 0.30191 | 0.82070 | 2.05878 | 1.04179 | 0.96588 |
| 3-Phenylpropyl 2-methylpropanoate | 0.02013 | 0.01927 | 0.90510 | 0.96768 | 1.04433 | 0.06258 | 0.58841 |
| 3-Phenylpropyl isovalerate | 0.43351 | 0.27181 | 0.51136 | 0.86434 | 1.59491 | 0.67347 | 0.11570 |
| (3R, 6'Z)-3,4-Dihydro-8-hydroxy-3-(6-pentadecenyl)-1H-2-benzopyran-1-one | 3.52890 | 1.63484 | 0.02624 | 0.75227 | 2.15857 | 1.11007 | 1.21473 |
| (3S,3'R,5R,6R)-7',8'-Didehydro-3,6-epoxy-5,6-dihydro-beta,beta-carotene-3',5-diol | 0.59428 | 0.59045 | 0.97512 | 0.99296 | 1.00649 | 0.00934 | 0.70958 |
| 4',5,6,7,8-Pentahydroxy-3'-methoxyflavone | 0.03125 | 0.02931 | 0.72638 | 0.92871 | 1.06646 | 0.09283 | 0.06779 |
| 4,8,12,15-Octadecatetraenoic acid | 0.27571 | 0.23793 | 0.50759 | 0.86434 | 1.15878 | 0.21261 | 0.37187 |
| 4,8 Dimethylnonanoyl carnitine | 0.00564 | 0.03786 | 0.05985 | 0.75227 | 0.14887 | -2.74785 | 1.46641 |
| 4-Acetyl-2(3H)-benzoxazolone | 0.14815 | 0.10109 | 0.72740 | 0.92871 | 1.46546 | 0.55136 | 0.09764 |
| 4-Amino-2-methyl-1-naphthol | 0.23938 | 1.07961 | 0.08213 | 0.75227 | 0.22172 | -2.17316 | 1.96053 |
| 4-Amino-2-methylenebutanoic acid | 0.24493 | 0.23973 | 0.88291 | 0.95744 | 1.02169 | 0.03095 | 0.00964 |
| 4-Aminobutyraldehyde | 0.19869 | 0.16799 | 0.47891 | 0.84543 | 1.18274 | 0.24213 | 0.35566 |
| 4-Aminohippuric acid | 0.57837 | 0.24450 | 0.19246 | 0.80583 | 2.36557 | 1.24219 | 1.62758 |
| 4-Aminophenol | 0.90501 | 3.29575 | 0.11220 | 0.80583 | 0.27460 | -1.86461 | 1.82063 |
| 4-Coumaroyl-2-hydroxyputrescine | 0.09920 | 0.12162 | 0.53442 | 0.87495 | 0.81569 | -0.29391 | 0.71662 |
| 4-Guanidinobutanoic acid | 2.21130 | 2.84464 | 0.36173 | 0.82398 | 0.77736 | -0.36335 | 1.69930 |
| 4-Hydroxy-4-(methylnitrosoamino)-1-(3-pyridinyl)-1-butanone | 0.06468 | 0.11472 | 0.51041 | 0.86434 | 0.56386 | -0.82660 | 0.77356 |
| 4-Hydroxy-5-phenyltetrahydro-1,3-oxazin-2-one | 0.06542 | 0.08081 | 0.44692 | 0.84429 | 0.80954 | -0.30482 | 1.32879 |
| (4-Hydroxybenzoyl)choline | 0.06610 | 0.04224 | 0.14818 | 0.80583 | 1.56469 | 0.64588 | 1.03144 |
| 4-Hydroxybenzyl isothiocyanate 4''-acetylrhamnoside | 0.16451 | 0.08504 | 0.63568 | 0.91307 | 1.93451 | 0.95197 | 0.24170 |
| 4-Hydroxy-L-glutamic acid | 0.31487 | 0.33392 | 0.74736 | 0.93126 | 0.94297 | -0.08472 | 0.62221 |
| 4-Hydroxymandelonitrile | 1.28549 | 3.57901 | 0.34630 | 0.82398 | 0.35917 | -1.47724 | 0.28755 |
| 4-[(Hydroxymethyl)nitrosoamino]-1-(3-pyridinyl)-1-butanone | 0.88837 | 0.51992 | 0.19383 | 0.80583 | 1.70867 | 0.77288 | 0.96595 |
| 4-Methoxy-5-(3,7,11,15-tetramethyl-2,6,10,14-hexadecatetraenyl)-1,3-benzenediol | 0.15602 | 0.14206 | 0.68783 | 0.92441 | 1.09826 | 0.13522 | 0.08956 |
| 4-O-alpha-D-Galactopyranosylcalystegine B2 | 0.02763 | 0.06348 | 0.39565 | 0.82398 | 0.43528 | -1.19997 | 0.84279 |
| 4-Pyridoxic acid | 0.16956 | 0.13456 | 0.32390 | 0.82125 | 1.26014 | 0.33358 | 0.55417 |
| (4S,6S)-3,4,5,6-Tetrahydro-4-hydroxy-6-methyl-2H-pyran-2-one | 0.15268 | 0.18334 | 0.51012 | 0.86434 | 0.83279 | -0.26397 | 1.62986 |
| 4-Trimethylammoniobutanoic acid | 0.06404 | 0.06149 | 0.91258 | 0.97071 | 1.04143 | 0.05857 | 0.28964 |
| 5-(2-Furanyl)-1,2,3,4,5,6-hexahydro-7H-cyclopenta[b]pyridin-7-one | 0.17258 | 0.87031 | 0.12670 | 0.80583 | 0.19830 | -2.33424 | 1.77496 |

| 5-(2-Hydroxyethyl)-4-methylthiazole | 0.60688 | 1.42558 | 0.35944 | 0.82398 | 0.42571 | -1.23206 | 1.12962 |
| --- | --- | --- | --- | --- | --- | --- | --- |
| 5-(2-Hydroxyethyl)-4-methylthiazole acetate | 0.28527 | 0.47398 | 0.18757 | 0.80583 | 0.60185 | -0.73253 | 1.51710 |
| 5,6:8,9-Diepoxyergost-22-ene-3,7beta-diol | 0.03688 | 0.02827 | 0.41329 | 0.82772 | 1.30469 | 0.38371 | 0.41388 |
| 5,7,8-trihydroxy-2-phenyl-4H-chromen-4-one | 0.08396 | 0.89179 | 0.19391 | 0.80583 | 0.09415 | -3.40897 | 0.36390 |
| 5-Acetyl-2,4-dimethyloxazole | 0.47941 | 0.67760 | 0.26772 | 0.81355 | 0.70751 | -0.49918 | 1.40667 |
| 5-Acetylamino-6-formylamino-3-methyluracil | 0.08305 | 0.14538 | 0.04618 | 0.75227 | 0.57125 | -0.80781 | 1.96111 |
| 5alpha-Tomatidan-3-one | 0.12174 | 0.12830 | 0.92861 | 0.97779 | 0.94885 | -0.07575 | 0.53473 |
| 5-Aminoimidazole-4-carboxamide | 0.07079 | 2.20160 | 0.29966 | 0.82070 | 0.03215 | -4.95887 | 0.78624 |
| 5-Aminopentanamide | 0.29517 | 0.33641 | 0.59555 | 0.91097 | 0.87742 | -0.18867 | 0.67708 |
| 5-Aminopentanoic acid | 40.99873 | 39.62893 | 0.91003 | 0.97047 | 1.03457 | 0.04903 | 0.08800 |
| 5-Hexyltetrahydro-2-furanoctanoic acid | 0.90979 | 0.75776 | 0.30927 | 0.82125 | 1.20063 | 0.26379 | 0.80066 |
| 5-Hydroxyconiferyl alcohol | 0.20443 | 0.14306 | 0.14968 | 0.80583 | 1.42896 | 0.51496 | 0.90164 |
| 5-Hydroxy-L-tryptophan | 0.06355 | 0.07533 | 0.30272 | 0.82070 | 0.84367 | -0.24525 | 1.26781 |
| 5-Hydroxylysine | 0.25365 | 0.17000 | 0.63955 | 0.91307 | 1.49205 | 0.57730 | 0.34591 |
| 5-Methoxytryptamine | 0.07514 | 0.12713 | 0.31462 | 0.82125 | 0.59108 | -0.75858 | 1.64752 |
| 5-Methoxytryptophan | 6.36488 | 6.25771 | 0.94020 | 0.98503 | 1.01713 | 0.02450 | 0.79297 |
| 5-Methyl-2-propyloxazole | 0.23336 | 0.20885 | 0.78481 | 0.94020 | 1.11739 | 0.16013 | 0.35422 |
| 5-Methylcytosine | 0.32170 | 0.27686 | 0.42229 | 0.82772 | 1.16195 | 0.21655 | 0.14340 |
| 5-Methyldeoxycytidine | 0.10634 | 0.09136 | 0.56630 | 0.89467 | 1.16393 | 0.21900 | 0.07058 |
| 5'-Methylthioadenosine | 0.59122 | 1.23742 | 0.07119 | 0.75227 | 0.47778 | -1.06557 | 1.20085 |
| 5-Nitro-2-propoxyaniline | 0.24598 | 0.23857 | 0.88066 | 0.95744 | 1.03106 | 0.04413 | 0.69277 |
| 6-Acetyl-2,3-dihydro-2-(hydroxymethyl)-4(1H)-pyridinone | 0.22215 | 0.25825 | 0.61142 | 0.91307 | 0.86022 | -0.21721 | 0.74139 |
| 6-(alpha-D-Glucosaminyl)-1D-myo-inositol | 0.11936 | 0.10647 | 0.73971 | 0.93126 | 1.12105 | 0.16484 | 0.04953 |
| 6-Chloro-N-(1-methylethyl)-1,3,5-triazine-2,4-diamine | 2.18389 | 2.44391 | 0.66598 | 0.91627 | 0.89360 | -0.16229 | 0.87037 |
| 6-Deoxohomodolichosterone | 0.33759 | 0.55514 | 0.03700 | 0.75227 | 0.60811 | -0.71760 | 2.10055 |
| 6-Dimethylaminopurine | 0.16947 | 0.16878 | 0.98659 | 0.99734 | 1.00411 | 0.00591 | 0.20778 |
| 6-Epi-7-isocucurbic acid glucoside | 0.01934 | 0.01368 | 0.43592 | 0.83677 | 1.41414 | 0.49993 | 0.40981 |
| 6-Isothiocyanato-1-hexene | 0.48893 | 0.65614 | 0.20743 | 0.80583 | 0.74516 | -0.42438 | 1.84906 |
| 6-O-Methylcodeine | 1.42419 | 0.88520 | 0.58086 | 0.89986 | 1.60888 | 0.68606 | 0.04684 |
| 7-Ethoxy-4-methyl-2H-1-benzopyran-2-one | 0.03240 | 0.03394 | 0.90873 | 0.97032 | 0.95439 | -0.06735 | 0.32740 |
| 7-Hydroxy-2-methyl-4-oxo-4H-1-benzopyran-5-carboxylic acid 7-glucoside | 0.01689 | 0.01024 | 0.67886 | 0.92441 | 1.64912 | 0.72169 | 0.46638 |
| 7-Ketocholesterol | 0.14831 | 0.10270 | 0.13997 | 0.80583 | 1.44412 | 0.53019 | 0.71728 |
| 7-Methylinosine | 0.12363 | 0.06883 | 0.07374 | 0.75227 | 1.79606 | 0.84484 | 1.34648 |
| 8-Acetyl-T2 tetrol | 0.06768 | 0.04008 | 0.25512 | 0.80583 | 1.68853 | 0.75577 | 0.90478 |
| 8-HETE | 2.09037 | 2.58974 | 0.70359 | 0.92441 | 0.80717 | -0.30905 | 0.71481 |
| 8-Hydroxycarteolol | 0.07109 | 0.03142 | 0.46286 | 0.84543 | 2.26276 | 1.17808 | 0.81770 |
| 8-Hydroxycarvedilol | 0.00820 | 0.00797 | 0.91841 | 0.97442 | 1.02860 | 0.04069 | 0.64503 |
| 8-Ocimenyl acetate | 0.68992 | 0.44093 | 0.08311 | 0.75227 | 1.56467 | 0.64586 | 1.04616 |
| 9-HODE | 0.22443 | 0.24181 | 0.75833 | 0.93126 | 0.92815 | -0.10757 | 0.67869 |
| (9S,10E,12Z,15Z)-9-Hydroxy-10,12,15-octadecatrienoic acid | 0.52690 | 0.48749 | 0.63480 | 0.91307 | 1.08084 | 0.11215 | 0.26514 |
| (9Z,11R,12S,13S,15Z)-12,13-Epoxy-11-hydroxy-9,15-octadecadienoic acid | 0.04738 | 0.04587 | 0.89751 | 0.96333 | 1.03292 | 0.04672 | 0.19748 |
| Acetone cyanohydrin | 0.39062 | 1.12898 | 0.00297 | 0.43316 | 0.34599 | -1.53119 | 2.43883 |
| Acetylcholine | 0.37108 | 0.07697 | 0.36553 | 0.82398 | 4.82135 | 2.26944 | 0.25223 |
| Acetylcysteine | 0.03408 | 0.03067 | 0.81554 | 0.94058 | 1.11113 | 0.15203 | 0.17605 |
| Acetylhomoserine | 0.15416 | 0.29750 | 0.39416 | 0.82398 | 0.51817 | -0.94850 | 0.26551 |
| Acetyl tributyl citrate | 0.04154 | 0.05282 | 0.35549 | 0.82398 | 0.78645 | -0.34658 | 1.11178 |
| Acetylvalerenolic acid | 0.20128 | 0.11998 | 0.18822 | 0.80583 | 1.67757 | 0.74637 | 0.97183 |
| Adenine | 7.89301 | 10.50504 | 0.13514 | 0.80583 | 0.75135 | -0.41243 | 1.10175 |
| Adenosine | 7.27611 | 5.14733 | 0.03076 | 0.75227 | 1.41357 | 0.49934 | 1.43447 |
| Adrenochrome | 0.17268 | 0.17639 | 0.96570 | 0.98973 | 0.97895 | -0.03069 | 0.80000 |
| Aflatoxin B2 | 0.24718 | 0.04824 | 0.19327 | 0.80583 | 5.12442 | 2.35739 | 1.22564 |
| Alanyl-Arginine | 1.06549 | 0.97819 | 0.63822 | 0.91307 | 1.08925 | 0.12334 | 0.11906 |
| Alanyl-Leucine | 1.82415 | 1.67283 | 0.61756 | 0.91307 | 1.09045 | 0.12493 | 0.53549 |
| Alanyl-Proline | 0.33790 | 0.98031 | 0.09681 | 0.80036 | 0.34469 | -1.53662 | 1.81440 |
| Alanyl-Serine | 0.20677 | 0.31126 | 0.39090 | 0.82398 | 0.66430 | -0.59009 | 1.18444 |
| Alanyl-Valine | 1.34156 | 0.92746 | 0.05885 | 0.75227 | 1.44650 | 0.53257 | 1.49850 |
| Alloxanthin | 0.20262 | 0.17865 | 0.53337 | 0.87495 | 1.13413 | 0.18158 | 0.05360 |
| all-trans-Retinoic acid | 0.98134 | 0.72046 | 0.49011 | 0.84975 | 1.36210 | 0.44583 | 0.07017 |
| Allysine | 2.86280 | 2.80492 | 0.95367 | 0.98827 | 1.02064 | 0.02947 | 0.01444 |
| Aloin | 1.08491 | 0.11628 | 0.31487 | 0.82125 | 9.33021 | 3.22191 | 1.11224 |
| alpha-[3-[(Hydroxymethyl)nitrosoamino]propyl]-3-pyridinemethanol | 2.00411 | 0.60528 | 0.42882 | 0.83108 | 3.31104 | 1.72728 | 0.42234 |
| alpha-Acetolactate decarboxylase (enzyme preparation from bacillus subtilis recom | 0.03890 | 0.03671 | 0.81057 | 0.94058 | 1.05965 | 0.08359 | 0.01858 |
| alpha-Ionol O-[arabinosyl-(1->6)-glucoside] | 0.00579 | 0.00990 | 0.07988 | 0.75227 | 0.58415 | -0.77559 | 1.64058 |
| alpha-Micropteroxanthin B | 0.13469 | 0.19459 | 0.13574 | 0.80583 | 0.69218 | -0.53079 | 1.49911 |
| Aminoadipic acid | 0.18584 | 0.20942 | 0.55085 | 0.88284 | 0.88743 | -0.17229 | 0.87229 |
| Anabsinthin | 0.02440 | 0.02073 | 0.52149 | 0.86996 | 1.17717 | 0.23532 | 0.17645 |
| Anandamide | 0.05115 | 0.29713 | 0.26892 | 0.81355 | 0.17213 | -2.53841 | 1.18204 |
| Androsterone sulfate | 0.02479 | 0.26460 | 0.28155 | 0.81629 | 0.09371 | -3.41573 | 1.06801 |
| Aniline | 1.49762 | 4.92209 | 0.11976 | 0.80583 | 0.30427 | -1.71660 | 1.79423 |
| Anserine | 0.80992 | 0.22463 | 0.29446 | 0.81978 | 3.60559 | 1.85023 | 0.81435 |
| Antibiotic SB 202742 | 0.03652 | 0.03313 | 0.69743 | 0.92441 | 1.10232 | 0.14054 | 0.05458 |
| Arachidyl carnitine | 0.19246 | 0.23115 | 0.62858 | 0.91307 | 0.83264 | -0.26423 | 0.47933 |
| ar-Artemisene | 0.44836 | 0.51817 | 0.76902 | 0.93930 | 0.86527 | -0.20877 | 0.48347 |
| Arborinine | 0.03942 | 0.01083 | 0.21498 | 0.80583 | 3.64121 | 1.86442 | 0.96693 |
| Arginyl-Phenylalanine | 0.15443 | 0.15387 | 0.98382 | 0.99575 | 1.00362 | 0.00521 | 0.02842 |
| Arginyl-Proline | 0.02583 | 0.03496 | 0.42432 | 0.82977 | 0.73885 | -0.43665 | 1.08882 |
| Arginyl-Serine | 0.13394 | 0.10938 | 0.57055 | 0.89551 | 1.22457 | 0.29227 | 0.34827 |
| Arginyl-Valine | 0.47845 | 0.39660 | 0.40550 | 0.82398 | 1.20639 | 0.27070 | 0.79516 |
| AS 1-5 | 0.28622 | 0.55602 | 0.13819 | 0.80583 | 0.51477 | -0.95800 | 1.10502 |
| Asparaginyl-Hydroxyproline | 2.13790 | 2.67614 | 0.68384 | 0.92441 | 0.79887 | -0.32396 | 0.58697 |
| Asparaginyl-Proline | 0.03575 | 0.05631 | 0.52667 | 0.87256 | 0.63486 | -0.65549 | 0.35560 |
| Aspartame | 0.08437 | 0.08888 | 0.78055 | 0.93997 | 0.94932 | -0.07503 | 1.02012 |
| Aspartyl-Arginine | 0.09090 | 0.07396 | 0.36484 | 0.82398 | 1.22895 | 0.29743 | 0.12096 |
| Aspartyl-Isoleucine | 0.60371 | 0.66014 | 0.63032 | 0.91307 | 0.91451 | -0.12893 | 0.61724 |
| Aspartyl-Lysine | 0.06910 | 0.07357 | 0.75839 | 0.93126 | 0.93919 | -0.09051 | 0.71477 |
| Aspartylphenylalanine | 0.05224 | 0.05453 | 0.81702 | 0.94058 | 0.95805 | -0.06183 | 1.03627 |
| Aspartyl-Valine | 0.09987 | 0.08687 | 0.61833 | 0.91307 | 1.14973 | 0.20129 | 0.38431 |
| Asymmetric dimethylarginine | 1.38818 | 1.22602 | 0.65978 | 0.91627 | 1.13226 | 0.17921 | 0.24935 |
| Benzaldehyde | 0.21232 | 0.22060 | 0.85984 | 0.95530 | 0.96248 | -0.05517 | 0.58360 |

| Benzoic acid | 0.58014 | 0.64204 | 0.66011 | 0.91627 | 0.90360 | -0.14624 | 1.11130 |
| --- | --- | --- | --- | --- | --- | --- | --- |
| Benzoyl ecgonine | 0.04619 | 0.13542 | 0.25614 | 0.80583 | 0.34112 | -1.55165 | 1.51889 |
| Berberine | 2.60222 | 0.06048 | 0.27183 | 0.81355 | 43.02533 | 5.42711 | 1.01648 |
| beta-Alanine | 2.09925 | 2.76052 | 0.37985 | 0.82398 | 0.76045 | -0.39507 | 1.63994 |
| Beta-Carboline | 4.33510 | 4.18115 | 0.85250 | 0.95293 | 1.03682 | 0.05217 | 0.42738 |
| beta-Cryptoxanthin | 0.72849 | 0.79364 | 0.66255 | 0.91627 | 0.91792 | -0.12357 | 1.09289 |
| Betagarin | 0.14707 | 0.03121 | 0.30809 | 0.82125 | 4.71268 | 2.23655 | 1.07159 |
| Betalamic acid | 0.08278 | 0.07566 | 0.84536 | 0.95251 | 1.09409 | 0.12974 | 0.30676 |
| beta-Sitostenone | 0.09768 | 0.20675 | 0.35646 | 0.82398 | 0.47246 | -1.08174 | 1.05219 |
| beta-Thujaplicin | 2.41842 | 4.01027 | 0.24903 | 0.80583 | 0.60306 | -0.72963 | 1.33176 |
| Betavulgarin xyloside | 0.03347 | 0.07450 | 0.03338 | 0.75227 | 0.44925 | -1.15442 | 1.92980 |
| Bilirubin | 0.02983 | 0.04283 | 0.36377 | 0.82398 | 0.69648 | -0.52185 | 1.07619 |
| Biliverdin | 0.26224 | 0.20579 | 0.34278 | 0.82398 | 1.27429 | 0.34969 | 0.81833 |
| Biotripyrrin-b | 0.01391 | 0.01533 | 0.75591 | 0.93126 | 0.90749 | -0.14004 | 0.50302 |
| Brassica napus non-fluorescent chlorophyll catabolite 3 | 0.09210 | 0.06313 | 0.33528 | 0.82398 | 1.45889 | 0.54487 | 0.69573 |
| Bufotenine O-glucoside | 0.82374 | 0.69536 | 0.88122 | 0.95744 | 1.18462 | 0.24443 | 0.25380 |
| Butyl -3-hydroxybutyrate glucoside | 0.02986 | 0.02668 | 0.69876 | 0.92441 | 1.11893 | 0.16212 | 0.23388 |
| Cadabicine methyl ether | 0.25555 | 0.20123 | 0.55620 | 0.88968 | 1.26998 | 0.34480 | 0.35705 |
| Caffeine | 0.06940 | 0.05953 | 0.78125 | 0.93997 | 1.16591 | 0.22146 | 0.31669 |
| Calabaxanthone | 0.04589 | 0.02254 | 0.06284 | 0.75227 | 2.03559 | 1.02545 | 1.64481 |
| Calcitriol | 0.51782 | 0.30034 | 0.23073 | 0.80583 | 1.72408 | 0.78583 | 0.75259 |
| Calystegine B2 | 0.08109 | 0.06492 | 0.46377 | 0.84543 | 1.24910 | 0.32089 | 0.26550 |
| Capsiamide | 2.15518 | 2.52290 | 0.63953 | 0.91307 | 0.85425 | -0.22728 | 0.83967 |
| Cardoltriene | 0.12049 | 0.08868 | 0.54997 | 0.88284 | 1.35877 | 0.44230 | 0.06796 |
| Carnosine | 0.38607 | 0.23764 | 0.34859 | 0.82398 | 1.62461 | 0.70009 | 0.86758 |
| Cascarillone | 0.32829 | 0.16589 | 0.26988 | 0.81355 | 1.97905 | 0.98481 | 0.75542 |
| Casticin | 0.77337 | 0.05030 | 0.34946 | 0.82398 | 15.37605 | 3.94261 | 1.07399 |
| Cavipetin C | 0.12677 | 0.47690 | 0.06824 | 0.75227 | 0.26583 | -1.91144 | 1.31162 |
| Ceanothenic acid | 0.00403 | 0.00970 | 0.07889 | 0.75227 | 0.41574 | -1.26624 | 1.58589 |
| Cellobiose | 0.52682 | 0.48751 | 0.80045 | 0.94058 | 1.08064 | 0.11188 | 0.80818 |
| Cer(d18:1/18:1(11Z)) | 0.40125 | 0.39463 | 0.96101 | 0.98827 | 1.01679 | 0.02402 | 0.27164 |
| Cerebroside B | 0.04655 | 0.10621 | 0.25454 | 0.80583 | 0.43829 | -1.19003 | 0.84066 |
| Cervonyl carnitine | 0.03299 | 0.03397 | 0.95660 | 0.98827 | 0.97132 | -0.04198 | 0.04537 |
| Cholesta-4,6-dien-3-one | 0.35731 | 0.21533 | 0.18340 | 0.80583 | 1.65937 | 0.73063 | 0.41160 |
| Cholic acid | 3.17313 | 3.96612 | 0.58077 | 0.89986 | 0.80006 | -0.32182 | 0.31355 |
| Choline | 0.95589 | 1.63898 | 0.25366 | 0.80583 | 0.58322 | -0.77788 | 1.46974 |
| Cinnamyl cinnamate | 0.11075 | 0.15809 | 0.75655 | 0.93126 | 0.70054 | -0.51346 | 0.43361 |
| Cinncassiol A | 0.08609 | 0.10220 | 0.86970 | 0.95678 | 0.84232 | -0.24756 | 0.58260 |
| cis-Resveratrol 4'-O-glucuronide | 0.14220 | 0.02092 | 0.34502 | 0.82398 | 6.79711 | 2.76492 | 1.10976 |
| Citpressine I | 0.25595 | 0.21278 | 0.46845 | 0.84543 | 1.20287 | 0.26648 | 0.40258 |
| Citpressine II | 0.15800 | 0.20769 | 0.62699 | 0.91307 | 0.76072 | -0.39457 | 0.95808 |
| Citrusin E | 0.12521 | 0.10199 | 0.38410 | 0.82398 | 1.22765 | 0.29589 | 0.23023 |
| Clionasterol | 0.02852 | 0.02633 | 0.77666 | 0.93988 | 1.08299 | 0.11502 | 0.24993 |
| Codamine | 0.23851 | 0.06063 | 0.16119 | 0.80583 | 3.93398 | 1.97599 | 0.64161 |
| Coproporphyrin III | 0.04223 | 0.02202 | 0.10866 | 0.80583 | 1.91807 | 0.93966 | 1.25333 |
| Corchoroside B | 0.01450 | 0.02311 | 0.27806 | 0.81613 | 0.62754 | -0.67222 | 1.35941 |
| Creatinine | 54.44563 | 31.65188 | 0.18734 | 0.80583 | 1.72014 | 0.78253 | 1.30682 |
| Cycloviolaxanthin | 0.67899 | 0.69943 | 0.89757 | 0.96333 | 0.97078 | -0.04279 | 0.64181 |
| Cytarabine | 0.59229 | 0.39682 | 0.02165 | 0.75227 | 1.49257 | 0.57780 | 1.83436 |
| Cytidine 2',3'-cyclic phosphate | 0.02291 | 0.01509 | 0.06515 | 0.75227 | 1.51787 | 0.60205 | 1.70227 |
| Cytokinin B | 0.06676 | 0.13374 | 0.24390 | 0.80583 | 0.49921 | -1.00228 | 1.60564 |
| Cytosine | 4.67294 | 3.63226 | 0.23071 | 0.80583 | 1.28651 | 0.36346 | 1.19427 |
| D-1-Piperideine-2-carboxylic acid | 0.50177 | 0.57158 | 0.22058 | 0.80583 | 0.87785 | -0.18795 | 1.39079 |
| Daidzein | 0.93008 | 0.91116 | 0.96223 | 0.98827 | 1.02077 | 0.02965 | 0.07231 |
| D-Alanine | 3.70200 | 3.73113 | 0.96603 | 0.98973 | 0.99219 | -0.01131 | 0.64181 |
| Deacetyldiltiazem | 0.02641 | 0.02160 | 0.75772 | 0.93126 | 1.22291 | 0.29032 | 0.07356 |
| Dehydrophytosphingosine | 4.53129 | 5.73421 | 0.32164 | 0.82125 | 0.79022 | -0.33967 | 1.10995 |
| Delta-Tocopherol | 0.01462 | 0.02973 | 0.06004 | 0.75227 | 0.49175 | -1.02401 | 1.68866 |
| Demethoxyegonol | 0.08573 | 0.05072 | 0.21463 | 0.80583 | 1.69045 | 0.75741 | 0.49520 |
| Demethylated antipyrine | 0.18195 | 0.19056 | 0.87224 | 0.95712 | 0.95478 | -0.06675 | 0.55374 |
| Deoxyadenosine | 10.69581 | 9.32097 | 0.56681 | 0.89467 | 1.14750 | 0.19849 | 0.56833 |
| Deoxyadenosine monophosphate | 0.10923 | 0.05428 | 0.06416 | 0.75227 | 2.01245 | 1.00895 | 1.60873 |
| Deoxycholic acid | 3.60076 | 1.91042 | 0.05113 | 0.75227 | 1.88480 | 0.91441 | 1.31902 |
| Deoxycorticosterone | 0.00919 | 0.01199 | 0.35712 | 0.82398 | 0.76598 | -0.38462 | 0.60210 |
| Deoxycytidine | 0.52770 | 0.38998 | 0.14515 | 0.80583 | 1.35315 | 0.43633 | 1.41627 |
| Deoxyguanosine | 0.43380 | 0.32708 | 0.34545 | 0.82398 | 1.32628 | 0.40739 | 0.10870 |
| Deoxyinosine | 1.37348 | 1.19070 | 0.60603 | 0.91307 | 1.15351 | 0.20603 | 0.40600 |
| Dethiobiotin | 0.26109 | 0.16954 | 0.23034 | 0.80583 | 1.53996 | 0.62289 | 0.96513 |
| Deuteroporphyrin IX | 0.03396 | 0.11369 | 0.21155 | 0.80583 | 0.29871 | -1.74316 | 0.23611 |
| DG(15:0/18:4(6Z,9Z,12Z,15Z)/0:0) | 0.39442 | 0.34393 | 0.73234 | 0.92959 | 1.14682 | 0.19764 | 0.12615 |
| DG(15:0/22:6(4Z,7Z,10Z,13Z,16Z,19Z)/0:0) | 0.07729 | 0.04639 | 0.04772 | 0.75227 | 1.66626 | 0.73661 | 2.04148 |
| DG(18:4(6Z,9Z,12Z,15Z)/15:0/0:0) | 0.57791 | 0.52233 | 0.77394 | 0.93930 | 1.10639 | 0.14586 | 0.07529 |
| DG(20:4(5Z,8Z,11Z,14Z)/16:0/0:0) | 0.16678 | 0.29010 | 0.18452 | 0.80583 | 0.57490 | -0.79862 | 1.53077 |
| DG(20:5(5Z,8Z,11Z,14Z,17Z)/16:1(9Z)/0:0) | 0.06184 | 0.04324 | 0.39464 | 0.82398 | 1.43026 | 0.51627 | 1.16359 |
| DG(22:2(13Z,16Z)/18:4(6Z,9Z,12Z,15Z)/0:0) | 0.09867 | 0.03617 | 0.02149 | 0.75227 | 2.72843 | 1.44807 | 2.28304 |
| DG(22:5(7Z,10Z,13Z,16Z,19Z)/16:1(9Z)/0:0) | 1.44464 | 1.12831 | 0.14935 | 0.80583 | 1.28036 | 0.35655 | 1.63241 |
| DHAP(10:0) | 0.08531 | 0.06609 | 0.37323 | 0.82398 | 1.29098 | 0.36847 | 0.62638 |
| DHAP(18:0e) | 0.16062 | 0.11913 | 0.49175 | 0.85013 | 1.34827 | 0.43111 | 0.29969 |
| Diethanolamine | 0.25871 | 0.14633 | 0.15376 | 0.80583 | 1.76792 | 0.82205 | 1.29469 |
| Diethylcarbamazine N-oxide | 0.04281 | 0.07013 | 0.29551 | 0.81978 | 0.61037 | -0.71225 | 1.38592 |
| Dihydro-5-methyl-2(3H)-furanone | 7.43661 | 7.15410 | 0.89325 | 0.96117 | 1.03949 | 0.05587 | 0.08216 |
| Dihydroclusin | 0.15176 | 0.01018 | 0.19872 | 0.80583 | 14.90744 | 3.89796 | 1.17000 |
| Dihydroferuloylglycine | 0.10895 | 0.19469 | 0.23915 | 0.80583 | 0.55961 | -0.83750 | 1.60429 |
| Dihydrothymine | 0.96089 | 0.84501 | 0.82405 | 0.94058 | 1.13713 | 0.18540 | 0.87262 |
| Dimethyl 3-methoxy-4-oxo-5-(8,11,14-pentadecatrienyl)-2-hexenedioate | 0.03141 | 0.01305 | 0.02369 | 0.75227 | 2.40746 | 1.26751 | 1.83388 |
| Dimethylethanolamine | 0.22136 | 0.17074 | 0.15216 | 0.80583 | 1.29648 | 0.37460 | 1.24569 |
| Dipropyl sulfide | 0.50609 | 0.61967 | 0.38645 | 0.82398 | 0.81671 | -0.29210 | 1.29353 |
| DL-2-Aminooctanoic acid | 0.45681 | 0.65276 | 0.44395 | 0.84346 | 0.69982 | -0.51495 | 1.23999 |
| D-Maltose | 0.02160 | 0.01534 | 0.41249 | 0.82772 | 1.40825 | 0.49391 | 0.83845 |

| Dopamine | 0.30854 | 0.43118 | 0.26171 | 0.81355 | 0.71557 | -0.48283 | 1.33294 |
| --- | --- | --- | --- | --- | --- | --- | --- |
| Dopamine quinone | 0.30904 | 0.32803 | 0.80165 | 0.94058 | 0.94212 | -0.08602 | 0.40956 |
| D-Ornithine | 0.95890 | 1.27780 | 0.45597 | 0.84543 | 0.75043 | -0.41421 | 0.92364 |
| D-Pantethine | 0.03333 | 0.06213 | 0.32094 | 0.82125 | 0.53649 | -0.89837 | 0.54097 |
| D-Pipecolic acid | 8.87556 | 9.28258 | 0.81193 | 0.94058 | 0.95615 | -0.06469 | 1.04016 |
| D-Proline | 16.83930 | 22.29930 | 0.07977 | 0.75227 | 0.75515 | -0.40517 | 1.96905 |
| D-Serine | 0.33245 | 0.35752 | 0.82137 | 0.94058 | 0.92986 | -0.10491 | 1.15179 |
| d-Tocotrienol | 0.78729 | 0.58805 | 0.47267 | 0.84543 | 1.33880 | 0.42094 | 0.20886 |
| (E)-2',4,4',6'-Tetrahydroxy-3',5'-diprenylchalcone | 0.09733 | 0.05127 | 0.06167 | 0.75227 | 1.89833 | 0.92473 | 1.56369 |
| (E)-Casimiroedine | 0.16932 | 0.15840 | 0.91378 | 0.97075 | 1.06889 | 0.09612 | 0.42352 |
| Ecgonine | 0.18384 | 0.12682 | 0.07481 | 0.75227 | 1.44960 | 0.53566 | 0.92763 |
| (E,E)-Trichostachine | 0.19679 | 0.76550 | 0.11177 | 0.80583 | 0.25707 | -1.95976 | 0.80302 |
| Eicosapentaenoic acid | 0.14732 | 0.43992 | 0.15183 | 0.80583 | 0.33488 | -1.57829 | 1.66649 |
| Elaidic carnitine | 0.81482 | 1.02007 | 0.46220 | 0.84543 | 0.79879 | -0.32412 | 0.74585 |
| Epidermin | 0.55430 | 1.29596 | 0.11179 | 0.80583 | 0.42771 | -1.22528 | 1.24204 |
| Epsilon-(gamma-Glutamyl)-lysine | 0.12331 | 0.11201 | 0.92138 | 0.97551 | 1.10090 | 0.13869 | 0.25367 |
| Eremopetasitenin C3 | 0.07976 | 0.78675 | 0.33268 | 0.82398 | 0.10138 | -3.30219 | 1.00281 |
| Ergocalciferol | 0.14186 | 0.12475 | 0.65815 | 0.91627 | 1.13718 | 0.18546 | 0.24328 |
| Ergothioneine | 0.79943 | 0.99682 | 0.51337 | 0.86600 | 0.80198 | -0.31836 | 0.94180 |
| Erinacine P | 0.04613 | 0.09632 | 0.30071 | 0.82070 | 0.47895 | -1.06204 | 0.52587 |
| (-)-erythro-Anethole glycol 2-glucoside | 0.02170 | 0.01809 | 0.31157 | 0.82125 | 1.19932 | 0.26222 | 0.34296 |
| Ethyl 4-(acetylthio)butyrate | 0.02251 | 0.46934 | 0.28518 | 0.81978 | 0.04795 | -4.38228 | 1.04630 |
| Ethylbenzene | 0.41659 | 0.36113 | 0.24347 | 0.80583 | 1.15357 | 0.20611 | 0.59371 |
| Ethyl beta-D-glucopyranoside | 0.32118 | 0.32121 | 0.99977 | 0.99977 | 0.99991 | -0.00012 | 0.67522 |
| Ethyl N-ethylanthranilate | 0.15328 | 0.10208 | 0.52162 | 0.86996 | 1.50166 | 0.58656 | 0.80165 |
| Fagomine | 1.81157 | 0.46163 | 0.16885 | 0.80583 | 3.92430 | 1.97243 | 0.77816 |
| Falcarindiol | 0.05499 | 0.08839 | 0.24734 | 0.80583 | 0.62215 | -0.68466 | 0.64023 |
| Falimint | 0.06501 | 0.06972 | 0.78752 | 0.94058 | 0.93246 | -0.10089 | 0.74820 |
| Feruloyl-2-hydroxyputrescine | 0.15061 | 0.07541 | 0.05486 | 0.75227 | 1.99735 | 0.99809 | 1.56139 |
| Feruperine | 0.37319 | 0.10705 | 0.09107 | 0.77591 | 3.48599 | 1.80157 | 1.51346 |
| Flavidulol C | 0.02247 | 0.02844 | 0.43170 | 0.83108 | 0.79011 | -0.33987 | 0.57436 |
| Flazine | 0.36937 | 0.31790 | 0.57761 | 0.89986 | 1.16191 | 0.21650 | 0.17089 |
| fluvoxamino acid | 0.45048 | 0.42821 | 0.79290 | 0.94058 | 1.05200 | 0.07314 | 0.00281 |
| Formiminoglutamic acid | 0.13704 | 0.10720 | 0.36085 | 0.82398 | 1.27829 | 0.35421 | 0.20054 |
| Formyl-5-hydroxykynurenamine | 1.57611 | 0.42106 | 0.40077 | 0.82398 | 3.74320 | 1.90427 | 0.47958 |
| Formylfusarochromanone | 0.50255 | 0.51000 | 0.95754 | 0.98827 | 0.98539 | -0.02123 | 0.36379 |
| Frenolicin B | 0.16508 | 0.13975 | 0.54089 | 0.88040 | 1.18119 | 0.24025 | 0.29174 |
| Furcelleran | 0.03330 | 0.03420 | 0.92294 | 0.97551 | 0.97348 | -0.03878 | 0.45401 |
| Fusarochromanone | 0.02902 | 0.06476 | 0.20991 | 0.80583 | 0.44810 | -1.15811 | 1.57412 |
| Galactosylsphingosine | 0.08579 | 0.09471 | 0.74948 | 0.93126 | 0.90580 | -0.14274 | 0.67179 |
| gamma-Aminobutyric acid | 4.18750 | 4.66888 | 0.66184 | 0.91627 | 0.89690 | -0.15699 | 0.47741 |
| Gamma-Aminobutyryl-lysine | 0.05495 | 0.07709 | 0.34672 | 0.82398 | 0.71280 | -0.48844 | 1.67778 |
| gamma-Calacorene | 0.48379 | 0.32139 | 0.12215 | 0.80583 | 1.50528 | 0.59003 | 0.95832 |
| gamma-Glutamylaspartic acid | 0.04454 | 0.04274 | 0.88789 | 0.95911 | 1.04205 | 0.05942 | 0.04511 |
| gamma-Glutamylglutamic acid | 0.15966 | 0.17794 | 0.68131 | 0.92441 | 0.89724 | -0.15643 | 0.53665 |
| gamma-Glutamylleucine | 0.25879 | 0.19998 | 0.24698 | 0.80583 | 1.29411 | 0.37196 | 0.07053 |
| gamma-Glutamylmethionine | 0.04071 | 0.04336 | 0.69780 | 0.92441 | 0.93906 | -0.09072 | 0.91517 |
| gamma-Tocopheryl quinone | 0.03212 | 0.05715 | 0.21060 | 0.80583 | 0.56210 | -0.83109 | 0.75761 |
| Ganodermanondiol | 0.12073 | 0.10781 | 0.43041 | 0.83108 | 1.11981 | 0.16326 | 0.03764 |
| Genipinic acid | 0.06846 | 0.23107 | 0.36577 | 0.82398 | 0.29625 | -1.75511 | 1.08348 |
| Ginkgolide A | 0.12129 | 0.15291 | 0.50889 | 0.86434 | 0.79321 | -0.33422 | 0.98431 |
| Ginkgolide C | 0.07534 | 0.08256 | 0.88968 | 0.95974 | 0.91246 | -0.13217 | 0.98778 |
| Ginsenoyne A linoleate | 0.01911 | 0.01203 | 0.08369 | 0.75227 | 1.58850 | 0.66767 | 0.95404 |
| Glucosylceramide (d18:1/16:0) | 0.16886 | 0.16111 | 0.88638 | 0.95911 | 1.04809 | 0.06776 | 0.43129 |
| Glutaminylalanine | 0.29165 | 0.47097 | 0.45362 | 0.84543 | 0.61926 | -0.69139 | 0.88667 |
| Glutaminylhydroxyproline | 0.43809 | 0.30297 | 0.52197 | 0.86996 | 1.44598 | 0.53204 | 0.93044 |
| Glutaminylleucine | 0.10574 | 0.09151 | 0.55832 | 0.88968 | 1.15552 | 0.20854 | 0.03349 |
| Glutaminylphenylalanine | 0.02847 | 0.01807 | 0.08188 | 0.75227 | 1.57543 | 0.65575 | 1.68874 |
| Glutaminylvaline | 0.40654 | 0.74696 | 0.06569 | 0.75227 | 0.54426 | -0.87764 | 1.27948 |
| Glutamylalanine | 0.69131 | 0.53658 | 0.34169 | 0.82398 | 1.28835 | 0.36552 | 1.09545 |
| Glutamyllysine | 0.03215 | 0.07103 | 0.16103 | 0.80583 | 0.45266 | -1.14349 | 1.28013 |
| Glutamylthreonine | 0.11121 | 0.13028 | 0.58670 | 0.90387 | 0.85361 | -0.22835 | 0.32675 |
| Glycerol tributanoate | 0.06794 | 0.07149 | 0.86055 | 0.95530 | 0.95035 | -0.07347 | 0.55329 |
| Glycerol trihexanoate | 0.16293 | 0.17171 | 0.77157 | 0.93930 | 0.94884 | -0.07576 | 0.45939 |
| Glycerophosphocholine | 0.12886 | 0.34781 | 0.12326 | 0.80583 | 0.37049 | -1.43250 | 1.24208 |
| Glycerylphosphorylethanolamine | 0.08977 | 0.17359 | 0.22875 | 0.80583 | 0.51713 | -0.95139 | 1.30342 |
| Glycylleucine | 2.01742 | 2.05319 | 0.92268 | 0.97551 | 0.98258 | -0.02536 | 0.47541 |
| Glycyl-Phenylalanine | 0.75143 | 0.67771 | 0.87855 | 0.95744 | 1.10878 | 0.14897 | 0.90675 |
| Glycylprolylhydroxyproline | 0.10229 | 0.06833 | 0.39657 | 0.82398 | 1.49699 | 0.58207 | 0.70616 |
| Glycyl-Valine | 0.87293 | 0.90267 | 0.87852 | 0.95744 | 0.96705 | -0.04834 | 1.10023 |
| Glycyrrhetinic acid | 0.19231 | 0.15760 | 0.47251 | 0.84543 | 1.22024 | 0.28717 | 0.04386 |
| Goyaglycoside c | 0.76455 | 0.49622 | 0.04167 | 0.75227 | 1.54074 | 0.62363 | 1.11782 |
| Guanine | 4.12525 | 3.69182 | 0.60528 | 0.91307 | 1.11740 | 0.16015 | 0.16410 |
| Guanosine | 0.67152 | 0.59457 | 0.70190 | 0.92441 | 1.12942 | 0.17559 | 0.23729 |
| Gyromitrin | 3.63086 | 6.60800 | 0.03806 | 0.75227 | 0.54946 | -0.86390 | 1.73817 |
| Harman | 3.81064 | 6.31989 | 0.03986 | 0.75227 | 0.60296 | -0.72987 | 1.34397 |
| Hepoxilin A3 | 0.09709 | 0.04071 | 0.05990 | 0.75227 | 2.38497 | 1.25397 | 1.56247 |
| Heptadecanoyl carnitine | 0.05608 | 0.11643 | 0.13515 | 0.80583 | 0.48162 | -1.05404 | 1.75641 |
| Heptyl cinnamate | 0.90964 | 0.50759 | 0.05571 | 0.75227 | 1.79207 | 0.84163 | 1.16360 |
| hesperetin 3'-O-sulfate | 0.08706 | 0.07173 | 0.84244 | 0.95251 | 1.21379 | 0.27952 | 0.17369 |
| Hexanoylglycine | 0.10176 | 0.23275 | 0.16773 | 0.80583 | 0.43718 | -1.19369 | 1.52987 |
| Hirsutin | 0.02935 | 0.04368 | 0.12088 | 0.80583 | 0.67197 | -0.57353 | 1.20914 |
| Histamine | 2.02704 | 0.65567 | 0.18218 | 0.80583 | 3.09157 | 1.62834 | 1.13164 |
| Histidinal | 1.50132 | 1.53537 | 0.95435 | 0.98827 | 0.97783 | -0.03235 | 0.43106 |
| Histidinyl-Gamma-glutamate | 0.03733 | 0.04472 | 0.42200 | 0.82772 | 0.83488 | -0.26036 | 1.41768 |
| Histidinyl-Leucine | 0.86436 | 0.94226 | 0.61397 | 0.91307 | 0.91732 | -0.12450 | 0.83158 |
| Histidinyl-Proline | 0.17577 | 0.19630 | 0.67279 | 0.91945 | 0.89542 | -0.15937 | 1.19230 |
| Homoanserine | 0.45107 | 0.41380 | 0.86513 | 0.95561 | 1.09006 | 0.12441 | 0.67358 |
| Hovenidulcigenin B | 0.07216 | 0.05400 | 0.25417 | 0.80583 | 1.33624 | 0.41818 | 0.80442 |

| Hydroxyisocaproic acid | 0.06376 | 0.05411 | 0.24789 | 0.80583 | 1.17821 | 0.23660 | 0.58347 |
| --- | --- | --- | --- | --- | --- | --- | --- |
| Hydroxyprolyl-Hydroxyproline | 0.25133 | 0.34215 | 0.14207 | 0.80583 | 0.73457 | -0.44502 | 1.80631 |
| Hydroxyprolyl-Isoleucine | 1.12536 | 4.29583 | 0.08043 | 0.75227 | 0.26196 | -1.93256 | 0.91428 |
| Hydroxyprolyl-Lysine | 1.01243 | 0.92085 | 0.53076 | 0.87495 | 1.09945 | 0.13678 | 0.40401 |
| Hydroxyprolyl-Valine | 0.19270 | 0.16963 | 0.75459 | 0.93126 | 1.13596 | 0.18391 | 0.40815 |
| Hypoglycin B | 0.36585 | 0.22936 | 0.16154 | 0.80583 | 1.59512 | 0.67366 | 0.50965 |
| Hypoletin 8-gentiobioside | 0.11882 | 0.01820 | 0.24736 | 0.80583 | 6.52874 | 2.70680 | 0.52243 |
| Hypoxanthine | 200.35531 | 191.63170 | 0.70975 | 0.92626 | 1.04552 | 0.06422 | 0.64857 |
| Indole-3-carboxylic acid | 0.03958 | 0.05256 | 0.20433 | 0.80583 | 0.75301 | -0.40925 | 1.18111 |
| Indoleacetaldehyde | 0.27114 | 0.12758 | 0.34117 | 0.82398 | 2.12532 | 1.08768 | 1.10805 |
| Indoleacetic acid | 0.04409 | 0.09160 | 0.23695 | 0.80583 | 0.48136 | -1.05483 | 1.54157 |
| Inosine | 1.17808 | 0.40472 | 0.00028 | 0.23068 | 2.91084 | 1.54144 | 2.73243 |
| Isocorydine | 4.41869 | 2.16533 | 0.26470 | 0.81355 | 2.04065 | 1.02903 | 0.39282 |
| Isoleucyl-Asparagine | 0.39765 | 0.45585 | 0.37996 | 0.82398 | 0.87232 | -0.19708 | 1.37152 |
| Isoleucyl-Phenylalanine | 0.04104 | 0.04998 | 0.49531 | 0.85451 | 0.82104 | -0.28447 | 0.76971 |
| Isoleucyl-Tryptophan | 0.58331 | 0.60724 | 0.85063 | 0.95269 | 0.96058 | -0.05802 | 0.54605 |
| Isoleucyl-Tyrosine | 0.39589 | 0.09343 | 0.22328 | 0.80583 | 4.23731 | 2.08315 | 1.18742 |
| Isomasticadienonic acid | 0.27483 | 0.20041 | 0.56899 | 0.89551 | 1.37138 | 0.45563 | 0.31315 |
| Isonicotinic acid | 0.23599 | 0.23672 | 0.98810 | 0.99766 | 0.99694 | -0.00443 | 0.19541 |
| Isopetasoside | 0.02440 | 0.02724 | 0.54885 | 0.88284 | 0.89562 | -0.15904 | 0.84551 |
| Isoquinoline | 0.45331 | 0.14099 | 0.18308 | 0.80583 | 3.21524 | 1.68493 | 1.13462 |
| Isosalicin | 0.33174 | 38.89757 | 0.27379 | 0.81510 | 0.00853 | -6.87350 | 1.23266 |
| Isotetrandrine | 0.04682 | 0.05499 | 0.59567 | 0.91097 | 0.85140 | -0.23209 | 1.02216 |
| Isovalerylalanine | 0.15402 | 0.12211 | 0.45377 | 0.84543 | 1.26138 | 0.33500 | 0.08580 |
| Isovalerylglucuronide | 0.01416 | 0.03976 | 0.09358 | 0.78926 | 0.35617 | -1.48937 | 1.71234 |
| I-Urobilin | 0.85369 | 1.53662 | 0.21041 | 0.80583 | 0.55557 | -0.84797 | 1.47337 |
| Jasmolone | 0.43883 | 0.41705 | 0.80466 | 0.94058 | 1.05222 | 0.07343 | 0.37786 |
| Junosine | 0.02532 | 0.09795 | 0.00802 | 0.62473 | 0.25855 | -1.95149 | 1.89235 |
| Kaempferol | 0.02307 | 0.09731 | 0.06081 | 0.75227 | 0.23709 | -2.07648 | 1.10342 |
| Kamahine C | 0.15884 | 0.04802 | 0.38158 | 0.82398 | 3.30814 | 1.72602 | 0.31541 |
| Kinobeon A | 0.01594 | 0.02935 | 0.39743 | 0.82398 | 0.54307 | -0.88078 | 0.30856 |
| Kuwanon E | 0.04840 | 0.02504 | 0.12018 | 0.80583 | 1.93254 | 0.95050 | 1.34676 |
| Kynurenic acid | 0.55990 | 0.33618 | 0.18588 | 0.80583 | 1.66551 | 0.73597 | 0.45449 |
| L-1,2,3,4-Tetrahydro-beta-carboline-3-carboxylic acid | 0.09874 | 0.09494 | 0.84910 | 0.95269 | 1.03998 | 0.05656 | 0.88825 |
| L-2-Amino-5-hydroxypentanoic acid | 0.53951 | 0.49185 | 0.71150 | 0.92637 | 1.09689 | 0.13342 | 0.44249 |
| Laccarin | 0.10639 | 0.08254 | 0.47464 | 0.84543 | 1.28887 | 0.36611 | 0.35643 |
| Lactosylceramide (d18:1/16:0) | 0.05490 | 0.24408 | 0.00120 | 0.43316 | 0.22492 | -2.15253 | 1.94345 |
| L-Allothreonine | 1.08101 | 0.77488 | 0.41076 | 0.82772 | 1.39507 | 0.48034 | 0.73742 |
| L-alpha-Amino-1H-pyrrole-1-hexanoic acid | 0.06221 | 0.06412 | 0.97284 | 0.99296 | 0.97020 | -0.04364 | 0.05877 |
| L-alpha-Aminobutyric acid | 0.91194 | 1.20426 | 0.38228 | 0.82398 | 0.75726 | -0.40113 | 1.59906 |
| L-Asparagine | 0.20242 | 0.14678 | 0.64407 | 0.91307 | 1.37900 | 0.46362 | 0.39153 |
| L-Aspartic acid | 0.53685 | 0.47574 | 0.64737 | 0.91556 | 1.12846 | 0.17436 | 0.38731 |
| L-beta-aspartyl-L-glutamic acid | 0.09345 | 0.15027 | 0.24606 | 0.80583 | 0.62189 | -0.68527 | 0.87400 |
| L-Carnitine | 5.95761 | 6.94214 | 0.60350 | 0.91307 | 0.85818 | -0.22065 | 1.16255 |
| Leucyl-Arginine | 0.89804 | 0.93249 | 0.82456 | 0.94058 | 0.96306 | -0.05430 | 0.55206 |
| Leucyl-Glycine | 0.24668 | 0.41559 | 0.05103 | 0.75227 | 0.59357 | -0.75251 | 2.04869 |
| Leucyl-Hydroxyproline | 0.24679 | 0.07834 | 0.21356 | 0.80583 | 3.15013 | 1.65541 | 1.04003 |
| Leucyl-Isoleucine | 15.86245 | 15.55658 | 0.91240 | 0.97071 | 1.01966 | 0.02809 | 0.00533 |
| Leucyl-Serine | 0.78639 | 0.62824 | 0.26797 | 0.81355 | 1.25173 | 0.32392 | 0.42762 |
| Leucyl-Threonine | 2.66161 | 2.53727 | 0.79541 | 0.94058 | 1.04901 | 0.06903 | 0.25461 |
| Leucyl-Valine | 15.76280 | 13.90928 | 0.46666 | 0.84543 | 1.13326 | 0.18048 | 0.35884 |
| L-Furosine | 0.17785 | 0.09344 | 0.25422 | 0.80583 | 1.90333 | 0.92853 | 0.97417 |
| L-Glutamic acid | 25.50256 | 24.87728 | 0.88766 | 0.95911 | 1.02513 | 0.03581 | 0.65165 |
| L-Histidinol | 0.13301 | 0.08083 | 0.45440 | 0.84543 | 1.64566 | 0.71867 | 0.20924 |
| Linalyl phenylacetate | 0.05386 | 0.06112 | 0.69503 | 0.92441 | 0.88109 | -0.18264 | 0.19398 |
| Linamarin | 0.07441 | 0.08293 | 0.69465 | 0.92441 | 0.89726 | -0.15641 | 0.68297 |
| Linoleamide | 4.24680 | 3.43470 | 0.63700 | 0.91307 | 1.23644 | 0.30619 | 0.05803 |
| Linoleoyl ethanolamide | 3.85832 | 6.40156 | 0.14571 | 0.80583 | 0.60272 | -0.73045 | 1.39997 |
| L-Isoleucine | 0.40794 | 0.54231 | 0.22337 | 0.80583 | 0.75224 | -0.41074 | 1.22249 |
| Lithocholyltaurine | 0.03345 | 0.03832 | 0.66562 | 0.91627 | 0.87295 | -0.19603 | 0.74213 |
| L,L-Cyclo(leucylprolyl) | 0.03685 | 0.04252 | 0.81406 | 0.94058 | 0.86675 | -0.20631 | 0.20233 |
| L-Leucine | 0.16460 | 0.17680 | 0.80023 | 0.94058 | 0.93100 | -0.10315 | 0.49445 |
| L-L-Homoglutathione | 0.14425 | 0.02111 | 0.30904 | 0.82125 | 6.83434 | 2.77280 | 1.01422 |
| L-Lysine | 7.99323 | 6.85274 | 0.59247 | 0.90940 | 1.16643 | 0.22210 | 0.55543 |
| L-Lysopine | 0.66780 | 0.44906 | 0.19906 | 0.80583 | 1.48713 | 0.57253 | 0.81920 |
| L-Methionine | 6.39658 | 7.26275 | 0.54404 | 0.88103 | 0.88074 | -0.18321 | 1.39117 |
| Loquatoside | 0.28074 | 0.29373 | 0.92710 | 0.97743 | 0.95577 | -0.06527 | 0.38980 |
| L-Palmitoylcarnitine | 1.20265 | 2.11017 | 0.19091 | 0.80583 | 0.56993 | -0.81114 | 1.18179 |
| L-Phenylalanine | 23.35944 | 23.52802 | 0.97736 | 0.99353 | 0.99284 | -0.01037 | 0.44014 |
| L-Proline | 0.47354 | 0.43357 | 0.63468 | 0.91307 | 1.09220 | 0.12723 | 0.20344 |
| L-prolyl-L-proline | 0.12359 | 0.12438 | 0.97925 | 0.99353 | 0.99363 | -0.00922 | 0.60544 |
| L-Pyridosine | 0.29128 | 0.35044 | 0.61461 | 0.91307 | 0.83117 | -0.26679 | 1.29117 |
| L-Theanine | 0.34370 | 0.34667 | 0.95840 | 0.98827 | 0.99144 | -0.01240 | 0.42679 |
| L-Threonine | 0.90299 | 0.79357 | 0.63814 | 0.91307 | 1.13788 | 0.18635 | 0.53393 |
| L-Tyrosine | 5.72517 | 6.93942 | 0.41245 | 0.82772 | 0.82502 | -0.27750 | 1.45432 |
| L-Urobilin | 2.53174 | 1.39823 | 0.11606 | 0.80583 | 1.81067 | 0.85653 | 1.22098 |
| Lutein | 1.44796 | 1.99582 | 0.36549 | 0.82398 | 0.72550 | -0.46295 | 1.41474 |
| L-Valine | 13.13521 | 15.55089 | 0.43196 | 0.83108 | 0.84466 | -0.24356 | 1.30219 |
| Lycopene | 0.17746 | 0.34010 | 0.00847 | 0.62473 | 0.52177 | -0.93851 | 2.61757 |
| Lycoperoside D | 0.05205 | 0.05619 | 0.84757 | 0.95251 | 0.92637 | -0.11034 | 0.44685 |
| Lysinoalanine | 0.05112 | 0.04051 | 0.16974 | 0.80583 | 1.26203 | 0.33574 | 0.71943 |
| LysoPC(14:1(9Z)) | 0.18705 | 0.08321 | 0.02615 | 0.75227 | 2.24787 | 1.16856 | 1.47108 |
| LysoPC(16:0) | 8.02975 | 10.38693 | 0.54439 | 0.88103 | 0.77306 | -0.37134 | 0.25709 |
| LysoPC(20:3(5Z,8Z,11Z)) | 0.03544 | 0.03319 | 0.79287 | 0.94058 | 1.06760 | 0.09437 | 0.61344 |
| LysoPC(20:4(5Z,8Z,11Z,14Z)) | 0.01498 | 0.03071 | 0.31315 | 0.82125 | 0.48787 | -1.03542 | 0.55939 |
| LysoPC(22:0) | 0.02446 | 0.02990 | 0.53290 | 0.87495 | 0.81815 | -0.28956 | 0.71363 |
| LysoPC(P-18:1(9Z)) | 0.02242 | 0.01323 | 0.23264 | 0.80583 | 1.69407 | 0.76049 | 1.07918 |
| LysoPE(0:0/18:3(6Z,9Z,12Z)) | 0.08179 | 0.08257 | 0.94743 | 0.98827 | 0.99059 | -0.01364 | 0.63783 |
| LysoPE(15:0/0:0) | 10.19489 | 8.39055 | 0.26542 | 0.81355 | 1.21504 | 0.28101 | 0.89469 |

| LysoPE(16:0/0:0) | 13.46569 | 12.12954 | 0.51927 | 0.86996 | 1.11016 | 0.15076 | 0.65481 |
| --- | --- | --- | --- | --- | --- | --- | --- |
| LysoPE(16:1(9Z)/0:0) | 0.29128 | 0.19127 | 0.06073 | 0.75227 | 1.52284 | 0.60677 | 1.57183 |
| LysoPE(18:1(9Z)/0:0) | 1.21039 | 1.28294 | 0.85989 | 0.95530 | 0.94345 | -0.08398 | 0.11768 |
| LysoPE(18:2(9Z,12Z)/0:0) | 0.35586 | 0.42609 | 0.66651 | 0.91627 | 0.83518 | -0.25984 | 0.21367 |
| LysoPI(18:0/0:0) | 0.02404 | 0.07009 | 0.10271 | 0.80583 | 0.34299 | -1.54375 | 1.10685 |
| Lysyl-Alanine | 0.51154 | 0.43192 | 0.35780 | 0.82398 | 1.18433 | 0.24407 | 0.06182 |
| Lysyl-Asparagine | 0.13817 | 0.12552 | 0.77594 | 0.93988 | 1.10078 | 0.13853 | 0.79272 |
| Lysyl-Aspartate | 0.07063 | 0.06827 | 0.93438 | 0.98139 | 1.03453 | 0.04898 | 0.54423 |
| Lysyl-Gamma-glutamate | 0.15075 | 0.13601 | 0.71622 | 0.92871 | 1.10840 | 0.14848 | 0.61772 |
| Lysyl-Glycine | 0.24980 | 0.32193 | 0.29332 | 0.81978 | 0.77594 | -0.36598 | 1.05173 |
| Lysyl-Isoleucine | 0.29788 | 0.39180 | 0.35116 | 0.82398 | 0.76030 | -0.39536 | 1.13943 |
| Lysyl-Leucine | 0.78182 | 0.83844 | 0.73081 | 0.92959 | 0.93247 | -0.10087 | 0.66194 |
| Lysyl-Lysine | 0.03381 | 0.02576 | 0.46128 | 0.84543 | 1.31243 | 0.39224 | 0.21270 |
| Lysyl-Phenylalanine | 0.06155 | 0.05894 | 0.81853 | 0.94058 | 1.04435 | 0.06260 | 0.45951 |
| Lysyl-Threonine | 0.14404 | 0.07357 | 0.21925 | 0.80583 | 1.95771 | 0.96916 | 0.21463 |
| Lysyl-Tyrosine | 0.03762 | 0.04124 | 0.61193 | 0.91307 | 0.91226 | -0.13249 | 0.62613 |
| Lysyl-Valine | 7.21416 | 7.29577 | 0.95467 | 0.98827 | 0.98881 | -0.01623 | 0.07005 |
| m-Aminobenzoic acid | 0.73930 | 0.70729 | 0.88076 | 0.95744 | 1.04526 | 0.06386 | 0.44454 |
| Mangiferdesmethylursanone | 2.34142 | 4.42274 | 0.20758 | 0.80583 | 0.52941 | -0.91755 | 1.37744 |
| Margrapine A | 0.00865 | 0.25279 | 0.18847 | 0.80583 | 0.03423 | -4.86876 | 0.78804 |
| Mascaroside | 0.02363 | 0.02854 | 0.54550 | 0.88103 | 0.82806 | -0.27219 | 0.99644 |
| Mecarbam | 0.03393 | 0.06073 | 0.14828 | 0.80583 | 0.55877 | -0.83967 | 1.62008 |
| Medicagenic acid | 0.10340 | 0.06639 | 0.02311 | 0.75227 | 1.55750 | 0.63923 | 1.57956 |
| Melibiose | 0.22935 | 0.24850 | 0.72393 | 0.92871 | 0.92293 | -0.11570 | 0.50835 |
| Menthone 1,2-glyceryl ketal | 0.02433 | 0.02012 | 0.40449 | 0.82398 | 1.20942 | 0.27431 | 0.84134 |
| Mesoporphyrin IX | 0.18963 | 0.02134 | 0.22349 | 0.80583 | 8.88707 | 3.15171 | 0.71582 |
| Metenamine | 1.76583 | 1.59328 | 0.21561 | 0.80583 | 1.10830 | 0.14835 | 0.93561 |
| Methionine sulfoxide | 0.73458 | 0.78403 | 0.81643 | 0.94058 | 0.93693 | -0.09399 | 1.08401 |
| Methionyl-Arginine | 0.06455 | 0.05450 | 0.40108 | 0.82398 | 1.18428 | 0.24401 | 0.72043 |
| Methionyl-Isoleucine | 1.69313 | 1.49485 | 0.50939 | 0.86434 | 1.13265 | 0.17970 | 0.54813 |
| Methionyl-Methionine | 0.05615 | 0.06024 | 0.70189 | 0.92441 | 0.93208 | -0.10147 | 0.65575 |
| Methionyl-Proline | 0.12779 | 0.10347 | 0.31712 | 0.82125 | 1.23503 | 0.30454 | 0.18948 |
| Methionyl-Threonine | 0.12952 | 0.12990 | 0.99184 | 0.99901 | 0.99705 | -0.00426 | 0.86038 |
| Methionyl-Tyrosine | 0.05270 | 0.04422 | 0.42646 | 0.83108 | 1.19177 | 0.25310 | 0.13143 |
| Methionyl-Valine | 0.74616 | 0.64710 | 0.42042 | 0.82772 | 1.15309 | 0.20550 | 0.69654 |
| Methoxypyrazine | 2.84188 | 2.80692 | 0.86263 | 0.95530 | 1.01246 | 0.01786 | 0.56476 |
| Methyl 6-O-galloyl-beta-D-glucopyranoside | 0.22048 | 0.20680 | 0.83383 | 0.94728 | 1.06615 | 0.09241 | 0.54945 |
| Methyl 7-epi-12-hydroxyjasmonate glucoside | 0.38634 | 0.05689 | 0.22576 | 0.80583 | 6.79123 | 2.76367 | 1.10130 |
| Methyldopa | 0.16409 | 0.16398 | 0.99879 | 0.99977 | 1.00067 | 0.00097 | 0.63633 |
| Methylimidazole acetaldehyde | 0.07480 | 0.07679 | 0.88079 | 0.95744 | 0.97402 | -0.03798 | 0.63779 |
| Methylpyrazine | 7.89026 | 2.86257 | 0.16375 | 0.80583 | 2.75636 | 1.46276 | 1.19255 |
| MG(0:0/18:3(6Z,9Z,12Z)/0:0) | 0.72715 | 0.85871 | 0.70265 | 0.92441 | 0.84679 | -0.23992 | 1.33156 |
| MG(0:0/18:4(6Z,9Z,12Z,15Z)/0:0) | 0.31417 | 0.27229 | 0.82310 | 0.94058 | 1.15381 | 0.20640 | 0.74756 |
| MG(18:3(6Z,9Z,12Z)/0:0/0:0) | 0.06084 | 0.20052 | 0.21352 | 0.80583 | 0.30340 | -1.72070 | 0.55688 |
| Montecristin | 0.06040 | 0.08316 | 0.70405 | 0.92441 | 0.72628 | -0.46141 | 0.34509 |
| Morpholine | 0.96271 | 0.79915 | 0.19755 | 0.80583 | 1.20466 | 0.26862 | 1.02433 |
| Muramic acid | 0.20071 | 0.10614 | 0.16217 | 0.80583 | 1.89103 | 0.91917 | 1.19270 |
| Mycotoxin T 2 | 0.71451 | 0.56672 | 0.76200 | 0.93432 | 1.26078 | 0.33432 | 0.13372 |
| Mytilin A | 0.17951 | 0.02440 | 0.27154 | 0.81355 | 7.35773 | 2.87926 | 0.53918 |
| N1-Caffeoyl-N10-feruloylspermidine | 0.07357 | 0.07167 | 0.95940 | 0.98827 | 1.02641 | 0.03760 | 0.44689 |
| N1-Methyl-4-pyridone-3-carboxamide | 0.20535 | 0.29379 | 0.20722 | 0.80583 | 0.69897 | -0.51669 | 1.22777 |
| N2-(2-Carboxymethyl-2-hydroxysuccinoyl)arginine | 0.04595 | 0.03616 | 0.49761 | 0.85556 | 1.27060 | 0.34551 | 0.60406 |
| N-(2-Methylpropyl)acetamide | 0.12638 | 0.23918 | 0.22729 | 0.80583 | 0.52838 | -0.92036 | 1.53978 |
| N2-Succinoylarginine | 0.06728 | 0.06678 | 0.97416 | 0.99296 | 1.00750 | 0.01079 | 0.97724 |
| N-(4,5-Dihydro-1-methyl-4-oxo-1H-imidazol-2-yl)alanine | 1.90817 | 1.57499 | 0.40555 | 0.82398 | 1.21154 | 0.27685 | 0.68961 |
| N-[(4E,8Z)-1,3-dihydroxyoctadeca-4,8-dien-2-yl]hexadecanamide 1-glucoside | 0.18010 | 0.14156 | 0.22752 | 0.80583 | 1.27224 | 0.34737 | 0.83353 |
| N-[(4-Hydroxy-3-methoxyphenyl)methyl]octanamide | 0.03606 | 0.02494 | 0.38323 | 0.82398 | 1.44616 | 0.53222 | 0.70538 |
| N6-Galacturonyl-L-lysine | 0.08162 | 0.13207 | 0.29473 | 0.81978 | 0.61804 | -0.69424 | 1.10490 |
| N6-Methyladenosine | 0.09065 | 0.18228 | 0.09906 | 0.80583 | 0.49732 | -1.00775 | 1.86373 |
| N-a-Acetyl-L-arginine | 1.30768 | 1.72727 | 0.14635 | 0.80583 | 0.75708 | -0.40149 | 1.73065 |
| N-acetyl-5-aminosalicylic acid | 0.13409 | 0.09441 | 0.31697 | 0.82125 | 1.42031 | 0.50621 | 0.80255 |
| N-Acetyl-a-neuraminic acid | 0.11165 | 0.07149 | 0.53545 | 0.87495 | 1.56183 | 0.64324 | 0.66668 |
| N-Acetyldopamine | 0.17664 | 0.24408 | 0.37199 | 0.82398 | 0.72371 | -0.46653 | 1.49623 |
| N-Acetylgalactosamine 4-sulphate | 0.02240 | 0.03054 | 0.16656 | 0.80583 | 0.73329 | -0.44754 | 1.66111 |
| N-Acetylhistamine | 116.67860 | 52.63697 | 0.14579 | 0.80583 | 2.21667 | 1.14839 | 1.22530 |
| N-Acetylhistidine | 0.25722 | 0.20165 | 0.61932 | 0.91307 | 1.27559 | 0.35116 | 0.06627 |
| N-Acetyl-L-glutamate 5-semialdehyde | 0.70388 | 0.87775 | 0.49797 | 0.85556 | 0.80192 | -0.31847 | 0.77781 |
| N-Acetyl-L-tyrosine | 0.20993 | 0.11488 | 0.19061 | 0.80583 | 1.82733 | 0.86973 | 0.73958 |
| N-Acetylmuramate | 0.19336 | 0.10229 | 0.08385 | 0.75227 | 1.89020 | 0.91854 | 1.47701 |
| N-Acetylneuraminic acid | 0.42376 | 0.22042 | 0.13134 | 0.80583 | 1.92251 | 0.94299 | 1.49458 |
| N-Acetylornithine | 0.63582 | 0.63754 | 0.99138 | 0.99901 | 0.99730 | -0.00391 | 0.13799 |
| N-Acetylputrescine | 0.27939 | 0.90181 | 0.09953 | 0.80583 | 0.30981 | -1.69053 | 0.91953 |
| N-Acetylvaline | 0.67888 | 0.46299 | 0.27430 | 0.81510 | 1.46630 | 0.55218 | 0.58875 |
| NAD | 0.00649 | 0.00173 | 0.13637 | 0.80583 | 3.75959 | 1.91058 | 1.17000 |
| N-Alpha-acetyllysine | 2.63362 | 2.56506 | 0.89078 | 0.95974 | 1.02673 | 0.03806 | 0.69552 |
| Nandrolone | 0.77367 | 0.45804 | 0.05147 | 0.75227 | 1.68909 | 0.75625 | 1.26363 |
| N-Benzoylaspartic acid | 0.06303 | 0.09335 | 0.14006 | 0.80583 | 0.67517 | -0.56668 | 1.76277 |
| N-Cyclopropyl-trans-2-cis-6-nonadienamide | 0.23627 | 0.24673 | 0.46741 | 0.84543 | 0.95763 | -0.06246 | 1.06092 |
| Neotrehalose | 0.29821 | 0.22161 | 0.60124 | 0.91307 | 1.34569 | 0.42834 | 0.35283 |
| Nerolidol 3-O-[a-L-Rhamnopyranosyl-(1->4)-a-L-rhamnopyranosyl-(1->2)-b-D-gluc | 0.01047 | 0.01305 | 0.48682 | 0.84975 | 0.80193 | -0.31845 | 0.20899 |
| N-Ethylglycine | 1.03910 | 1.20367 | 0.63151 | 0.91307 | 0.86328 | -0.21210 | 1.26634 |
| N'-Formylkynurenine | 0.04407 | 0.03852 | 0.42848 | 0.83108 | 1.14413 | 0.19425 | 0.50458 |
| N-(Heptan-4-yl)benzo[d][1,3]dioxole-5-carboxamide | 0.08096 | 0.10497 | 0.49051 | 0.84975 | 0.77134 | -0.37457 | 0.74731 |
| N-Hexadecanoylpyrrolidine | 0.32910 | 0.43596 | 0.67243 | 0.91945 | 0.75488 | -0.40567 | 0.17449 |
| N-Hydroxy-L-tyrosine | 0.63506 | 0.62153 | 0.90101 | 0.96578 | 1.02177 | 0.03107 | 0.02583 |
| Niacinamide | 0.20582 | 0.08752 | 0.00898 | 0.62473 | 2.35173 | 1.23372 | 1.85533 |
| Nicotinic acid | 0.93627 | 0.60000 | 0.13367 | 0.80583 | 1.56046 | 0.64197 | 1.56917 |
| Nicotinic acid mononucleotide | 0.84753 | 0.63576 | 0.28666 | 0.81978 | 1.33310 | 0.41478 | 1.23950 |
| Nigakinone | 0.14566 | 0.11304 | 0.37761 | 0.82398 | 1.28865 | 0.36586 | 0.17724 |

| N-Lactoyl ethanolamine | 0.10382 | 0.67382 | 0.08469 | 0.75227 | 0.15408 | -2.69828 | 1.52500 |
| --- | --- | --- | --- | --- | --- | --- | --- |
| N-Methyl-1-deoxynojirimycin | 0.31179 | 0.14986 | 0.48505 | 0.84975 | 2.08054 | 1.05696 | 0.19873 |
| N-Methylcalystegine B2 | 0.10221 | 0.20360 | 0.06440 | 0.75227 | 0.50202 | -0.99419 | 1.77531 |
| N-Methylcoclaurine | 0.07868 | 0.04698 | 0.64030 | 0.91307 | 1.67490 | 0.74408 | 0.07019 |
| N-Methylnicotinamide | 7.22556 | 4.44573 | 0.08296 | 0.75227 | 1.62528 | 0.70069 | 1.52397 |
| N,N-Dimethylsphingosine | 0.41477 | 0.45946 | 0.86213 | 0.95530 | 0.90273 | -0.14763 | 0.00982 |
| N-Oleoylethanolamine | 1.09801 | 2.38120 | 0.04801 | 0.75227 | 0.46112 | -1.11679 | 1.94622 |
| Norambreinolide | 0.13111 | 0.07325 | 0.27875 | 0.81613 | 1.78989 | 0.83987 | 0.65075 |
| Norcapsaicin | 0.03959 | 0.04148 | 0.86080 | 0.95530 | 0.95459 | -0.06704 | 0.36091 |
| Norhydromorphone | 0.06797 | 0.18436 | 0.06266 | 0.75227 | 0.36870 | -1.43947 | 1.05941 |
| Norophthalmic acid | 1.05572 | 0.49716 | 0.01593 | 0.73899 | 2.12349 | 1.08644 | 1.58903 |
| Norpropoxyphene | 0.04012 | 0.03742 | 0.80735 | 0.94058 | 1.07239 | 0.10083 | 0.25091 |
| Nor-psi-tropine | 0.36995 | 0.42253 | 0.78465 | 0.94020 | 0.87556 | -0.19172 | 0.00473 |
| N-Palmitoylsphingosine | 0.54458 | 0.34643 | 0.01167 | 0.69594 | 1.57197 | 0.65257 | 1.81223 |
| N-Succinyl-2-amino-6-ketopimelate | 0.04572 | 0.04131 | 0.64311 | 0.91307 | 1.10668 | 0.14624 | 0.06840 |
| N-Succinyl-L,L-2,6-diaminopimelate | 0.44381 | 1.04319 | 0.37796 | 0.82398 | 0.42543 | -1.23299 | 0.77564 |
| N-Undecylbenzenesulfonic acid | 0.02799 | 0.02524 | 0.77964 | 0.93997 | 1.10880 | 0.14900 | 0.30900 |
| Nutriacholic acid | 0.13998 | 0.19121 | 0.40460 | 0.82398 | 0.73209 | -0.44991 | 0.57236 |
| O-Acetylethanolamine | 0.44200 | 0.15360 | 0.00278 | 0.43316 | 2.87749 | 1.52481 | 2.58225 |
| Obtusilactone A | 0.11331 | 0.14380 | 0.64813 | 0.91556 | 0.78792 | -0.34389 | 1.23250 |
| Octadecanamide | 0.47048 | 0.25555 | 0.01692 | 0.74378 | 1.84102 | 0.88051 | 1.16669 |
| Octadecyl cis-p-coumarate | 0.08253 | 0.14067 | 0.10154 | 0.80583 | 0.58666 | -0.76941 | 1.44872 |
| Oenanthoside A | 0.06038 | 0.04667 | 0.34532 | 0.82398 | 1.29392 | 0.37175 | 0.36264 |
| o-Ethyltoluene | 0.46282 | 0.43542 | 0.69657 | 0.92441 | 1.06292 | 0.08804 | 0.13443 |
| Oleamide | 0.12454 | 0.09241 | 0.06344 | 0.75227 | 1.34778 | 0.43059 | 1.46916 |
| Oleic acid | 0.08952 | 0.12546 | 0.41465 | 0.82772 | 0.71352 | -0.48698 | 1.06254 |
| Olomoucine | 0.05708 | 0.04198 | 0.28051 | 0.81613 | 1.35949 | 0.44306 | 0.86861 |
| O-Phosphoethanolamine | 0.93479 | 0.48419 | 0.00724 | 0.62473 | 1.93064 | 0.94908 | 2.48747 |
| Ophthalmic acid | 0.56639 | 0.52058 | 0.76980 | 0.93930 | 1.08801 | 0.12169 | 0.39308 |
| O-propanoyl-carnitine | 0.12384 | 0.15602 | 0.19384 | 0.80583 | 0.79379 | -0.33317 | 1.57827 |
| Ornithine | 0.43143 | 0.37031 | 0.53295 | 0.87495 | 1.16505 | 0.22040 | 0.40449 |
| Oryzarol | 0.41449 | 0.39496 | 0.74204 | 0.93126 | 1.04946 | 0.06965 | 0.39430 |
| Osmaronin | 0.05446 | 0.03890 | 0.46138 | 0.84543 | 1.40016 | 0.48559 | 0.13089 |
| Ovalicin | 0.12624 | 0.10619 | 0.44179 | 0.84346 | 1.18882 | 0.24953 | 0.20071 |
| Oxolan-3-one | 0.94030 | 1.16822 | 0.37070 | 0.82398 | 0.80490 | -0.31312 | 1.19808 |
| Oxypinnatanine | 0.04240 | 0.08062 | 0.24196 | 0.80583 | 0.52589 | -0.92716 | 1.39684 |
| Palmitic amide | 0.20388 | 0.25567 | 0.45744 | 0.84543 | 0.79744 | -0.32655 | 1.42323 |
| Palmitoleoyl Ethanolamide | 0.16224 | 0.21697 | 0.21878 | 0.80583 | 0.74773 | -0.41941 | 1.35602 |
| Palmitoylethanolamide | 2.76285 | 4.74864 | 0.08852 | 0.76203 | 0.58182 | -0.78136 | 1.36583 |
| Pantothenic acid | 0.69880 | 0.87186 | 0.40656 | 0.82398 | 0.80150 | -0.31922 | 0.87624 |
| Paucine | 0.09063 | 0.10576 | 0.73405 | 0.92959 | 0.85687 | -0.22286 | 0.84648 |
| PC(15:0/15:0) | 0.06046 | 0.03422 | 0.24455 | 0.80583 | 1.76698 | 0.82128 | 1.15138 |
| PC(16:0/15:0) | 0.05881 | 0.01307 | 0.17518 | 0.80583 | 4.49998 | 2.16992 | 1.29190 |
| PC(16:0/16:0) | 0.25556 | 0.13727 | 0.08560 | 0.75235 | 1.86174 | 0.89665 | 1.63819 |
| PC(18:1(11Z)/14:0) | 0.04645 | 0.05452 | 0.63039 | 0.91307 | 0.85191 | -0.23123 | 0.29679 |
| PC(18:2(9Z,12Z)/18:0) | 12.94914 | 8.46365 | 0.16080 | 0.80583 | 1.52997 | 0.61350 | 0.92900 |
| PC(18:3(6Z,9Z,12Z)/18:1(11Z)) | 0.25740 | 0.54743 | 0.41882 | 0.82772 | 0.47020 | -1.08866 | 0.62830 |
| PC(20:1(11Z)/14:0) | 0.84999 | 1.29055 | 0.50941 | 0.86434 | 0.65863 | -0.60247 | 0.28725 |
| PC(20:2(11Z,14Z)/14:0) | 0.48665 | 0.73038 | 0.59845 | 0.91307 | 0.66630 | -0.58575 | 0.29123 |
| PC(20:3(5Z,8Z,11Z)/16:0) | 0.37428 | 1.38694 | 0.25306 | 0.80583 | 0.26986 | -1.88972 | 0.77097 |
| PC-M6 | 0.27635 | 0.22875 | 0.30156 | 0.82070 | 1.20805 | 0.27268 | 0.78288 |
| PC(P-16:0/20:1(11Z)) | 0.17179 | 0.19794 | 0.73406 | 0.92959 | 0.86788 | -0.20443 | 0.05694 |
| PC(P-18:1(11Z)/16:0) | 0.05707 | 0.07085 | 0.61136 | 0.91307 | 0.80545 | -0.31214 | 0.17727 |
| PE(15:0/14:0) | 0.07513 | 0.06936 | 0.82180 | 0.94058 | 1.08308 | 0.11514 | 0.91184 |
| PE(15:0/16:0) | 0.16347 | 0.11319 | 0.37269 | 0.82398 | 1.44414 | 0.53021 | 1.55862 |
| PE(16:0/14:0) | 0.28287 | 0.19427 | 0.40355 | 0.82398 | 1.45612 | 0.54213 | 1.29859 |
| Pentanenitrile | 1.23556 | 1.12121 | 0.70410 | 0.92441 | 1.10199 | 0.14011 | 0.69726 |
| PE(P-18:1(11Z)/14:1(9Z)) | 0.06303 | 0.01243 | 0.03114 | 0.75227 | 5.06921 | 2.34176 | 1.89299 |
| Phaeophorbide b | 0.04616 | 0.10782 | 0.34121 | 0.82398 | 0.42814 | -1.22384 | 0.79377 |
| Phenethylamine glucuronide | 2.44632 | 0.66490 | 0.31885 | 0.82125 | 3.67921 | 1.87939 | 0.32235 |
| Phenmetrazine | 0.12667 | 0.17663 | 0.52539 | 0.87218 | 0.71718 | -0.47959 | 1.18647 |
| Phenylacetaldehyde | 3.95428 | 4.42732 | 0.82093 | 0.94058 | 0.89315 | -0.16302 | 0.26595 |
| Phenylalanyl-Alanine | 0.40058 | 0.36352 | 0.51703 | 0.86996 | 1.10195 | 0.14006 | 0.38059 |
| Phenylalanyl-Gamma-glutamate | 0.04790 | 0.06628 | 0.09561 | 0.79835 | 0.72266 | -0.46861 | 1.95080 |
| Phenylalanyl-Glutamine | 0.04205 | 0.06116 | 0.23261 | 0.80583 | 0.68743 | -0.54073 | 1.69473 |
| Phenylalanyl-Glycine | 0.20644 | 0.18909 | 0.64256 | 0.91307 | 1.09176 | 0.12665 | 0.21367 |
| Phenylalanyl-Isoleucine | 5.44396 | 5.88909 | 0.66139 | 0.91627 | 0.92442 | -0.11339 | 0.42488 |
| Phenylalanyl-Methionine | 0.32278 | 0.30783 | 0.84390 | 0.95251 | 1.04858 | 0.06843 | 0.04480 |
| Phenylalanylproline | 0.73142 | 0.74714 | 0.93190 | 0.98002 | 0.97896 | -0.03068 | 0.90334 |
| Phenylalanyl-Threonine | 0.19763 | 0.18508 | 0.65255 | 0.91576 | 1.06780 | 0.09464 | 0.03315 |
| Phenylalanyl-Tryptophan | 0.15153 | 0.16503 | 0.68772 | 0.92441 | 0.91817 | -0.12317 | 0.66080 |
| Phenylalanyl-Tyrosine | 0.26522 | 0.28161 | 0.75515 | 0.93126 | 0.94180 | -0.08650 | 0.75570 |
| Phenylalanyl-Valine | 2.49927 | 2.40937 | 0.82129 | 0.94058 | 1.03731 | 0.05285 | 0.22530 |
| Phenylethylamine | 1.12846 | 3.23728 | 0.04403 | 0.75227 | 0.34858 | -1.52043 | 1.68572 |
| Phosphorylcholine | 0.10243 | 0.04746 | 0.01531 | 0.73899 | 2.15818 | 1.10982 | 2.11393 |
| Physalolactone B | 0.05917 | 0.04162 | 0.07291 | 0.75227 | 1.42151 | 0.50742 | 1.09882 |
| Phytosphingosine | 4.39805 | 3.53943 | 0.24809 | 0.80583 | 1.24259 | 0.31335 | 0.93997 |
| PI(16:2(9Z,12Z)/22:3(10Z,13Z,16Z)) | 0.24235 | 0.18384 | 0.06002 | 0.75227 | 1.31825 | 0.39863 | 0.98745 |
| Picraquassioside A | 0.00774 | 0.02242 | 0.12511 | 0.80583 | 0.34509 | -1.53495 | 1.15819 |
| Pipecolic acid | 0.85848 | 0.81230 | 0.76716 | 0.93927 | 1.05686 | 0.07978 | 0.77954 |
| Pipericine | 0.21256 | 0.22117 | 0.70994 | 0.92626 | 0.96107 | -0.05729 | 0.72800 |
| Piperidine | 8.64144 | 9.28145 | 0.69349 | 0.92441 | 0.93104 | -0.10308 | 0.60620 |
| Pipermethystine | 0.03567 | 0.05818 | 0.31945 | 0.82125 | 0.61307 | -0.70588 | 1.45745 |
| Plantaricin BN | 0.05373 | 0.05749 | 0.85814 | 0.95530 | 0.93451 | -0.09771 | 0.80039 |
| p-Mentha-1,8-dien-7-ol | 0.42077 | 0.40930 | 0.82397 | 0.94058 | 1.02804 | 0.03989 | 0.30031 |
| Polyoxyethylene (600) monoricinoleate | 0.03327 | 0.08976 | 0.07936 | 0.75227 | 0.37068 | -1.43175 | 1.13242 |
| Polyporusterone C | 0.19175 | 0.07019 | 0.44986 | 0.84543 | 2.73191 | 1.44991 | 0.73959 |
| Porphobilinogen | 0.33012 | 0.22356 | 0.29845 | 0.82070 | 1.47663 | 0.56231 | 0.81176 |
| Prenyl glucoside | 0.07982 | 0.04622 | 0.47485 | 0.84543 | 1.72708 | 0.78833 | 0.98664 |

| Proline betaine | 9.20212 | 4.05742 | 0.00494 | 0.58958 | 2.26797 | 1.18140 | 1.99511 |
| --- | --- | --- | --- | --- | --- | --- | --- |
| Prolyl-Alanine | 0.92138 | 1.05147 | 0.43756 | 0.83798 | 0.87628 | -0.19054 | 1.20030 |
| Prolyl-Arginine | 0.47386 | 0.44474 | 0.84569 | 0.95251 | 1.06549 | 0.09152 | 0.65673 |
| Prolyl-Asparagine | 0.37769 | 0.29709 | 0.45741 | 0.84543 | 1.27127 | 0.34627 | 0.16349 |
| Prolyl-Aspartate | 0.08432 | 0.08820 | 0.83010 | 0.94562 | 0.95600 | -0.06492 | 0.86991 |
| Prolyl-Glutamine | 0.19917 | 0.20768 | 0.86558 | 0.95561 | 0.95901 | -0.06039 | 0.10841 |
| Prolylglycine | 0.19306 | 0.19297 | 0.99810 | 0.99977 | 1.00044 | 0.00063 | 0.58195 |
| Prolylhydroxyproline | 0.12123 | 0.16024 | 0.26882 | 0.81355 | 0.75658 | -0.40244 | 1.08064 |
| Prolyl-Methionine | 0.05123 | 0.04164 | 0.48379 | 0.84975 | 1.23042 | 0.29916 | 0.13785 |
| Prolyl-Threonine | 0.05689 | 0.06328 | 0.72641 | 0.92871 | 0.89907 | -0.15349 | 0.46892 |
| Prolyl-Tryptophan | 0.10965 | 0.12807 | 0.75731 | 0.93126 | 0.85612 | -0.22412 | 0.57514 |
| Prolyl-Valine | 0.48766 | 0.58730 | 0.36302 | 0.82398 | 0.83034 | -0.26822 | 1.43343 |
| Prostaglandin E2 | 0.24614 | 0.12557 | 0.08196 | 0.75227 | 1.96012 | 0.97094 | 1.47773 |
| Prostaglandin H2 2-glyceryl Ester | 0.17455 | 0.16854 | 0.84694 | 0.95251 | 1.03564 | 0.05052 | 0.23780 |
| Protoporphyrin IX | 0.81118 | 0.63700 | 0.69988 | 0.92441 | 1.27344 | 0.34873 | 0.39610 |
| Pseudoionone | 0.08277 | 0.07359 | 0.43010 | 0.83108 | 1.12474 | 0.16960 | 0.52576 |
| Pyridoxal | 0.24929 | 0.31567 | 0.37640 | 0.82398 | 0.78971 | -0.34061 | 1.25047 |
| Pyridoxamine | 0.68344 | 0.67771 | 0.97897 | 0.99353 | 1.00847 | 0.01216 | 0.01472 |
| Pyridoxine | 0.41223 | 0.44616 | 0.74829 | 0.93126 | 0.92394 | -0.11413 | 0.38991 |
| Pyrimidine | 0.22809 | 0.19079 | 0.15788 | 0.80583 | 1.19549 | 0.25761 | 0.92661 |
| Pyroglutamic acid | 1.30055 | 1.73888 | 0.32670 | 0.82398 | 0.74793 | -0.41903 | 1.68827 |
| Pyro-L-glutaminyl-L-glutamine | 0.25220 | 0.13765 | 0.42155 | 0.82772 | 1.83219 | 0.87357 | 0.34337 |
| Pyrophaeophorbide a | 2.26430 | 1.63318 | 0.36835 | 0.82398 | 1.38644 | 0.47139 | 0.60697 |
| Pyrraline | 0.20016 | 0.19406 | 0.93578 | 0.98163 | 1.03141 | 0.04462 | 0.41534 |
| Pyrrolidonecarboxylic acid | 4.03909 | 3.79098 | 0.72739 | 0.92871 | 1.06545 | 0.09146 | 0.48008 |
| Quinaldine | 1.28489 | 1.05952 | 0.60922 | 0.91307 | 1.21270 | 0.27822 | 0.49235 |
| (R)-3,4-Dihydro-2-methyl-2-(4,8,12-trimethyl-3,7,11-tridecatrienyl)-2H-1-benzopyr | 0.06909 | 0.07824 | 0.58385 | 0.90116 | 0.88299 | -0.17953 | 1.23096 |
| R-95913 | 0.13135 | 0.16627 | 0.74517 | 0.93126 | 0.79001 | -0.34006 | 0.67032 |
| Raffinose | 0.00802 | 0.00930 | 0.58068 | 0.89986 | 0.86162 | -0.21488 | 0.44886 |
| Rheidin A | 0.03107 | 0.02191 | 0.29062 | 0.81978 | 1.41835 | 0.50422 | 0.56535 |
| Riboflavin | 0.48414 | 0.96194 | 0.22539 | 0.80583 | 0.50330 | -0.99050 | 1.11295 |
| Rishitin | 0.08749 | 0.05310 | 0.11680 | 0.80583 | 1.64766 | 0.72042 | 0.95488 |
| Rosmic acid | 0.16702 | 0.11770 | 0.31100 | 0.82125 | 1.41898 | 0.50486 | 0.51361 |
| Rotundine B | 0.09885 | 0.12433 | 0.28004 | 0.81613 | 0.79508 | -0.33082 | 0.55380 |
| Saccharopine | 0.07816 | 0.09023 | 0.73477 | 0.92959 | 0.86624 | -0.20717 | 0.35736 |
| S-Acetyldihydrolipoamide | 0.27568 | 0.30126 | 0.74571 | 0.93126 | 0.91508 | -0.12803 | 1.28757 |
| S-Adenosylmethionine | 0.04298 | 0.04587 | 0.85115 | 0.95269 | 0.93704 | -0.09382 | 0.35433 |
| Sakacin P | 0.18317 | 0.21081 | 0.47078 | 0.84543 | 0.86889 | -0.20276 | 1.03820 |
| Santalyl phenylacetate | 0.03268 | 0.03787 | 0.60597 | 0.91307 | 0.86278 | -0.21294 | 1.01149 |
| Santene | 0.26435 | 0.25629 | 0.80005 | 0.94058 | 1.03146 | 0.04469 | 0.27203 |
| Saxitoxin | 0.07574 | 0.03570 | 0.18707 | 0.80583 | 2.12132 | 1.08496 | 0.58229 |
| Sciadonic acid | 0.15429 | 0.13248 | 0.47447 | 0.84543 | 1.16461 | 0.21985 | 0.60403 |
| Semilepidinoside A | 0.02489 | 0.02820 | 0.63582 | 0.91307 | 0.88256 | -0.18024 | 1.11153 |
| Serotonin | 0.08527 | 0.21576 | 0.00709 | 0.62473 | 0.39520 | -1.33933 | 1.95941 |
| Serylalanine | 0.17900 | 0.18266 | 0.92594 | 0.97743 | 0.97994 | -0.02924 | 0.22593 |
| Serylglycine | 0.05950 | 0.05258 | 0.47613 | 0.84543 | 1.13166 | 0.17844 | 0.47110 |
| Serylisoleucine | 0.11544 | 0.23453 | 0.03251 | 0.75227 | 0.49224 | -1.02258 | 2.04604 |
| Seryltryptophan | 0.02017 | 0.01865 | 0.77332 | 0.93930 | 1.08169 | 0.11328 | 0.16645 |
| Serylvaline | 0.61268 | 0.59647 | 0.86749 | 0.95561 | 1.02718 | 0.03868 | 0.27717 |
| (S,E)-Zearalenone | 0.17169 | 0.19322 | 0.68549 | 0.92441 | 0.88858 | -0.17043 | 0.09774 |
| SM(d18:1/16:0) | 0.35540 | 0.26929 | 0.32782 | 0.82398 | 1.31975 | 0.40026 | 1.04208 |
| Smilagenone | 0.40501 | 0.35704 | 0.72374 | 0.92871 | 1.13437 | 0.18189 | 0.08740 |
| Solanidine | 0.40548 | 0.72505 | 0.47727 | 0.84543 | 0.55925 | -0.83844 | 0.67479 |
| Solanocardinol | 0.05461 | 0.05266 | 0.94593 | 0.98827 | 1.03696 | 0.05236 | 0.68859 |
| Solasodine | 0.25298 | 0.11169 | 0.22578 | 0.80583 | 2.26510 | 1.17958 | 0.75735 |
| Sphinganine | 18.83614 | 17.95945 | 0.88194 | 0.95744 | 1.04881 | 0.06876 | 0.20111 |
| Sphingosine | 23.37644 | 19.50284 | 0.58387 | 0.90116 | 1.19862 | 0.26137 | 0.24500 |
| Squamolone | 0.55760 | 1.17309 | 0.23495 | 0.80583 | 0.47533 | -1.07300 | 0.43362 |
| Stachyose | 0.03551 | 0.01666 | 0.38843 | 0.82398 | 2.13100 | 1.09153 | 0.07569 |
| Stearoylcarnitine | 0.78615 | 1.64218 | 0.15661 | 0.80583 | 0.47872 | -1.06273 | 1.47676 |
| Stearoylethanolamide | 1.19461 | 1.17973 | 0.98226 | 0.99538 | 1.01262 | 0.01809 | 0.03166 |
| Stigmast-4-ene-3,6-dione | 2.28707 | 2.82175 | 0.33751 | 0.82398 | 0.81051 | -0.30309 | 1.26893 |
| Stigmasterol | 0.01214 | 0.01349 | 0.66717 | 0.91627 | 0.90002 | -0.15197 | 0.83756 |
| Styrene | 1.43833 | 4.18098 | 0.04200 | 0.75227 | 0.34402 | -1.53945 | 1.69726 |
| Suberylglycine | 0.15155 | 0.22062 | 0.17531 | 0.80583 | 0.68694 | -0.54174 | 1.21056 |
| Sucrose | 0.09598 | 0.03042 | 0.37314 | 0.82398 | 3.15576 | 1.65799 | 0.36384 |
| Taurine | 5.56304 | 6.43373 | 0.71225 | 0.92637 | 0.86467 | -0.20978 | 1.18995 |
| Taurodeoxycholic acid | 0.17314 | 0.06958 | 0.37726 | 0.82398 | 2.48823 | 1.31512 | 0.60022 |
| Tetraacetylethylenediamine | 0.41154 | 0.37215 | 0.66620 | 0.91627 | 1.10586 | 0.14517 | 0.00347 |
| Tetradecanoylcarnitine | 0.21933 | 0.26812 | 0.63067 | 0.91307 | 0.81801 | -0.28982 | 0.35369 |
| Tetraphyllin B | 0.07722 | 0.04056 | 0.47772 | 0.84543 | 1.90378 | 0.92887 | 0.48673 |
| Thiamine | 2.81580 | 6.70573 | 0.34349 | 0.82398 | 0.41991 | -1.25185 | 1.14990 |
| Thiomorpholine 3-carboxylate | 1.01601 | 1.15701 | 0.40590 | 0.82398 | 0.87813 | -0.18749 | 0.55931 |
| Threoninyl-Aspartate | 0.10526 | 0.11553 | 0.61214 | 0.91307 | 0.91110 | -0.13432 | 0.75905 |
| Threoninyl-Leucine | 0.07045 | 0.03941 | 0.12994 | 0.80583 | 1.78762 | 0.83804 | 1.27730 |
| Threoninyl-Tyrosine | 0.10145 | 0.09516 | 0.67067 | 0.91945 | 1.06616 | 0.09243 | 0.15120 |
| Thujyl 19-trachylobanoate | 0.14826 | 0.08425 | 0.06893 | 0.75227 | 1.75978 | 0.81540 | 1.89777 |
| Thymine | 0.35407 | 0.31058 | 0.60309 | 0.91307 | 1.14003 | 0.18907 | 0.11877 |
| Thyrotropin releasing hormone | 0.02313 | 0.02456 | 0.81055 | 0.94058 | 0.94163 | -0.08676 | 1.13641 |
| Tiglylglycine | 0.10375 | 0.18107 | 0.10396 | 0.80583 | 0.57297 | -0.80346 | 1.97889 |
| trans-Hexadec-2-enoyl carnitine | 0.37820 | 0.45278 | 0.63205 | 0.91307 | 0.83528 | -0.25967 | 0.25665 |
| trans-S-(1-Propenyl)-L-cysteine | 2.73865 | 5.86020 | 0.21814 | 0.80583 | 0.46733 | -1.09749 | 0.70022 |
| Trehalose | 0.18655 | 0.17143 | 0.72660 | 0.92871 | 1.08819 | 0.12193 | 0.70840 |
| Triacetin | 0.31059 | 0.02482 | 0.33066 | 0.82398 | 12.51298 | 3.64535 | 0.47295 |
| Tridemorph | 0.39797 | 0.35897 | 0.72246 | 0.92871 | 1.10865 | 0.14880 | 0.04086 |
| Triethanolamine | 0.75915 | 0.16633 | 0.18327 | 0.80583 | 4.56400 | 2.19030 | 1.35903 |
| Trimethylamine N-oxide | 0.04080 | 0.03334 | 0.27004 | 0.81355 | 1.22373 | 0.29128 | 0.00090 |
| Trimethylaminoacetone | 0.09348 | 0.10117 | 0.72945 | 0.92959 | 0.92392 | -0.11415 | 0.97523 |
| Tryptamine | 0.36806 | 0.86385 | 0.01454 | 0.73899 | 0.42607 | -1.23084 | 2.03288 |

| Tryptophyl-Alanine | 0.02142 | 0.02009 | 0.71648 | 0.92871 | 1.06579 | 0.09192 | 0.55807 |
| --- | --- | --- | --- | --- | --- | --- | --- |
| Tryptophyl-Arginine | 0.14885 | 0.08177 | 0.05283 | 0.75227 | 1.82043 | 0.86428 | 1.61721 |
| Tryptophyl-Histidine | 0.03901 | 0.02337 | 0.57000 | 0.89551 | 1.66953 | 0.73944 | 0.41042 |
| Tryptophyl-Tryptophan | 0.10443 | 0.15341 | 0.44547 | 0.84346 | 0.68069 | -0.55493 | 0.38189 |
| Tsangane L 3-glucoside | 0.08963 | 0.09046 | 0.96079 | 0.98827 | 0.99080 | -0.01333 | 0.45628 |
| Tyramine-O-sulfate | 0.08130 | 0.03383 | 0.48731 | 0.84975 | 2.40286 | 1.26475 | 0.04393 |
| Tyrosyl-Isoleucine | 0.05133 | 0.04666 | 0.75131 | 0.93126 | 1.10028 | 0.13787 | 0.65590 |
| Tyrosyl-Leucine | 0.38651 | 0.24419 | 0.55014 | 0.88284 | 1.58283 | 0.66250 | 0.14874 |
| Tyrosyl-Proline | 0.16185 | 0.14223 | 0.66305 | 0.91627 | 1.13795 | 0.18644 | 0.48012 |
| Tyrosyl-Serine | 0.37921 | 0.21091 | 0.12619 | 0.80583 | 1.79793 | 0.84634 | 1.30258 |
| Tyrosyl-Threonine | 0.04411 | 0.03447 | 0.34248 | 0.82398 | 1.27987 | 0.35599 | 0.13899 |
| Tyrosyl-Tyrosine | 0.04464 | 0.08083 | 0.27756 | 0.81613 | 0.55234 | -0.85636 | 1.19596 |
| Tyrosyl-Valine | 0.14264 | 0.18651 | 0.34988 | 0.82398 | 0.76480 | -0.38684 | 1.57310 |
| Ubiquinone-2 | 0.06192 | 0.10289 | 0.23920 | 0.80583 | 0.60177 | -0.73272 | 1.41870 |
| Ureidoisobutyric acid | 2.22734 | 2.93385 | 0.34239 | 0.82398 | 0.75919 | -0.39747 | 1.63839 |
| Urocanic acid | 4.72570 | 3.84527 | 0.32417 | 0.82125 | 1.22897 | 0.29744 | 0.10642 |
| Urothion | 0.66005 | 0.79330 | 0.26853 | 0.81355 | 0.83203 | -0.26529 | 1.86426 |
| Ursodeoxycholic acid | 0.93593 | 0.53701 | 0.06600 | 0.75227 | 1.74285 | 0.80145 | 1.22251 |
| Uzarigenin 3-[xylosyl-(1->2)-rhamnoside] | 0.66262 | 0.52699 | 0.65415 | 0.91627 | 1.25737 | 0.33041 | 1.13368 |
| Valerylglycine | 0.54358 | 0.30032 | 0.37026 | 0.82398 | 1.81000 | 0.85599 | 0.33018 |
| Valyl-Asparagine | 0.21128 | 0.25106 | 0.32457 | 0.82125 | 0.84153 | -0.24891 | 1.22835 |
| Valyl-Aspartate | 0.29433 | 0.17692 | 0.38325 | 0.82398 | 1.66360 | 0.73431 | 0.84161 |
| Valyl-Lysine | 2.23944 | 1.99372 | 0.62735 | 0.91307 | 1.12325 | 0.16768 | 0.39885 |
| Valyl-Phenylalanine | 0.10371 | 0.02807 | 0.31461 | 0.82125 | 3.69426 | 1.88529 | 0.49546 |
| Valyl-Proline | 0.32449 | 0.53682 | 0.19666 | 0.80583 | 0.60447 | -0.72625 | 1.03072 |
| Valyl-Valine | 5.71915 | 5.26410 | 0.64912 | 0.91556 | 1.08644 | 0.11961 | 0.12591 |
| Vitamin A | 0.13876 | 0.14633 | 0.86674 | 0.95561 | 0.94828 | -0.07662 | 0.41973 |
| Wampetin | 0.12150 | 0.04158 | 0.47300 | 0.84543 | 2.92199 | 1.54695 | 0.28227 |
| Wharangin | 0.39867 | 0.87938 | 0.46750 | 0.84543 | 0.45335 | -1.14129 | 1.12601 |
| Withanolide B | 0.52630 | 0.28240 | 0.18702 | 0.80583 | 1.86366 | 0.89814 | 1.00652 |
| (x)-1,2-Propanediol 1-O-b-D-glucopyranoside | 0.10910 | 0.06771 | 0.16138 | 0.80583 | 1.61117 | 0.68811 | 1.16858 |
| Xanthine | 0.39575 | 0.30951 | 0.67867 | 0.92441 | 1.27863 | 0.35459 | 0.26731 |
| Xanthosine | 0.03931 | 0.02297 | 0.01057 | 0.67889 | 1.71137 | 0.77516 | 1.61194 |
| xi-2,5-Dihydro-2,4-dimethylthiazole | 0.24550 | 0.43224 | 0.23844 | 0.80583 | 0.56797 | -0.81611 | 0.79009 |
| xi-2-Hydroxy-1,3,5-bisabolatrien-9-one | 0.30868 | 0.16825 | 0.03949 | 0.75227 | 1.83463 | 0.87549 | 1.40160 |
| Yuccaol C | 0.02175 | 0.02068 | 0.87230 | 0.95712 | 1.05174 | 0.07277 | 0.58699 |
| Zanthodioline | 0.01547 | 0.01978 | 0.24759 | 0.80583 | 0.78204 | -0.35469 | 1.58203 |
| Zymonic acid | 0.38372 | 0.37857 | 0.94336 | 0.98710 | 1.01360 | 0.01949 | 0.59942 |

**

**

**Fig. S1.** Correlations between clinical parameters conducted by Pearson’s correlation analyses.





**Fig. S2.**Differentiated fecal microbial diversity in three groups. β diversity calculated between HC and SP (a), SP, and AE (b), HC and AE (c) calculated using unweighted UniFrac by PCoA.


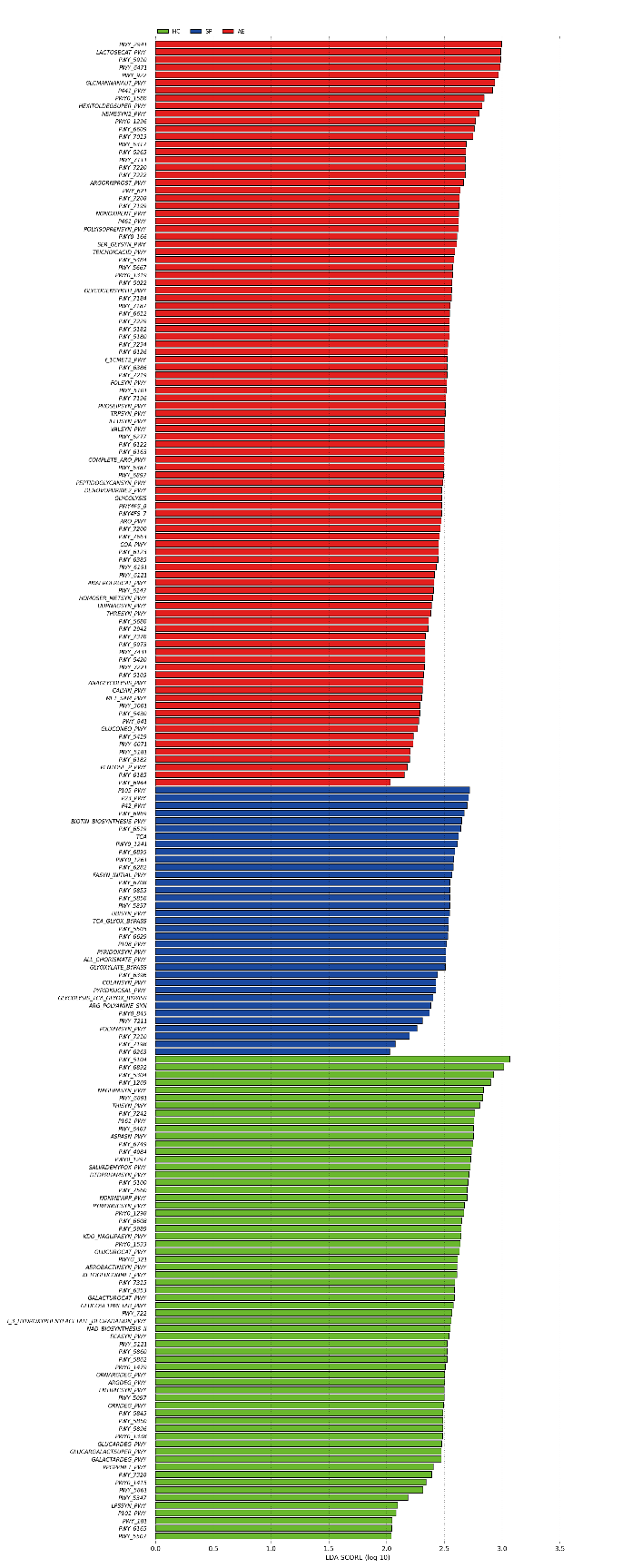


**Fig. S3.** Statistical LEfSe analysis of differential MetaCyc_pathway diagram at *P* < 0.05 and LDA scores (log10) > 2.


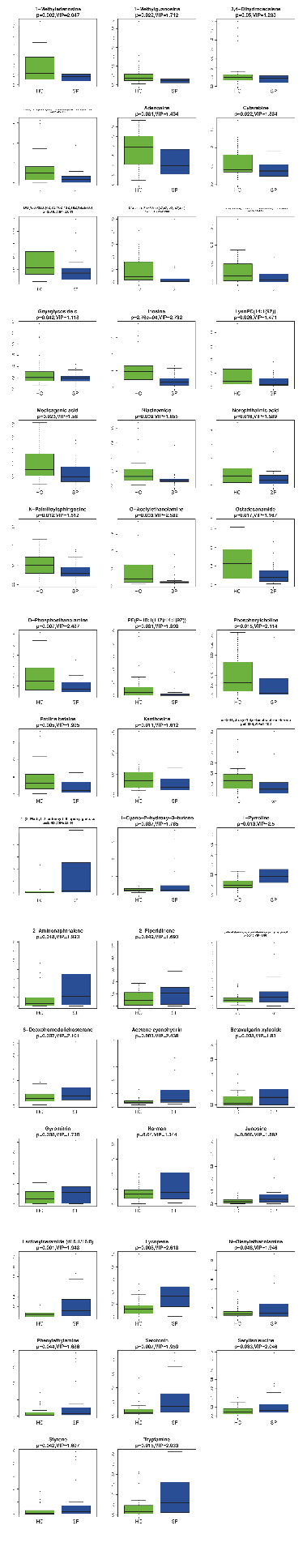


**Fig. S4.**Forty-four differential fecal metabolites in the HC and SP groups.


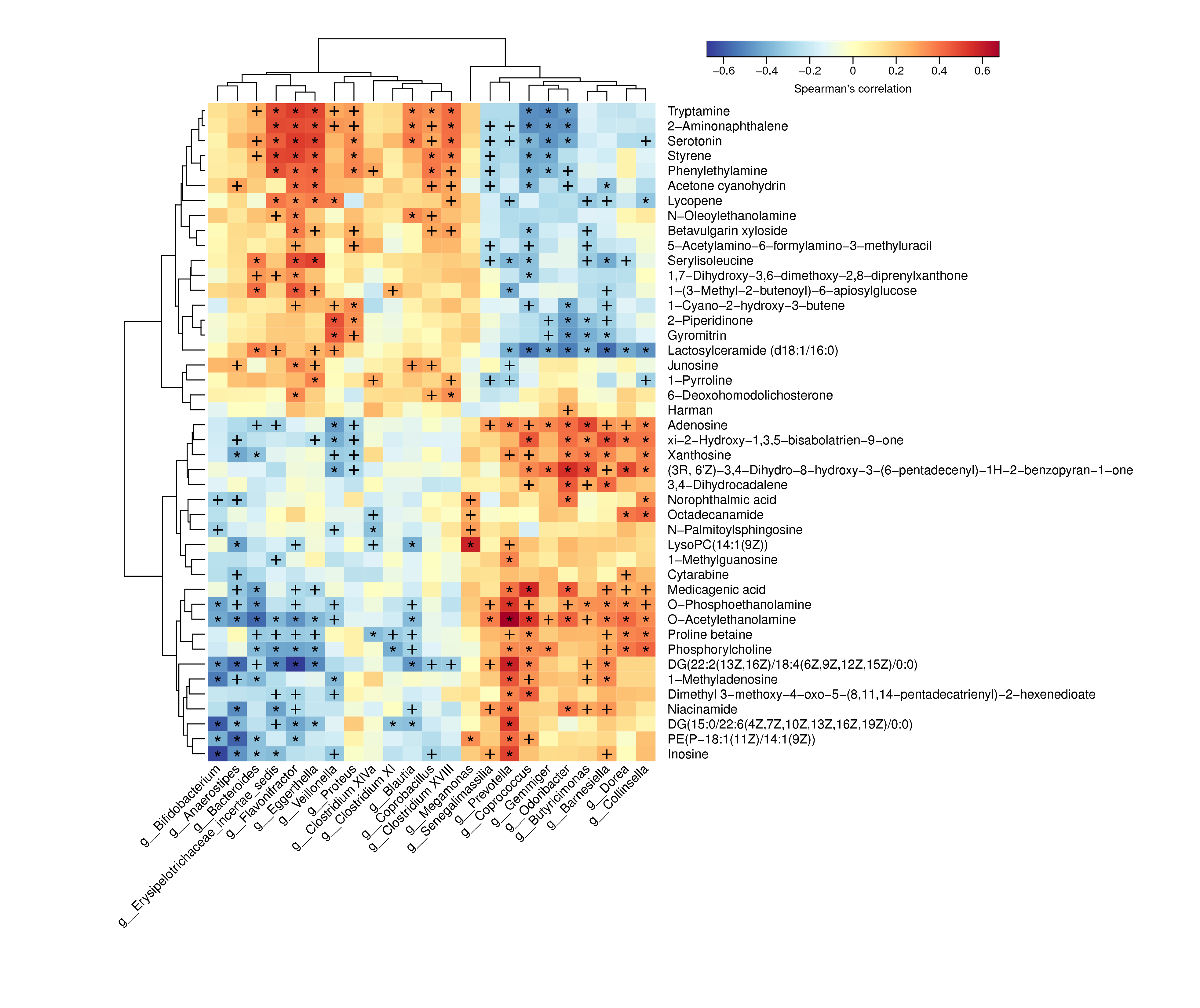


**Fig. S5.**Correlations between the 23 differential genera and 44 metabolites by Spearman’s rank correlation coefficient analyses. +*P*<0.05, **P* <0.01.


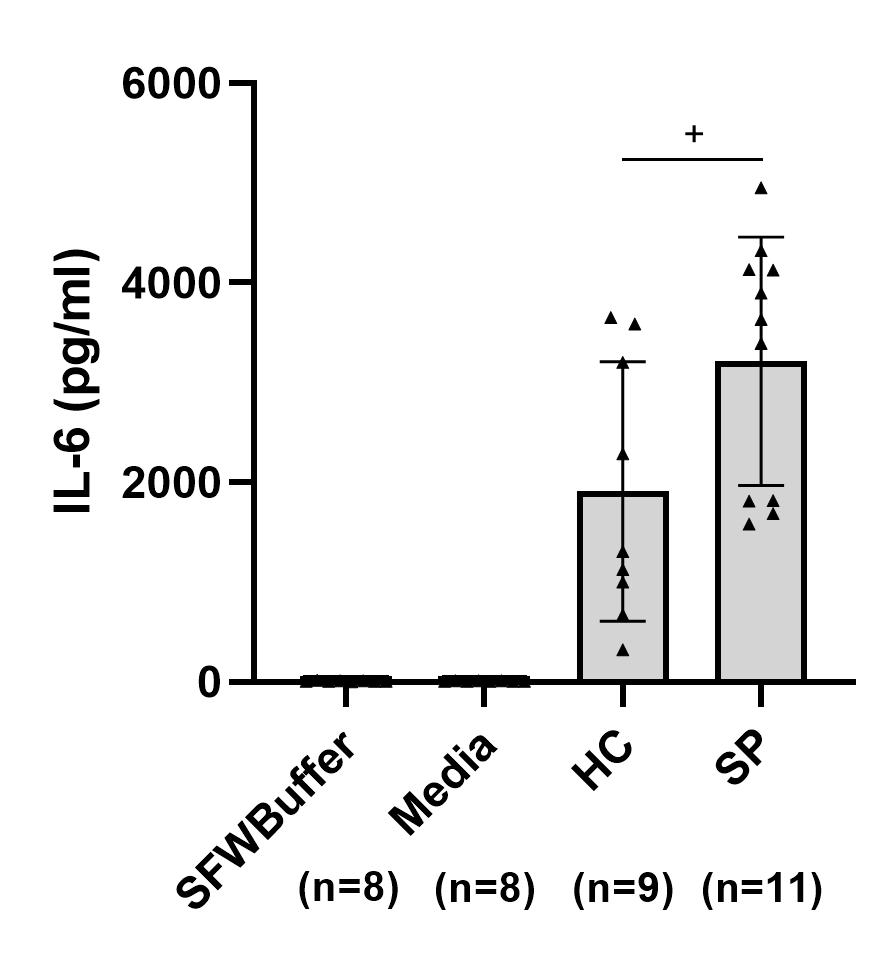


**Fig. S6.**IL-6 levels in the supernatant of SFW-cultured MH-S cells detected by ELISA. +*P*<0.05.

**Additional Methods**

Stool samples were homogenized 1g/ml with pre-warmed SFW buffer (phosphate-buffered saline (PBS) containing 20% heat-inactivated fetal bovine serum (FBS)). Samples were then thoroughly vortexed, incubated in a 37℃ water bath for 10min, and centrifuged at 14000×g for 10 minutes. Supernatant was centrifuged at 16000×g for another 1 hour. Supernatant was transferred to a new tube, and was successively filter-sterilized though a 0.45μm and a 0.22μm filter, and the filtrate was collected as sterile fecal water. The SFW buffer and media (media 1640 with 10% FBS and 100 U/ml penicillin–streptomycin) were used as negative controls in the preliminary experiments. The MH-S cells were then co-incubated with SFW for 24 hours at 37℃ within a cell incubator. Supernatant was then harvested to measure the concentration of IL-6.
